# Tandem four-component reaction for efficient synthesis of dihydrothiophene with substituted amino acid ethyl esters†

**DOI:** 10.1039/c8ra03605e

**Published:** 2018-06-20

**Authors:** Jing Sun, Yu Zhang, Chao-Guo Yan

**Affiliations:** College of Chemistry & Chemical Engineering, Yangzhou University Yangzhou 225002 China cgyan@yzu.edu.cn

## Abstract

The one-pot four-component reaction of aromatic aldehydes, malononitrile, 1,3-thiazolidinedione and ethyl glycinate hydrochloride in ethanol in the presence of triethylamine afforded *trans*-dihydrothiophene ureidoformamide derivatives in moderate to good yields. The other α-amino acid ethyl esters resulted in the corresponding diastereoisomeric dihydrothiophene derivatives with various molecular ratios. The functionalized thiophene derivatives were also successfully prepared by sequential dehydrogenation reaction with DDQ.

## Abstract 2

The one-pot four-component reaction of aromatic aldehydes, malononitrile, 1,3-thiazolidinedione and ethyl glycinate hydrochloride in ethanol in the presence of triethylamine afforded *trans*-dihydrothiophene.

## Graphical abstract

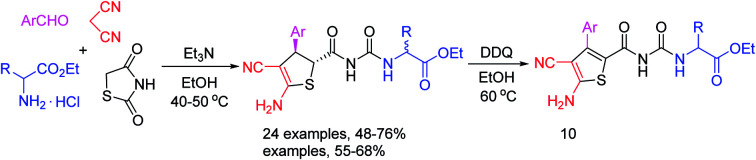

## 1 Introduction

The heterocyclic nucleus 2,3-dihydrothiophene is widely distributed in many natural products, bioactive compounds possessing useful medicinal and biological activities.^1–3^ On the other hand, 2,3-dihydrothiophenes are also useful synthetic precursors for many compounds including thiophenes, thionucleoside derivatives, and penicillin mimics.^4,5^ Therefore, the development of efficient synthetic methodologies for functionalized 2,3-dihydrothiophenes has attracted continuous interest in the field of organic and medicinal chemistry.^6,7^ Although many elegant strategies toward the construction of dihydrothiophenes have been successfully accessed, green approaches to functionalized dihydrothiophenes with high atomic efficiency and good feasibility to assemble various substitution patterns is still highly desirable.^8,9^ Recently, we successfully revealed a novel domino four-component reaction of 1,3-thiazolidinedione, malononitrile, aromatic aldehydes, and amines for efficient synthesis of dihydrothiophene derivatives. This reaction was very unique because the ring-opening/recyclization process occurs unexpectedly at the ring of 1,3-thiazolidinedione with various amines.^10^ In the past few years, for developing “greener” processes and ascertaining limitations of this useful transformation, this four-component reaction was also carried out in a functional ionic liquid, under ultrasound irradiation and under catalyst-free conditions as well as others.^11,12^ For further demonstrating the synthetic value of this four-component reaction, we successfully employed several α-amino acid ethyl ester hydrochlorides as the amino component, and herein we wish to report the efficient synthesis of dihydrothiophenes and thiophenes with substituted α-amino acid ethyl esters.

## 2 Results and discussions

**Fig. 1:**
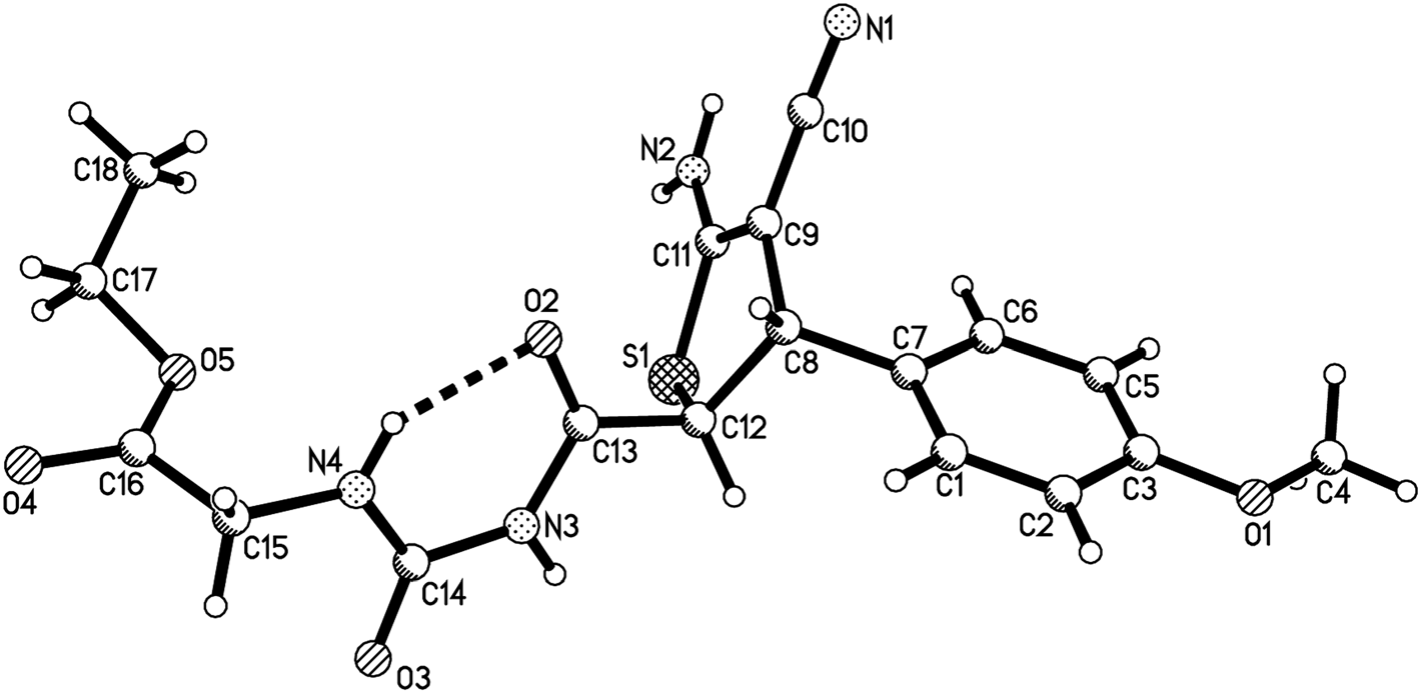
Crystal structure of the compound 1b.

Initially, a mixture of benzaldehyde, malononitrile, 1,3-thiazolidinedione and ethyl glycinate hydrochloride in ethanol in the presence of triethylamine was stirred at room temperature according to the previously established reaction conditions. The reaction resulted in a complicate mixture of products and the desired product 1a was only obtained in very low yields. Then, a one-pot two-step reaction procedure was employed. After carrying out the reaction of benzaldehyde with malononitrile in ethanol in the presence of triethylamine for about one hour, 1,3-thiazolidinedione and ethyl glycinate hydrochloride was added to the reaction system. Then, the reaction was conducted at 40–50 °C for about six hours. The substituted dihydrothiophene 1a could be prepared in 59% yield. Under this convenient one-pot two-step reaction procedure, other aromatic aldehydes were employed in the reaction and the corresponding dihydrothiophene derivatives 1b–1g were obtained in moderate to good yields (Table 1). The substituent on the aryl group showed little effect on the yields of the products. Thiophene-2-carbaldehyde can be used in the reaction to give the desired product 1 h in 60% yield. However, the similar reactions with aliphatic aldehydes always resulted in complicate mixture of products. The structures of the products 1a–1h were fully characterized by various spectroscopies. Because there are two asymmetric carbon atoms in the obtained dihydrothiophenes, both *cis*- and *trans*-isomers might be formed in the reaction. The ^1^H NMR spectra of the 1a–1h usually displayed one set of absorptions, which indicated that only one diastereoisomer existed in the obtained products. In order to determine the relative configuration of the dihydrothiophenes, the single crystal structures of the compounds 1b (Fig. 1), 1d and 1h (Fig. s1 and s2†) were successfully determined. It can be seen that they have *trans*-configuration, in which the aryl group and the ureido group stand at *trans*-positions in the ring of dihydrothiophene. A intramolecular H-bond is formed between the carbonyl group and amino group in the molecule. This result is concordance to our previously reported four-component reactions with various aliphatic or aromatic amines, in that only *trans*-1,2-disubstituted dihydrothiophene derivatives were predominately formed in the reaction.^10^

**Table 1:** Synthesis of substituted dihydrothiophenes 1a–1g^a^

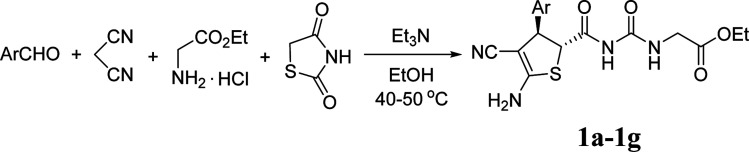			
Entry	Compd	Ar	Yield (%)^b^
1	1a	C_6_H_5_	59
2	1b	*p*-CH_3_OC_6_H_4_	75
3	1c	*o*-CH_3_OC_6_H_4_	73
4	1d	*p*-CH_3_C_6_H_4_	70
5	1e	*m*-CH_3_C_6_H_4_	64
6	1f	*p*-ClC_6_H_4_	76
7	1g	*m*-NO_2_C_6_H_4_	58
8	1h	2-Thiophene	60

a Reaction conditions: ArCHO (2.0 mmol), CH_2_(CN)_2_ (2.0 mmol), EtO_2_CCH_2_NH_3_Cl (2.0 mmol), 1,3-thiazolidinedione (2.0 mmol), Et_3_N (3.0 mmol), 40–50 °C, 6 h.

b Isolated yields.

**Fig. 2:**
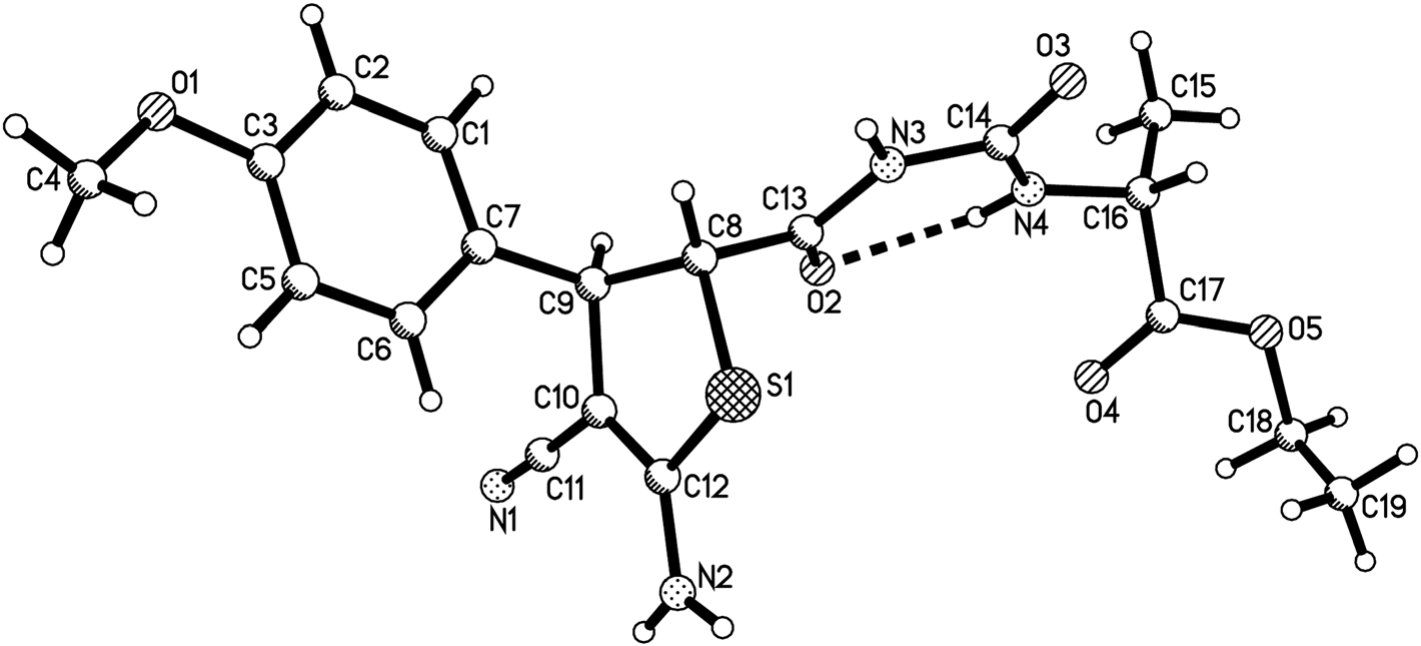
Crystal structure of the compound 2a.

**Scheme 1:**
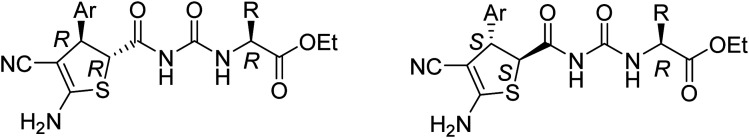
Illustration of two diastereoisomers for compounds 2a–2n.

In order to develop the scope of the reaction, various α-amino acid ethyl esters were employed in the four-component reaction. The results are summaries in Table 2. It can be seen that ethyl dl-alaninate, l-serine, l-isoleucinate, l-phenylalaninate, and l-hyperphenylalaninate gave the expected substituted dihydrothiophenes 2a–2n in moderate yields. The substituted dihydrothiophenes 2a–2n have two asymmetric carbon atoms in ring of dihydrothiophene and one chiral carbon atom in the scaffold of amino acid. ^1^H and ^13^C NMR spectra indicated that there is only one diastereoisomer in the products 2a–2f, which were derived from the reactions of dl-alaninate, l-serine, l-isoleucinate and l-phenylalaninate. However, there are two diastereoisomers with nearly 1 : 1 molar ratio existing in the products 2g–2n. The two diastereoisomers have very similar polarity and cannot be separated by column chromatography. The single crystal structures of the compounds 2a (Fig. 2), 2g, 2h and 2m (Fig. s3–s5†) were determined by X-ray diffraction. The two substituents on the ring of dihyrothiophene still exist on the *trans*-positions in four single crystals as that of the above products 1a–1h. Therefore, the two diastereoisomers were clearly come from the different relative configuration of the terminal substituted α-amino acid ethyl ester to the ring of dihydrothiophene (Scheme 1).

**Table 2:** Synthesis of substituted dihydrothiophenes 2a–2n^a^

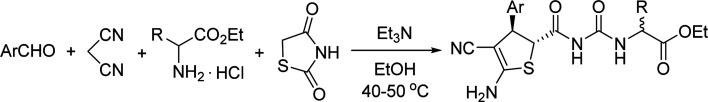				
Entry	Compd	Ar	R	Yield (%)^b^
1	2a	*p*-CH_3_OC_6_H_4_	Me	68
2	2b	*m*-NO_2_C_6_H_4_	Me	55
3	2c	*p*-CH_3_C_6_H_4_	CH_2_OH	63
4	2d	*p*-CH_3_OC_6_H_4_	*Sec*-Bu	68
5	2e	*p*-CH_3_C_6_H_4_	*Sec*-Bu	64
6	2f	C_6_H_5_	Bn	48
7	2g	*p*-CH_3_OC_6_H_4_	Bn	62 (53 : 47)
8	2h	*m*-CH_3_OC_6_H_4_	Bn	60 (51 : 49)
9	2i	*o*-CH_3_OC_6_H_4_	Bn	56 (51 : 49)
10	2j	*p*-CH_3_C_6_H_4_	Bn	55 (52 : 48)
11	2k	*p*-ClC_6_H_4_	Bn	53 (53 : 47)
12	2l	*p*-BrC_6_H_4_	Bn	55 (53 : 47)
13	2m	*p*-CH_3_OC_6_H_4_	CH_2_CH_2_Ph	67 (54 : 46)
14	2n	*p*-CH_3_OC_6_H_4_	CH_2_C_6_H_5_OH-*p*	57 (55 : 45)

a Reaction conditions: ArCHO (2.0 mmol), CH_2_(CN)_2_ (2.0 mmol), α-amino acid ethyl esters (2.0 mmol), 1,3-thiazolidinedione (2.0 mmol), Et_3_N (3.0 mmol), 40–50 °C, 6 h.

b Isolated yields.

**Fig. 3:**
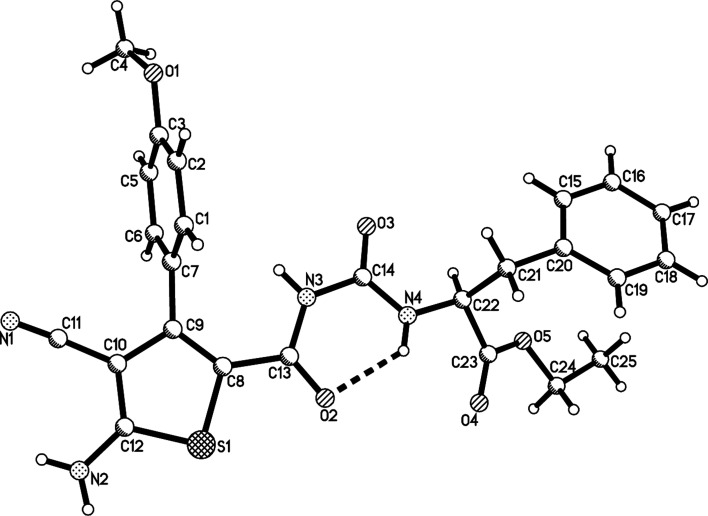
Crystal structure of the compound 3a.

For demonstrating the synthetic values of the four-component reaction, the aromatization of the obtained dihydrothiophenes was performed. When the four-component reaction in the presence of triethylamine was finished, the dehydrogenation reaction with a lightly excess of DDQ was carried out at elevated temperature. After workup, the corresponding thiophene derivatives 3a–3j were prepared in good yields. Due to formation of aromatized thiophene derivatives, ^1^H and ^13^C NMR spectra clearly showed one set of the characterized absorptions, which also indicated that there is only one diastereoisomer in the products 3a–3j. The single crystal structures of the compounds 3a (Fig. 3), 3d, 3g (Fig. s5 and s6†) were determined by X-ray diffraction method (Table 3).

**Table 3:** Synthesis of thiophene derivatives 3a–3j^a^

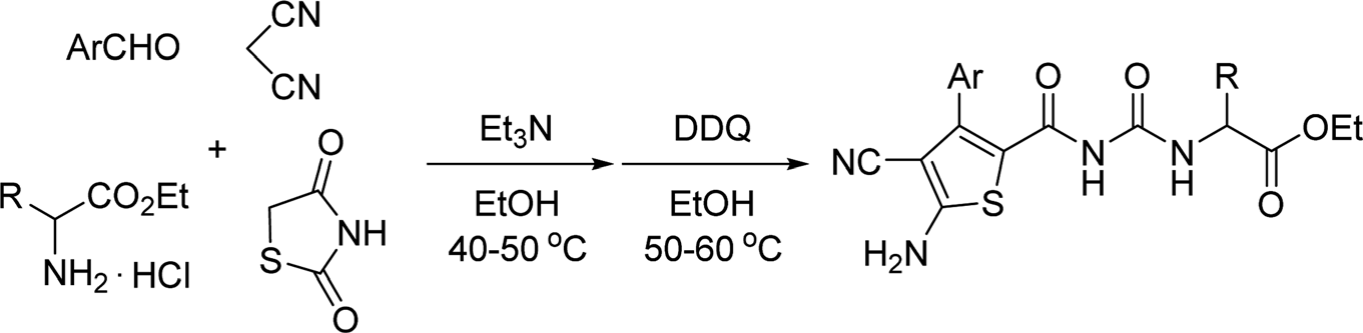				
Entry	Compd	Ar	R	Yield (%)^b^
1	3a	*p*-CH_3_OC_6_H_4_	Bn	62
2	3b	*m*-CH_3_OC_6_H_4_	Bn	60
3	3c	*o*-CH_3_OC_6_H_4_	Bn	60
4	3d	*p*-CH_3_C_6_H_4_	Bn	55
5	3e	*p*-ClC_6_H_4_	Bn	63
6	3f	*p*-BrC_6_H_4_	Bn	55
7	3g	*p*-CH_3_C_6_H_4_	H	60
8	3h	*p*-CH_3_OC_6_H_4_	Me	68
9	3i	*p*-CH_3_OC_6_H_4_	CH_2_CH_2_Ph	58
10	3j	*p*-CH_3_OC_6_H_4_	CH_2_C_6_H_4_OH-*p*	66

a Reaction conditions: 1. ArCHO (2.0 mmol), CH_2_(CN)_2_ (2.0 mmol), α-amino acid ethyl ester (2.0 mmol), 1,3-thiazolidinedione (2.0 mmol), Et_3_N (3.0 mmol), 40–50 °C, 6 h; 2. DDQ (2.2 mmol), 60 °C, 4 h.

b Isolated yields.

**Scheme 2:**
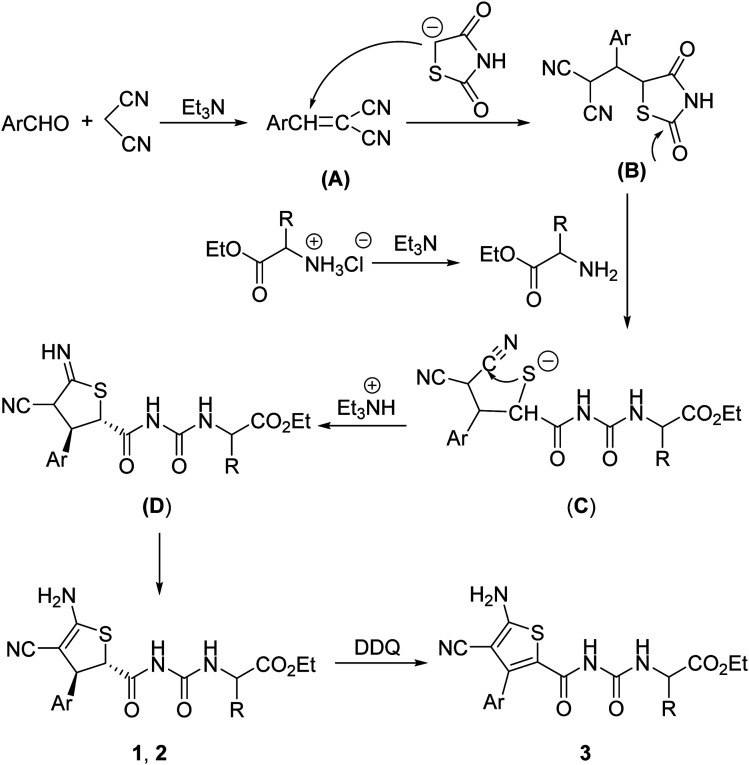
Proposed reaction mechanism for the four-component reaction.

For explaining the formation of the dihydrothiophene derivatives, a plausible reaction mechanism was proposed on the basis of the previously reported reactions.^10^ Firstly, the base catalyzed condensation reaction of aromatic aldehyde with malononitrile afforded arylidene malononitrile (A). Secondly, Michael addition of the *in situ* generated carbanion of 1,3-thiazolidinedione resulted in the adduct (B). In the meantime, free α-amino acid ethyl ester was produced by the neutralization of triethylamine with α-amino acid ethyl ester hydrochloride. Then the nucleophilic attack of amino group to the carbonyl group of intermediate (B) resulted in the ring-opening sulfide anion (C). The intramolecular nucleophilic addition of sulfide anion to one cyano group with sequential protonation gave the imino-substituted dihydrothiophene (D), which in turn converted to the dihydrothiophene derivatives 1 or 2 through imino–enamino tautomerization. The thermodynamically stable *trans*-isomer was predominately formed in the cyclization process. At last, DDQ oxidation of dihydrophene resulted in the thiophene product 3 (Scheme 2).

## 3 Conclusion

In summary, we have investigated the base promoted four-component reaction of aromatic aldehydes, malononitrile, 1,3-thiazolidinedion and α-amino acid ethyl ester hydrochlorides and provided efficient synthetic protocol for *trans*-2,3-dihydrothiophene and corresponding thiophene derivatives with scaffold of α-amino acid ethyl esters. This reaction has the advantages of using readily available chemicals, mild reaction conditions, satisfactory yields, high diastereoselectivity and atomic economy. More importantly, this reaction not only developed the variety of our previously established novel four-component reaction, but also further demonstrated the synthetic applications for diverse dihydrothiophene and thiophene derivatives. The potential application of this multicomponent reaction in organic and medicinal chemistry might be significant.

## 4 Experimental section

### 4.1. General procedure for the preparation of the dihydrothiophene derivatives 1a–1g and 2a–2m

A mixture of aromatic aldehyde (2.0 mmol) and malononitrile (2.0 mmol) and triethylamine (3.0 mmol) in ethanol (20.0 mL) was stirred at room temperature for one hour. Then, 1,3-thiazolidinedione (2.0 mmol) and α-amino acid ethyl ester hydrochloride (2.0 mmol) was added. The resulting mixture was stirred at about 40–50 °C for six hours. After removing the solvent by rotatory evaporation at reduced pressure, the residue was subjected to column chromatography with light petroleum and ethyl acetate (v/v = 2 : 1) as eluent to give pure product for analysis.

#### 4.1.1 Ethyl ((5-amino-4-cyano-3-phenyl-2,3-dihydrothiophene-2-carbonyl)carbamoyl)glycinate (1a)

Yellow solid, 49%, mp 188–190 °C; ^1^H NMR (400 MHz, DMSO-*d*_6_) *δ*: 10.53 (s, 1H, NH), 8.50 (s, 1H, NH), 7.40–7.36 (m, 2H, ArH), 7.32–7.28 (m, 3H, ArH), 7.20 (s, 2H, NH_2_), 4.57 (d, *J* = 3.2 Hz, 1H, CH), 4.14–4.09 (m, 3H, CH), 3.94 (d, *J* = 6.0 Hz, 2H, CH), 1.20 (t, *J* = 7.2 Hz, 3H, CH_3_); ^13^C NMR (100 MHz, DMSO-*d*_6_) *δ*: 171.9, 170.0, 162.1, 153.5, 141.8, 129.1, 127.9, 127.5, 118.6, 70.7, 61.0, 55.6, 52.0, 41.7, 14.5; IR (KBr) *ν*: 3408, 3369, 3317, 3193, 3097, 2973, 2204, 1753, 1701, 1654, 1591, 1548, 1415, 1356, 1321, 1248, 1210, 1135, 1019, 977, 856 cm^−1^; MS (*m*/*z*): HRMS (ESI) calcd for C_17_H_18_N_4_O_4_S ([M + Na]^+^): 397.1049, found: 397.0938.

#### 4.1.2 Ethyl ((5-amino-4-cyano-3-(4-methoxyphenyl)-2,3-dihydrothiophene-2-carbonyl)carbamoyl)alaninate (2a)

White solid, 68%, mp 218–220 °C; ^1^H NMR (400 MHz, DMSO-*d*_6_) *δ*: 10.58 (s, 1H, NH), 8.52 (s, 1H, NH), 7.23–7.18 (m, 4H, ArH, NH_2_), 6.93 (d, *J* = 8.8 Hz, 2H, ArH), 4.51–4.50 (m, 1H, CH), 4.34–4.27 (m, 1H, CH), 4.12 (m, 2H, CH), 4.05 (s, 2H, CH), 3.75 (s, 3H, OCH_3_), 1.36 (d, *J* = 7.2 Hz, 3H, CH_3_), 1.20 (t, *J* = 7.2 Hz, 3H, CH_3_); ^13^C NMR (100 MHz, DMSO-*d*_6_) *δ*: 172.5, 172.3, 161.7, 159.1, 152.8, 133.6, 128.6, 118.6, 114.5, 71.1, 61.2, 55.8, 55.5, 51.4, 48.6, 18.0, 14.4; IR (KBr) *ν*: 3449, 3328, 3227, 3155, 2968, 2848, 2180, 1740, 1695, 1624, 1582, 1547, 1506, 1348, 1309, 1252, 1193, 1030, 829 cm^−1^; MS (*m*/*z*): HRMS (ESI) calcd for C_19_H_22_N_4_O_5_S ([M + Na]^+^): 441.1311, found: 441.1206.

### 4.2. General procedure for the preparation of the thiophene derivatives 3a–3j

A mixture of aromatic aldehyde (2.0 mmol) and malononitrile (2.0 mmol) and triethylamine (3.0 mmol) in ethanol (20.0 mL) was stirred at room temperature for one hour. Then, 1,3-thiazolidinedione (2.0 mmol) and α-amino acid ethyl ester hydrochloride (2.0 mmol) was added. The resulting mixture was stirred at about 40–50 °C for six hours. Then, DDQ (2.2 mmol) was added. The mixture was stirred at 50–60 °C for additional for four hours. After removing the solvent by rotatory evaporation at reduced pressure, the residue was subjected to column chromatography with light petroleum and ethyl acetate (v/v = 2 : 1) as eluent to give pure product for analysis.

#### 4.2.1 Ethyl ((5-amino-4-cyano-3-(4-methoxyphenyl)thiophene-2-carbonyl)carbamoyl)phenylalaninate (3a)

Yellow solid, 62%, mp 177–179 °C; ^1^H NMR (400 MHz, DMSO-*d*_6_) *δ*: 8.49 (d, *J* = 7.6 Hz, 1H, NH), 8.10 (d, *J* = 8.0 Hz, 3H, NH, NH_2_), 7.35 (d, *J* = 8.4 Hz, 2H, ArH), 7.31–7.21 (m, 3H, ArH), 7.13 (d, *J* = 8.8 Hz, 2H, ArH), 7.08 (d, *J* = 8.8 Hz, 2H, ArH), 4.51–4.46 (m, 1H, CH), 4.06 (q, *J* = 7.2 Hz, 2H, CH), 3.82 (s, 3H, OCH_3_), 3.07–2.97 (m, 2H, CH), 1.13 (t, *J* = 7.2 Hz, 3H, CH_3_); ^13^C NMR (100 MHz, DMSO-*d*_6_) *δ*: 171.2, 167.6, 161.9, 160.6, 152.0, 146.0, 136.7, 130.8, 129.5, 128.7, 127.2, 124.4, 115.3, 114.9, 111.9, 89.5, 61.3, 55.7, 54.2, 37.4, 14.3; IR (KBr) *ν*: 3438, 3373, 3281, 3168, 2967, 2211, 1730, 1680, 1648, 1516, 1467, 1412, 1316, 1256, 1181, 1078, 1024, 876, 840 cm^−1^; MS (*m*/*z*): HRMS (ESI) calcd for C_25_H_24_N_4_O_5_S ([M + Na]^+^): 515.1467, found: 515.1357.

## Conflicts of interest

There are no conflicts to declare.

## Supplementary Material

RA-008-C8RA03605E-s001

RA-008-C8RA03605E-s002
