# Supplementary material for: Tandem four-component reaction for efficient synthesis of dihydrothiophene with substituted amino acid ethyl esters
Source: RSC Adv. 2018 Jun 20;8(40):22498–505. doi: 10.1039/c8ra03605e (PMC9081350; doi:10.1039/c8ra03605e)
Supplement: RA-008-C8RA03605E-s001 [file RA-008-C8RA03605E-s001.pdf]

Electronic Supplementary Material (ESI) for RSC Advances.
This journal is © The Royal Society of Chemistry 2018

Tandem four-component reaction for efficient synthesis of dihydrothiophene with substituted amino acid ethyl esters

Jing Sun, Yu Zhang, Chao-Guo Yan*

Supporting Information

Figures of single crystal structures Fig. s1-s6 **2-4**

^{1}H NMR, ^{13}C NMR and HRMS spectra of all compounds **5-68**

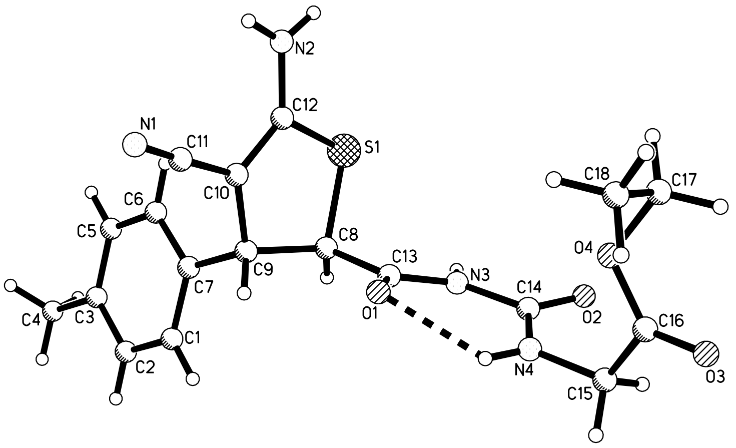

Fig. s1 Crystal structure of the compound **1d**

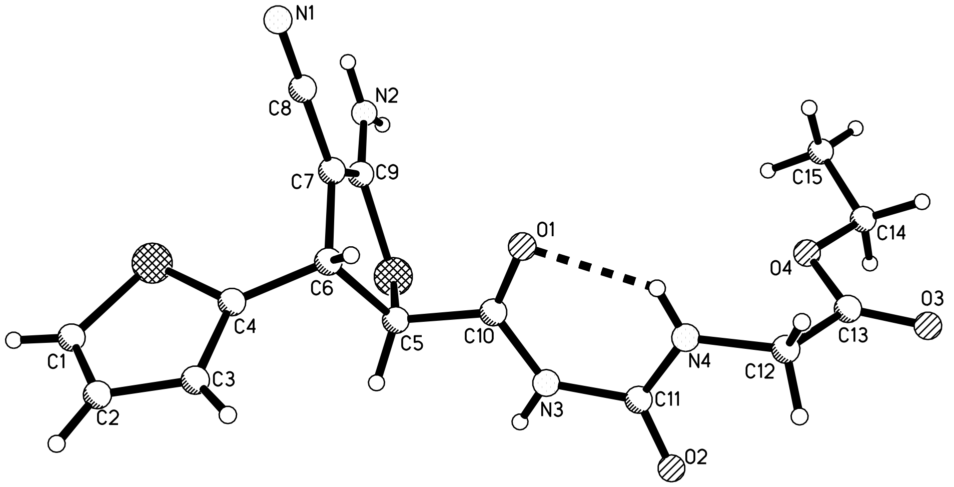

Fig. s2 Crystal structure of the compound **1h**

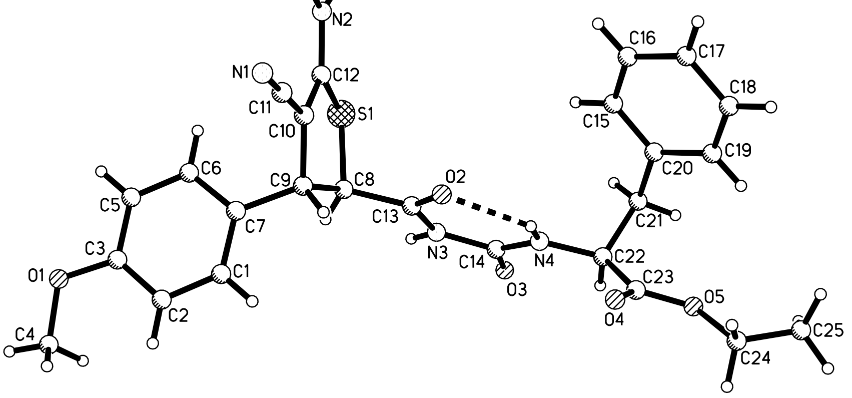

Fig. s3 Crystal structure of the compound **2g**

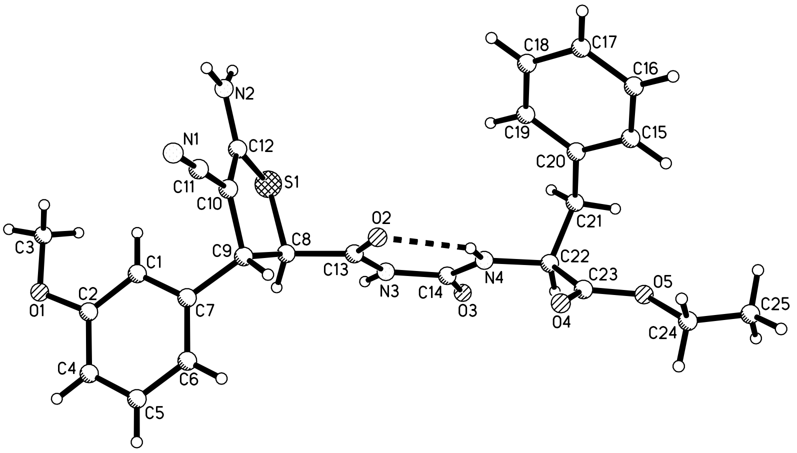

Fig. s4 Crystal structure of the compound **2h**

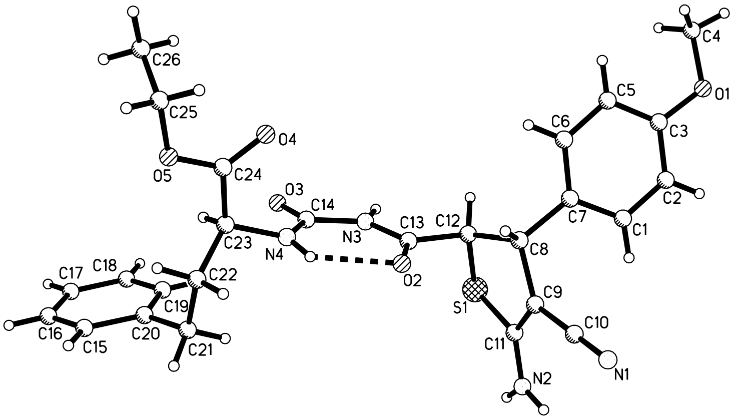

Fig. s5 Crystal structure of the compound **2m**

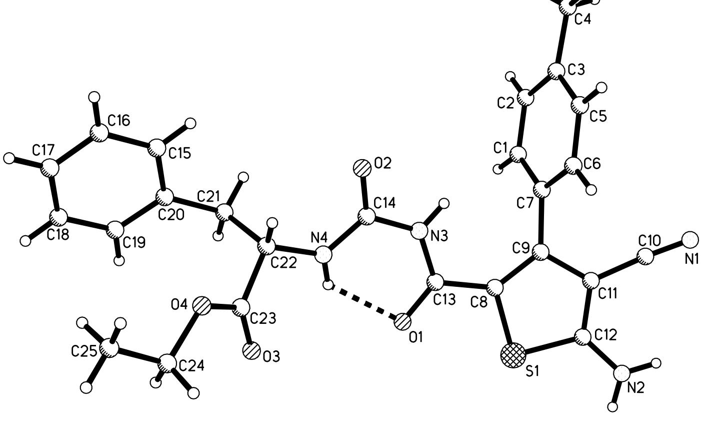

Fig. s6 Crystal structure of the compound **3d**

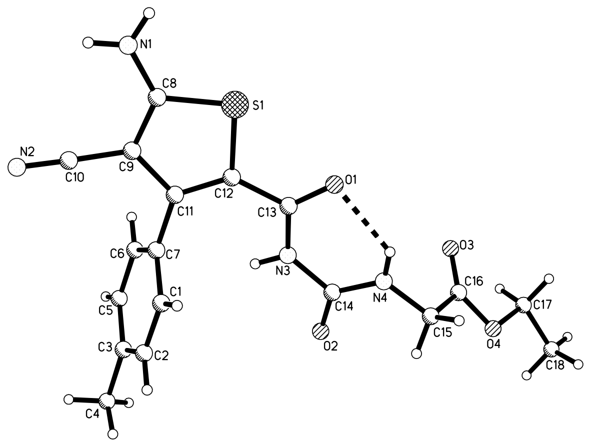

Fig. s7 Crystal structure of the compound **3g**

Ethyl ((5-amino-4-cyano-3-phenyl-2,3-dihydrothiophene-2-carbonyl)carbamoyl)glycinate (1a): yellow solid, 49%, m.p. 188-190℃; ^{1}H NMR (400 MHz, DMSO-d_6) δ: 10.53 (s, 1H, NH), 8.50 (s, 1H, NH), 7.40-7.36 (m, 2H, ArH), 7.32-7.28 (m, 3H, ArH), 7.20 (s, 2H, NH_2), 4.57 (d, J = 3.2 Hz, 1H, CH), 4.14-4.09 (m, 3H, CH), 3.94 (d, J = 6.0 Hz, 2H, CH), 1.20 (t, J = 7.2 Hz, 3H, CH_3); ^{13}C NMR (100 MHz, DMSO-d6) δ: 171.9, 170.0, 162.1, 153.5, 141.8, 129.1, 127.9, 127.5, 118.6, 70.7, 61.0, 55.6, 52.0, 41.7, 14.5; IR (KBr) υ: 3408, 3369, 3317, 3193, 3097, 2973, 2204, 1753, 1701, 1654, 1591, 1548, 1415, 1356, 1321, 1248, 1210, 1135, 1019, 977, 856 cm^{-1}; MS (m/z): HRMS (ESI) Calcd. for $C_{17}H_{18}N_4O_4S$ ($[M+Na]^+$): 397.1049, found: 397.0938.

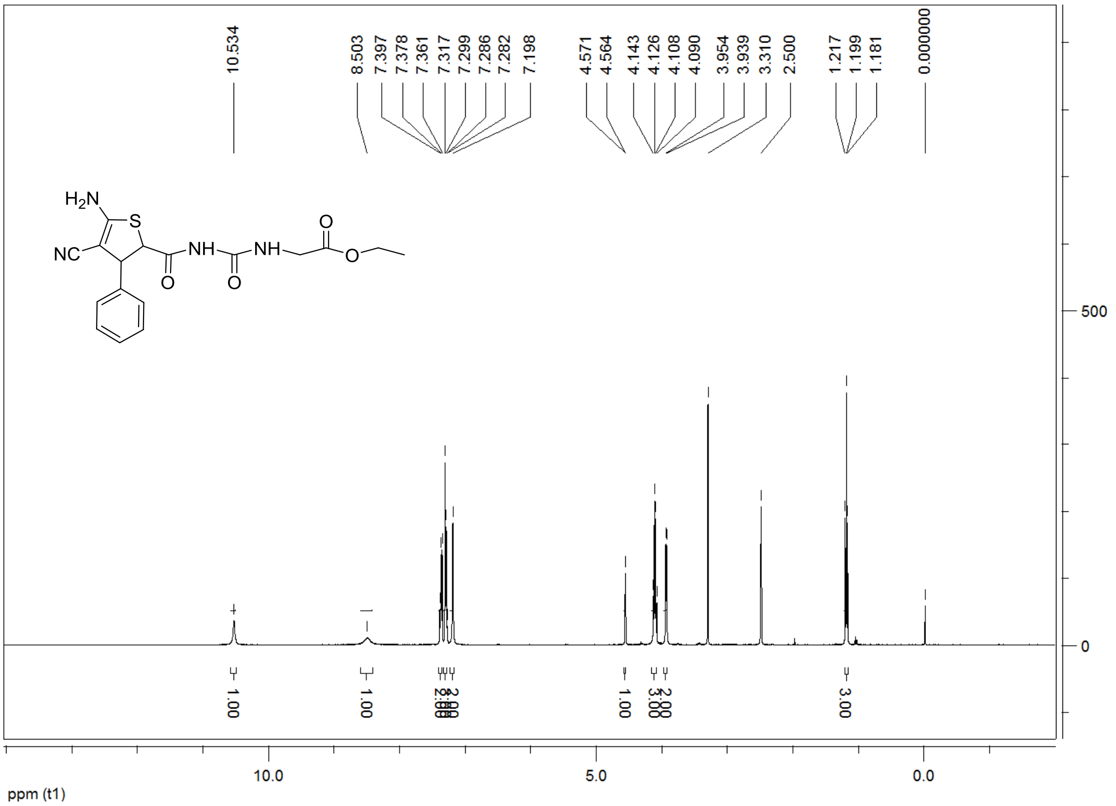

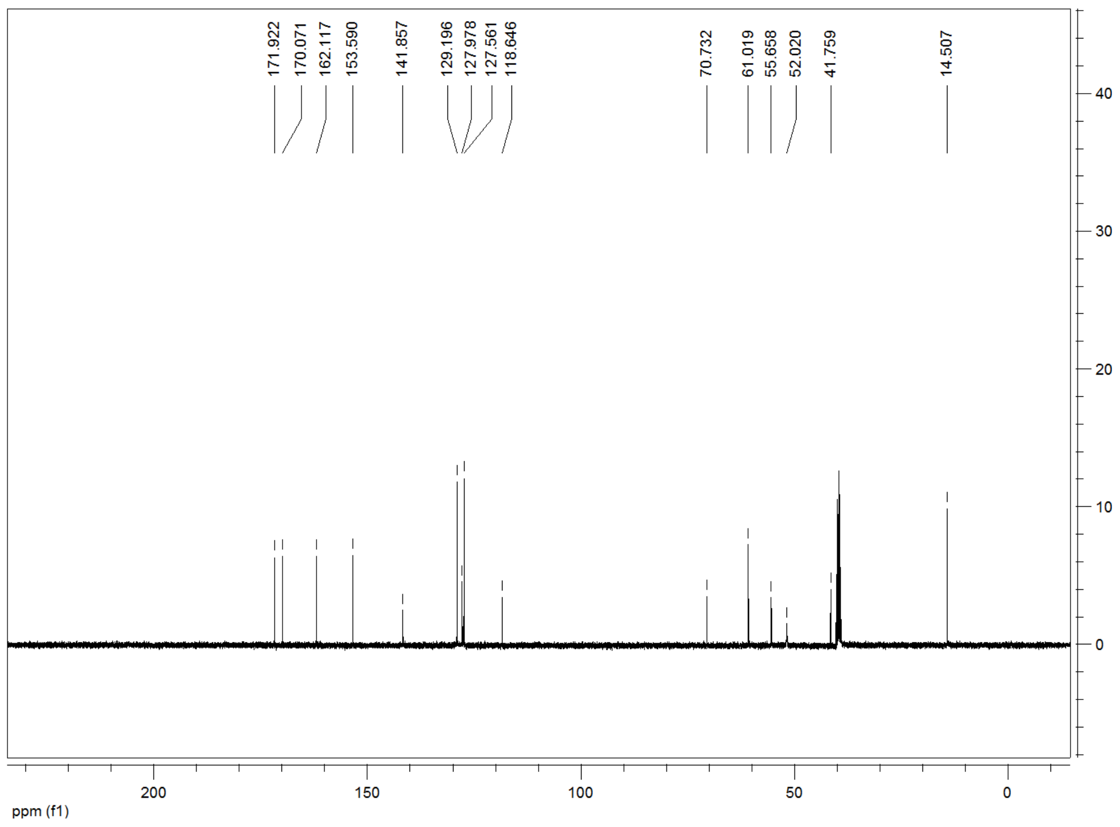
171.922
170.071
162.117
153.590
141.857
129.196
127.978
127.561
118.646
70.732
61.019
55.658
52.020
41.759
14.507
40
30
20
10
0
200
150
100
50
0
ppm (f1)

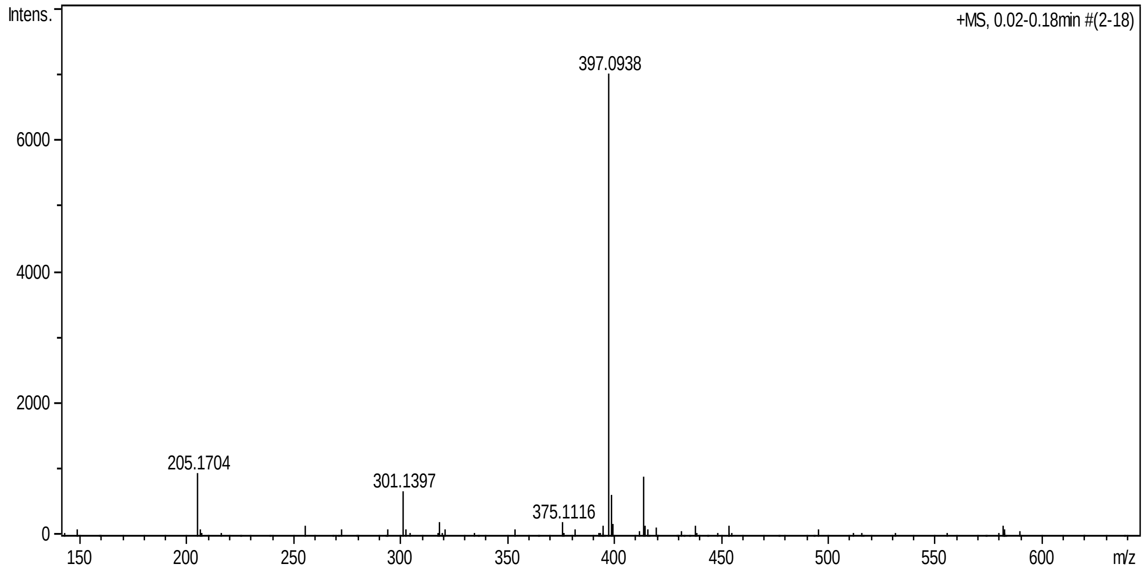
Intens.
+MS, 0.02-0.18min #(2-18)
397.0938
6000
4000
2000
0
205.1704
301.1397
375.1116
150
200
250
300
350
400
450
500
550
600
m/z

Ethyl ((5-amino-4-cyano-3-(4-methoxyphenyl)-2,3-dihydrothiophene-2-carbonyl)carbamoyl)glycinate (1b): yellow solid, 65%, m.p. 202-204℃; ^{1}H NMR (400 MHz, DMSO-*d*$_6$) δ: 10.54 (s, 1H, NH), 8.51 (s, 1H, NH), 7.21 (d, *J* = 8.4 Hz, 2H, ArH), 7.17 (s, 2H, NH_2), 6.93 (d, *J* = 8.4 Hz, 2H, ArH), 4.52 (d, *J* = 3.2 Hz, 1H, CH), 4.14-4.08 (m, 3H, CH), 3.94 (d, *J* = 5.6 Hz, 2H, CH), 3.75 (s, 3H, OCH_3), 1.20 (t, *J* = 7.2 Hz, 3H, CH_3); ^{13}C NMR (100 MHz, DMSO-d6) δ: 171.9, 170.0, 161.7, 159.1, 153.5, 133.6, 128.6, 118.6, 114.5, 71.0, 60.9, 55.8, 55.5, 51.5, 41.7, 14.5; IR (KBr) υ: 3442, 3321, 3227, 3160, 2970, 2837, 2176, 1749, 1697, 1629, 1580, 1506, 1352, 1310, 1257, 1187, 1028, 982, 888, 828 cm^{-1}; MS (*m*/*z*): HRMS (ESI) Calcd. for $C_{18}H_{20}N_4O_5S$ ($[M+H]^+$): 405.1154, found: 405.1236.

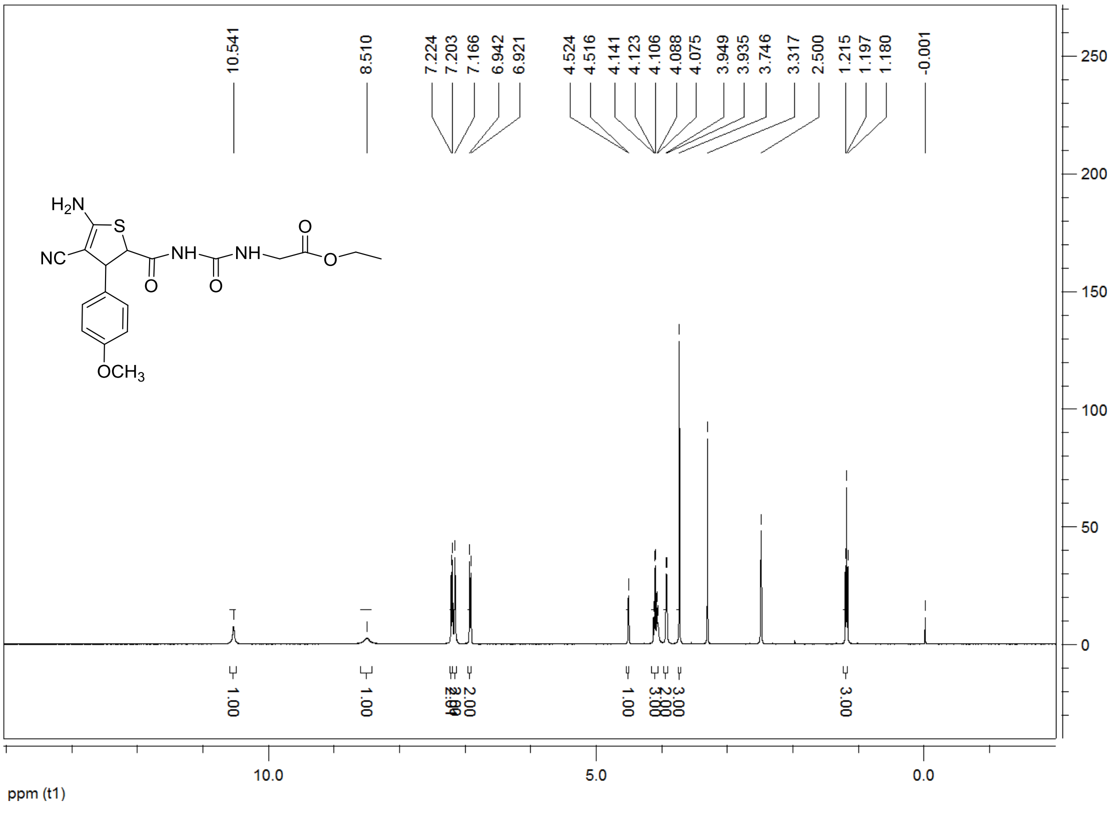

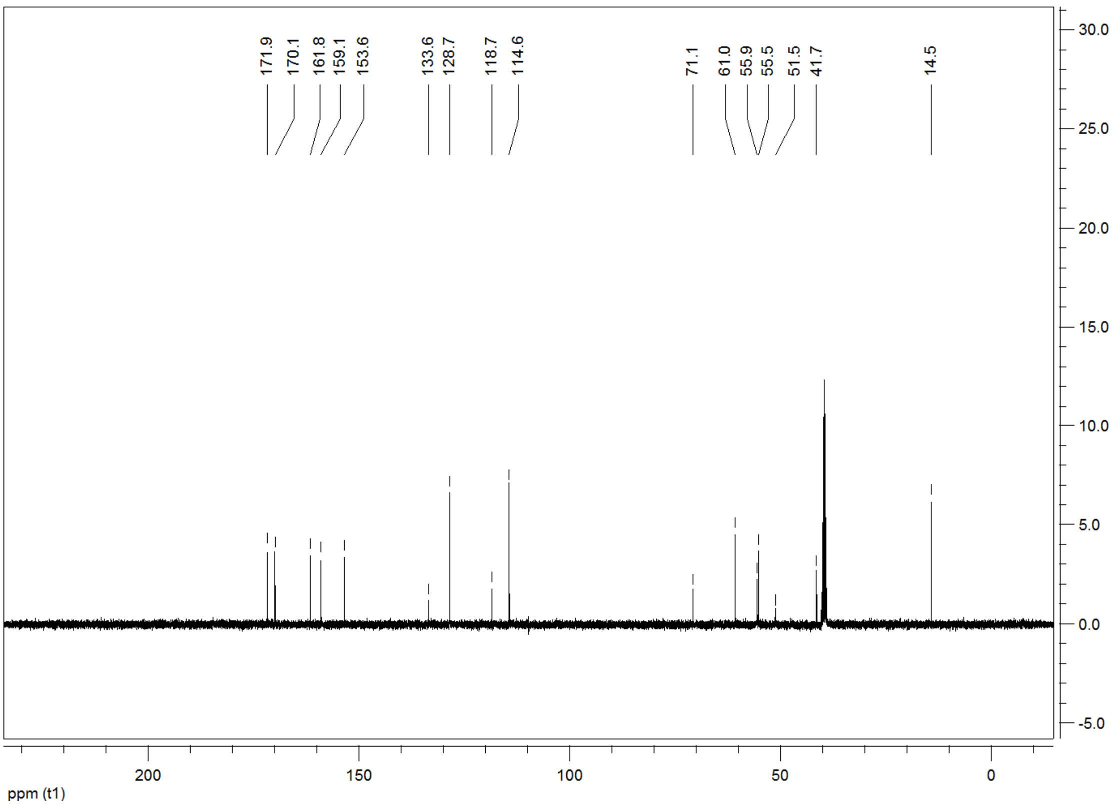
171.9
170.1
161.8
159.1
153.6
133.6
128.7
118.7
114.6
71.1
61.0
55.9
55.5
51.5
41.7
14.5
30.0
25.0
20.0
15.0
10.0
5.0
0.0
-5.0
200
150
100
50
0
ppm (t1)

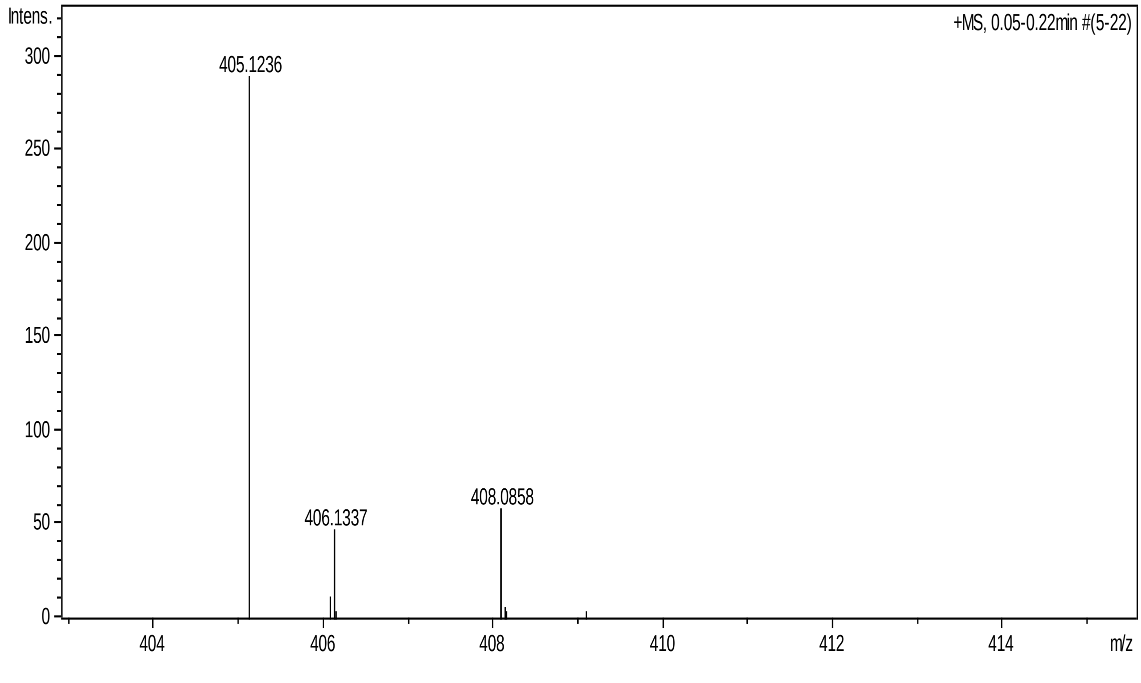
Intens.
+MS, 0.05-0.22min #(5-22)
405.1236
406.1337
408.0858
300
250
200
150
100
50
0
404
406
408
410
412
414
m/z

Ethyl ((5-amino-4-cyano-3-(2-methoxyphenyl)-2,3-dihydrothiophene-2-carbonyl)carbamoyl)glycinate (1c): yellow solid, 63%, m.p. 220-222℃; ^{1}H NMR (400 MHz, DMSO-*d*$_6$) δ: 10.48 (s, 1H, NH), 8.53 (s, 1H, NH), 7.30-7.26 (m, 1H, ArH), 7.20 (s, 2H, NH_2), 7.12-7.11 (m, 1H, ArH), 7.03-7.01 (m, 1H, ArH), 6.98-6.95 (m, 1H, ArH), 4.81 (s, 1H, CH), 4.09 (q, *J* = 7.2 Hz, 2H, CH_2), 3.93 (d, *J* = 5.6 Hz, 2H, CH), 3.88 (s, 1H, CH), 3.76 (s, 3H, OCH_3), 1.18 (t, *J* = 7.2 Hz, 3H, CH_3); ^{13}C NMR (100 MHz, DMSO-d6) δ: 172.5, 170.1, 162.7, 156.7, 153.6, 129.1, 129.0, 127.1, 120.8, 118.6, 111.5, 68.1, 61.0, 56.0, 54.5, 45.8, 41.7, 14.5; IR (KBr) υ: 3407, 3337, 3238, 3124, 2977, 2175, 1739, 1698, 1650, 1577, 1549, 1494, 1389, 1352, 1283, 1239, 1138, 1105, 1022, 807 cm^{-1}; MS (*m/z*): HRMS (ESI) Calcd. for $C_{18}H_{20}N_4O_5S$ ([M+Na]$^+$): 427.1154, found: 427.1050.

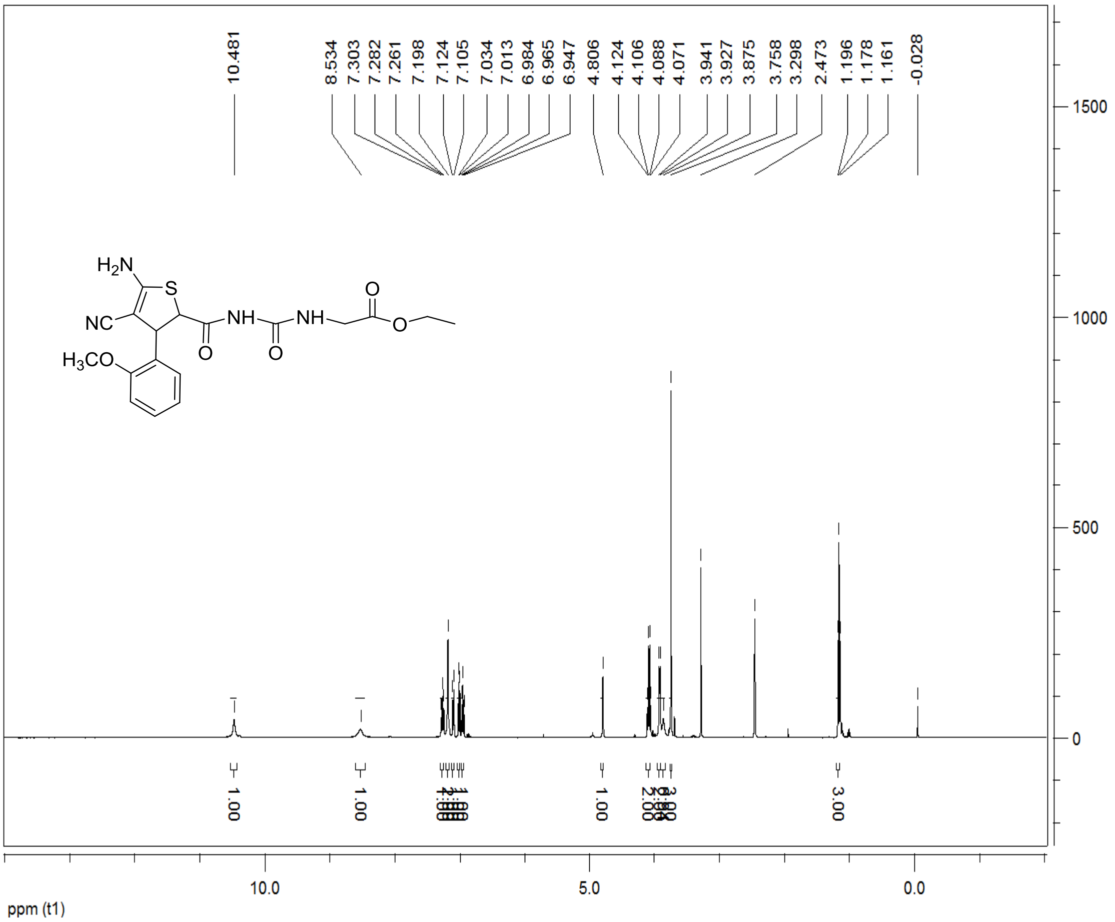

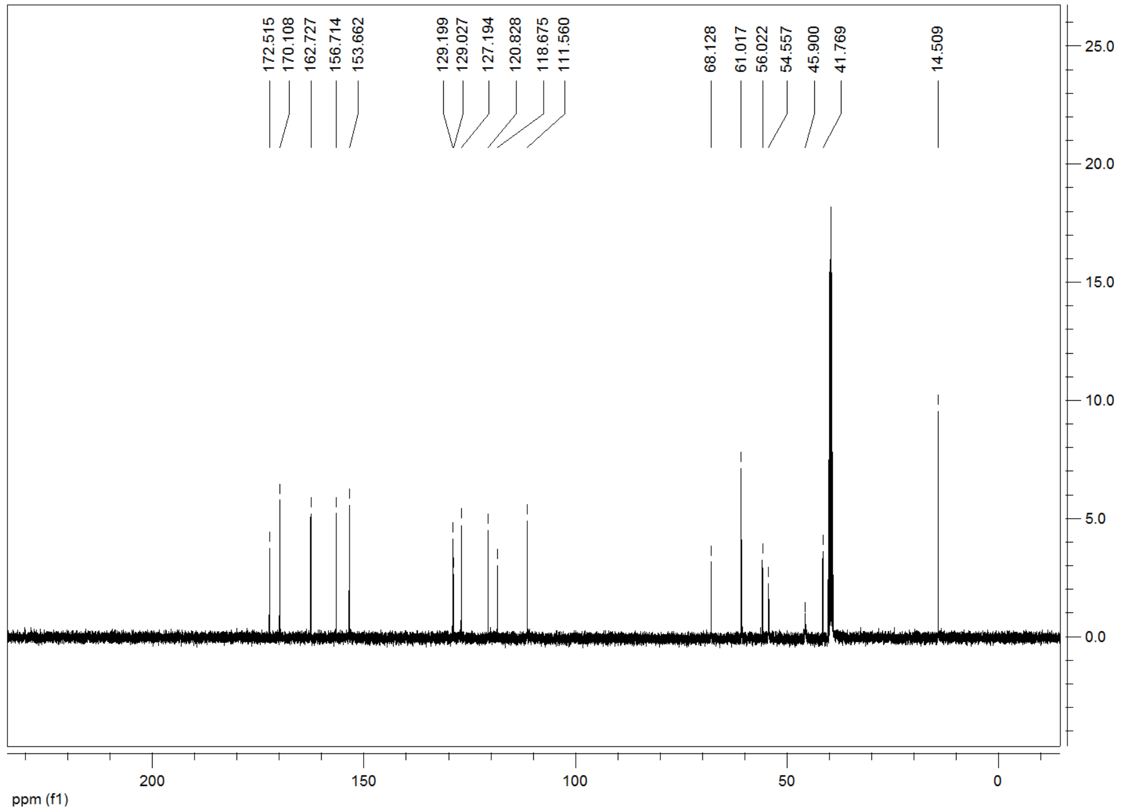
172.515
170.108
162.727
156.714
153.662
129.199
129.027
127.194
120.828
118.675
111.560
68.128
61.017
56.022
54.557
45.900
41.769
14.509
25.0
20.0
15.0
10.0
5.0
0.0
200
150
100
50
0
ppm (f1)

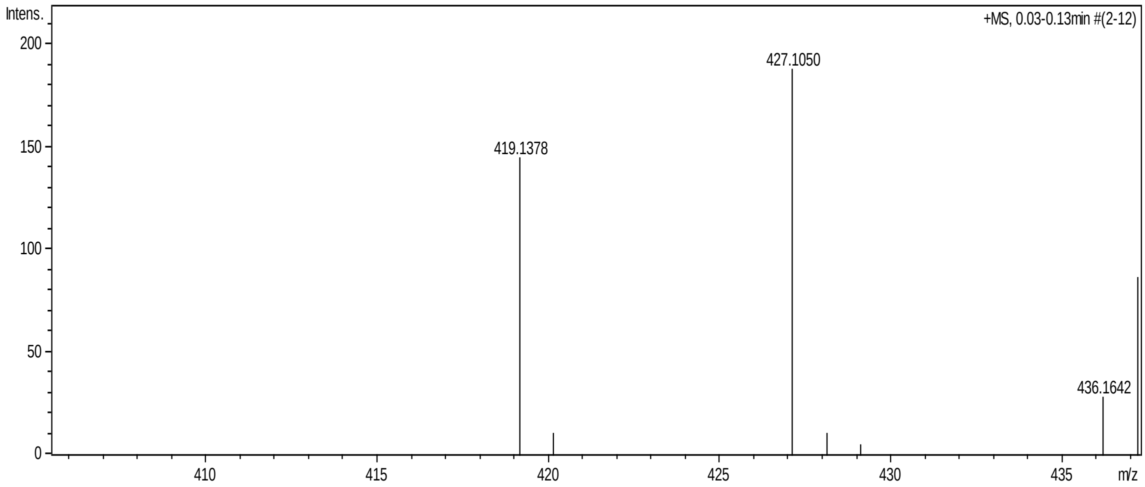
Intens.
+MS, 0.03-0.13min #(2-12)
427.1050
419.1378
436.1642
200
150
100
50
0
410
415
420
425
430
435
m/z

Ethyl ((5-amino-4-cyano-3-(p-tolyl)-2,3-dihydrothiophene-2-carbonyl)carbamoyl)glycinate (1d): yellow solid, 60%, m.p. 204-206℃; ^{1}H NMR (400 MHz, DMSO-d_6) δ: 10.54 (s, 1H, NH), 8.50 (s, 1H, NH), 7.20-7.18 (m, 4H, ArH), 7.15 (s, 2H, NH_2), 4.52 (d, J = 3.2 Hz, 1H, CH), 4.14-4.09 (m, 3H, CH), 3.94 (d, J = 6.0 Hz, 2H, CH), 2.29 (s, 3H, CH_3), 1.20 (t, J = 7.2 Hz, 3H, CH_3); ^{13}C NMR (100 MHz, DMSO-d6) δ: 171.9, 170.0, 161.9, 153.5, 138.7, 137.1, 129.7, 127.4, 118.6, 70.8, 61.0, 55.7, 51.8, 41.7, 21.0, 14.5; IR (KBr) υ: 3436, 3326, 3226, 2976, 2177, 1747, 1697, 1630, 1579, 1498, 1350, 1256, 1191, 1025, 981, 885 cm^{-1}; MS (m/z): HRMS (ESI) Calcd. for $C_{18}H_{20}N_4O_4S$ ($[M+Na]^+$): 411.1205, found: 411.1096.

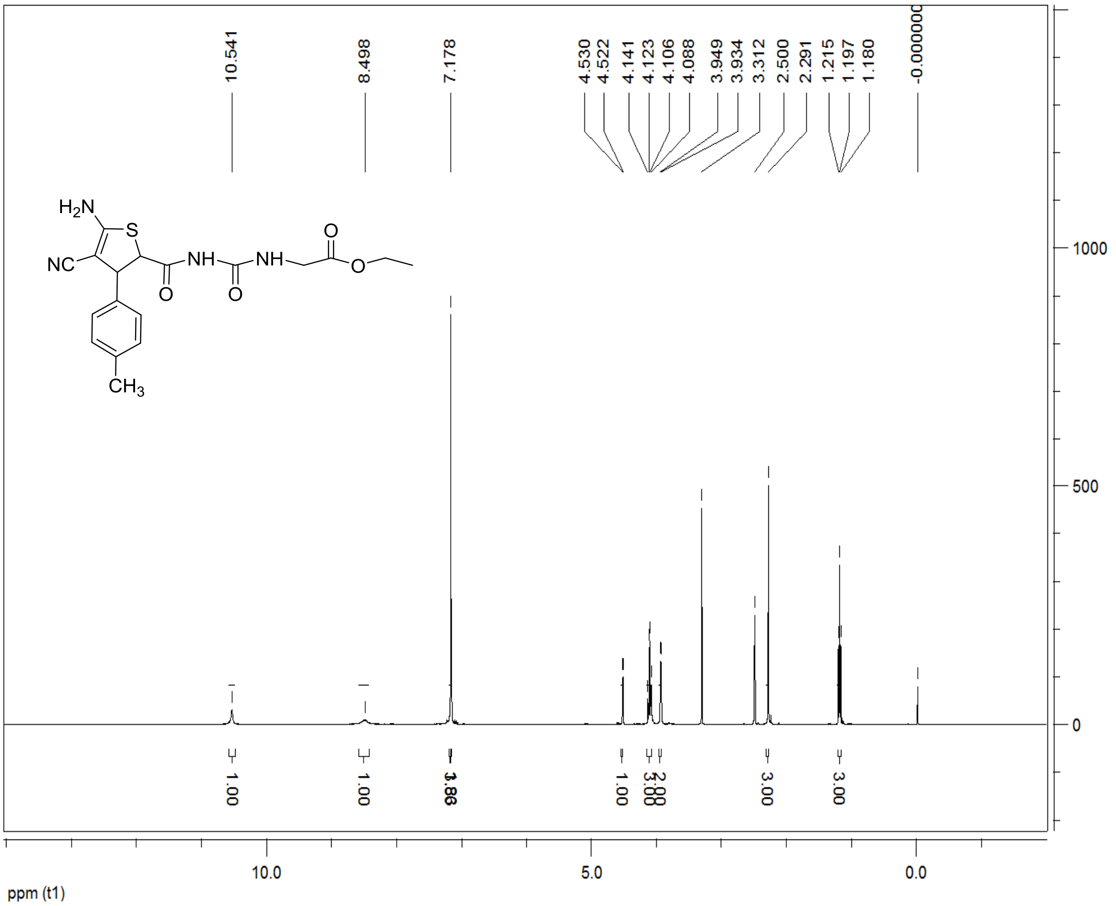

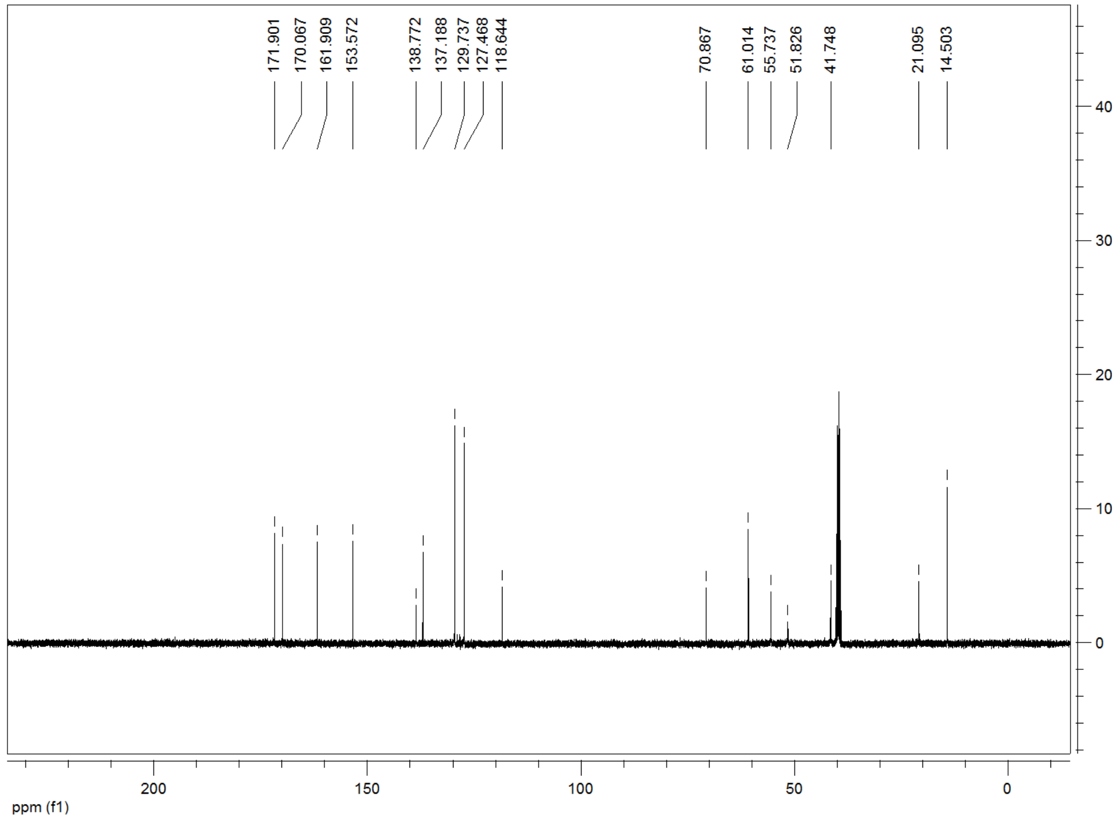
171.901
170.067
161.909
153.572
138.772
137.188
129.737
127.468
118.644
70.867
61.014
55.737
51.826
41.748
21.095
14.503
ppm (f1)
200
150
100
50
0
40
30
20
10
0

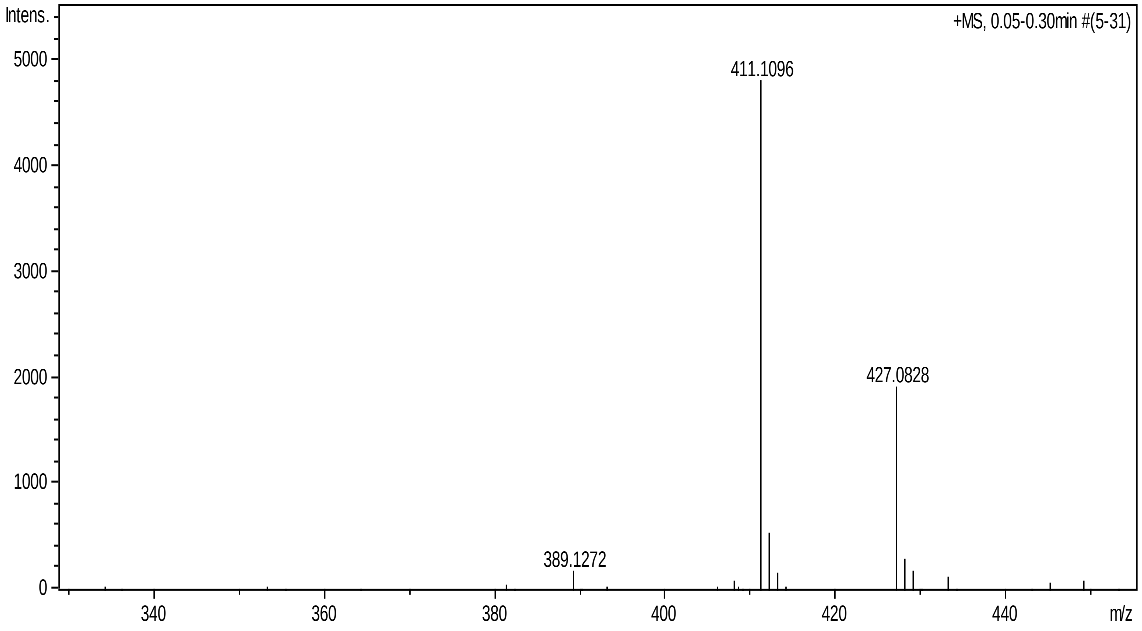
Intens.
+MS, 0.05-0.30min #(5-31)
411.1096
427.0828
389.1272
5000
4000
3000
2000
1000
0
340
360
380
400
420
440
m/z

Ethyl ((5-amino-4-cyano-3-(m-tolyl)-2,3-dihydrothiophene-2-carbonyl)carbamoyl)glycinate (1e): yellow solid, 54%, m.p. 208-210℃; ^{1}H NMR (400 MHz, DMSO-d_6) δ: 10.53 (s, 1H, NH), 8.50 (s, 1H, NH), 7.28-7.24 (m, 1H, ArH), 7.18 (s, 2H, NH_2), 7.12-7.08 (m, 3H, ArH), 4.53 (s,1H, CH), 4.14-4.09 (m, 3H, CH), 3.94 (d, J = 6.0 Hz, 2H, CH), 2.31 (s, 3H, CH_3), 1.20 (t, J = 7.2 Hz, 3H, CH_3); ^{13}C NMR (100 MHz, DMSO-d6) δ: 171.9, 170.0, 162.0, 153.5, 141.8, 138.3, 129.1, 128.6, 128.1, 124.6, 118.6, 70.7, 61.0, 55.6, 52.0, 41.7, 21.5, 14.5; IR (KBr) υ: 3424, 3332, 3226, 3157, 2978, 2177, 1739, 1682, 1629, 1586, 1499, 1413, 1342, 1264, 1195, 1133, 1030, 985 892, 817 cm^{-1}; MS (m/z): HRMS (ESI) Calcd. for $C_{18}H_{20}N_4O_4S$ ([M+Na]$^+$): 411.1205, found: 411.1097.

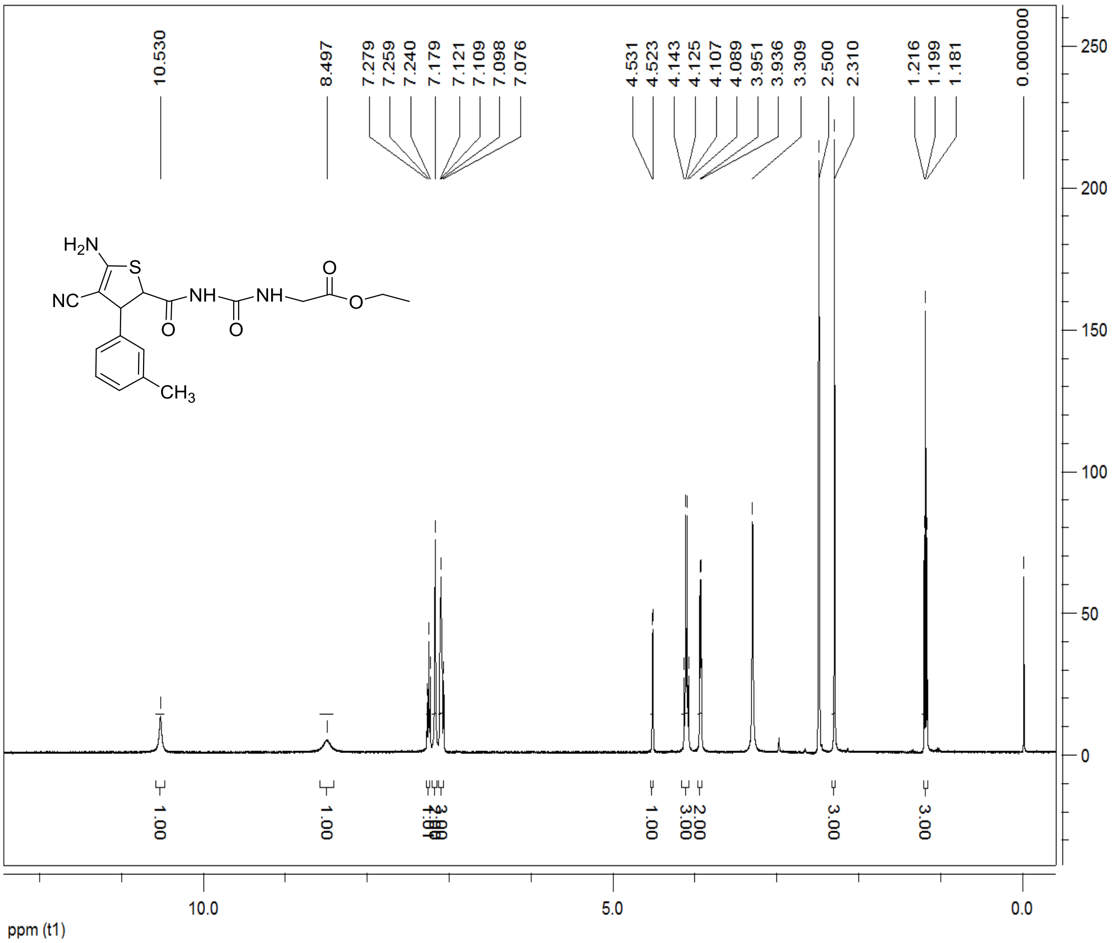

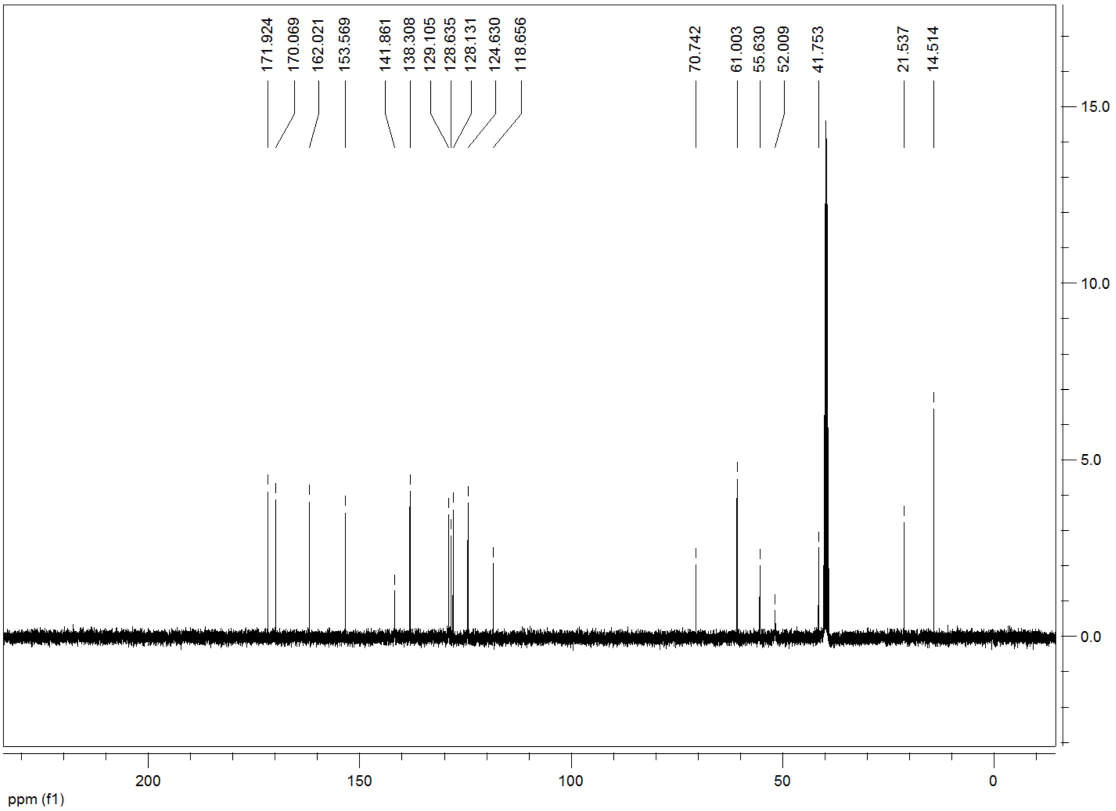
171.924
170.069
162.021
153.569
141.861
138.308
129.105
128.635
128.131
124.630
118.656
70.742
61.003
55.630
52.009
41.753
21.537
14.514
15.0
10.0
5.0
0.0
200
150
100
50
0
ppm (f1)

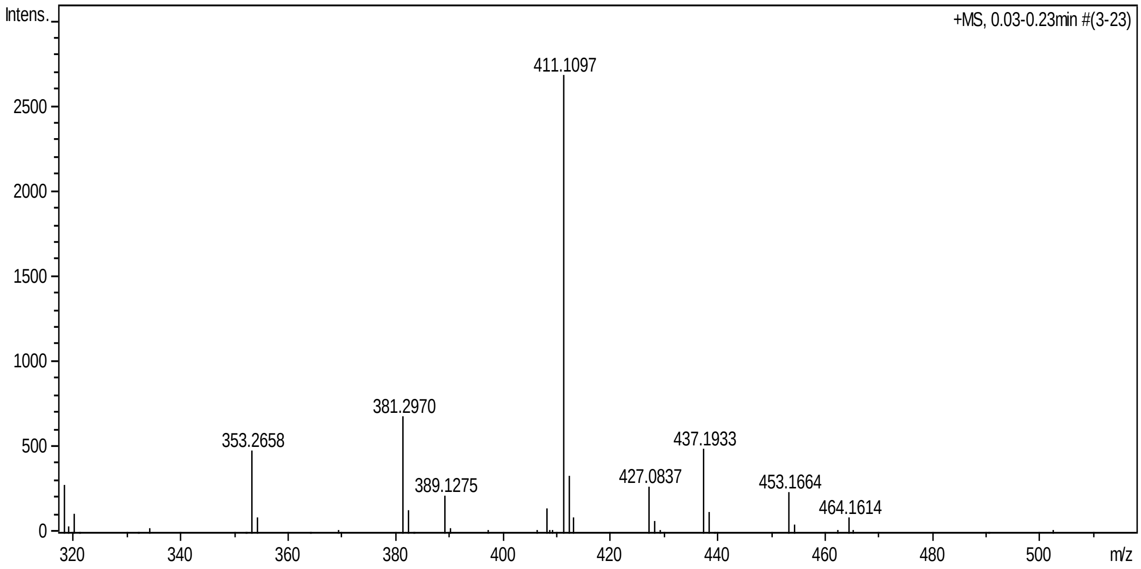
Intens.
+MS, 0.03-0.23min #(3-23)
411.1097
381.2970
353.2658
389.1275
437.1933
427.0837
453.1664
464.1614
2500
2000
1500
1000
500
0
320
340
360
380
400
420
440
460
480
500
m/z

Ethyl ((5-amino-3-(4-chlorophenyl)-4-cyano-2,3-dihydrothiophene-2-carbonyl)carbamoyl)glycinate (1f): yellow solid, 66%, m.p. 195-197℃; ^{1}H NMR (400 MHz, DMSO-d_6) δ: 10.55 (s, 1H, NH), 8.51 (s, 1H, NH), 7.46-7.44 (m, 2H, ArH), 7.35-7.33 (m, 2H, ArH), 7.26 (s, 2H, NH_2), 4.57 (d, J = 2.8 Hz, 1H, CH), 4.14-4.08 (m, 3H, CH), 3.94 (d, J = 5.6 Hz, 2H, CH), 1.20 (t, J = 7.2 Hz, 3H, CH_3); ^{13}C NMR (100 MHz, DMSO-d6) δ: 171.7, 170.0, 162.3, 153.5, 140.8, 132.5, 129.5, 129.1, 118.5, 70.3, 60.9, 55.5, 51.2, 41.7, 14.5; IR (KBr) υ: 3453, 3342, 3236, 3113, 2969, 2190, 1740, 1706, 1613, 1582, 1529, 1408, 1339, 1266, 1203, 1095, 1023, 982, 826 cm^{-1}; MS (m/z): HRMS (ESI) Calcd. for $C_{17}H_{17}ClN_4O_4S$ ($[M+H]^+$): 409.0659, found: 409.0722.

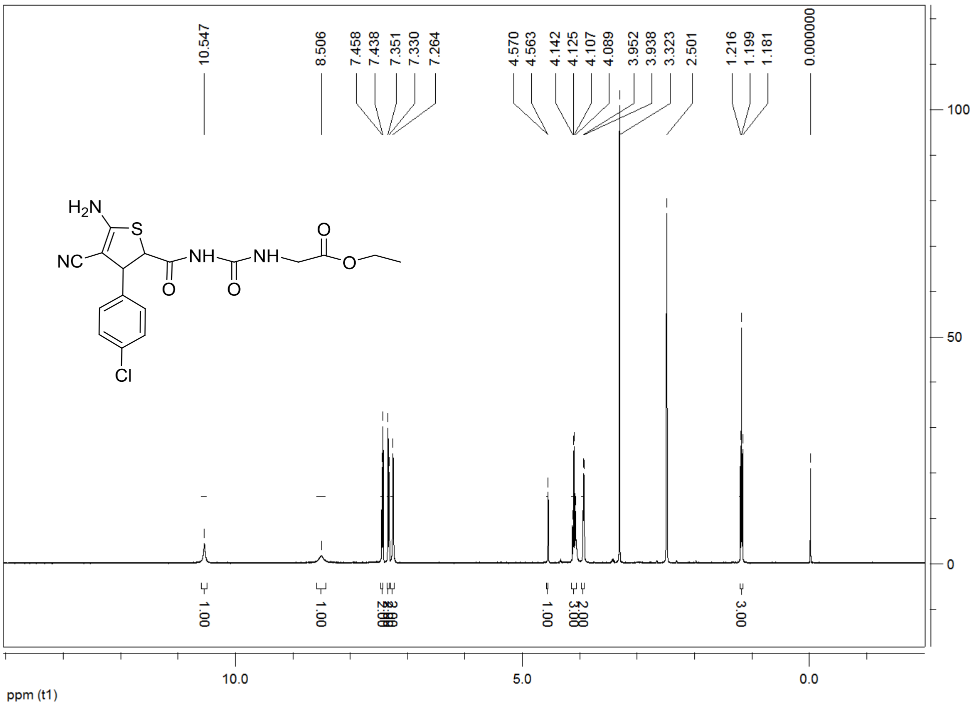

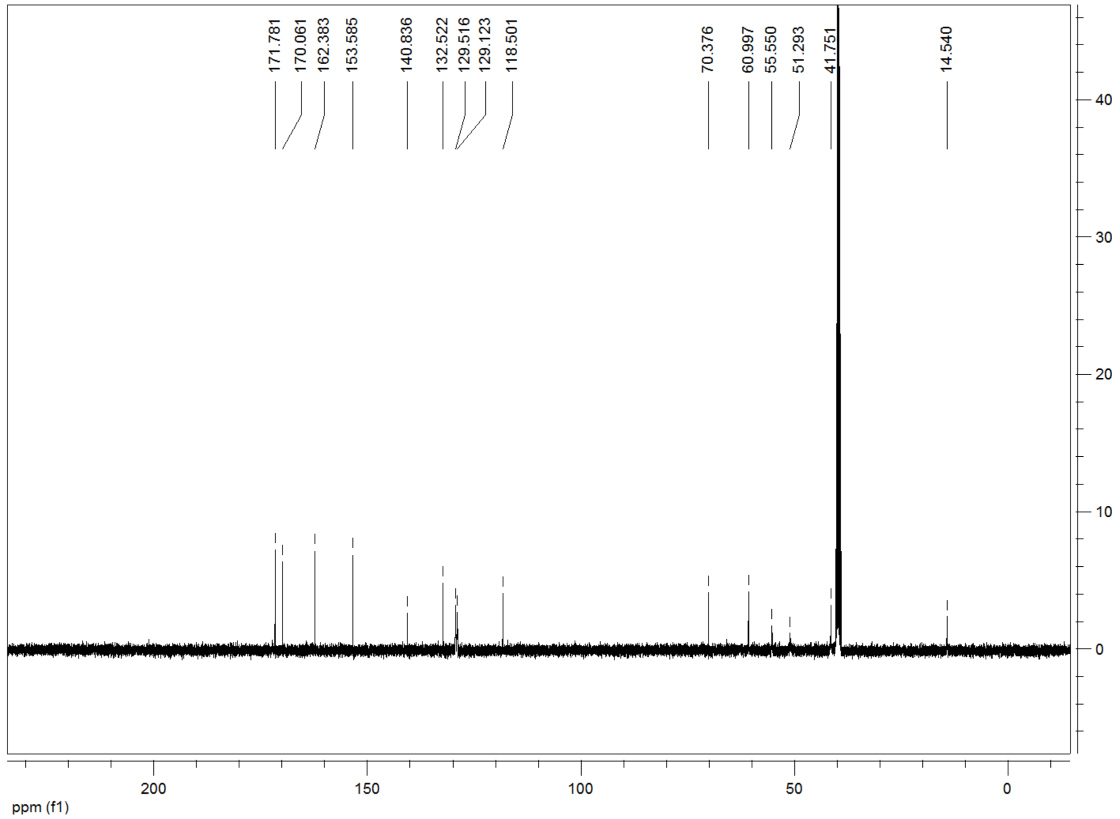

171.781
170.061
162.383
153.585
140.836
132.522
129.516
129.123
118.501
70.376
60.997
55.550
51.293
41.751
14.540
40
30
20
10
0
200
150
100
50
0
ppm (f1)

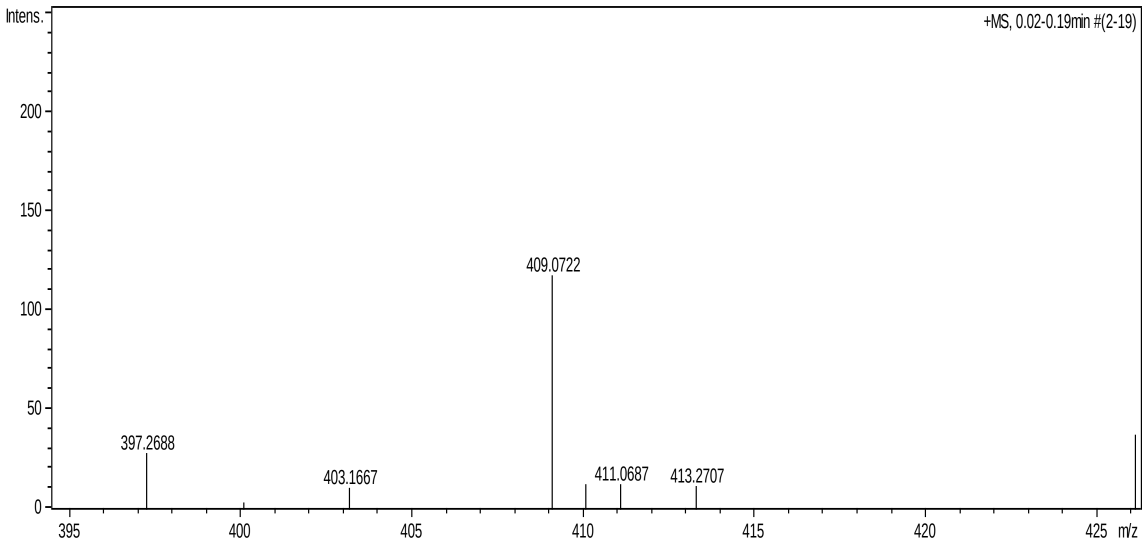

Intens.
+MS, 0.02-0.19min #(2-19)
200
150
100
50
0
409.0722
397.2688
403.1667
411.0687
413.2707
395
400
405
410
415
420
425
m/z

Ethyl ((5-amino-4-cyano-3-(3-nitrophenyl)-2,3-dihydrothiophene-2-carbonyl)carbamoyl)glycinate (1g): yellow solid, 58%, m.p. 188-190℃; ^{1}H NMR (400 MHz, DMSO-d_6) δ: 10.54 (s, 1H, NH), 8.54 (s, 1H, NH), 8.22-8.17 (m, 2H, ArH), 7.82-7.80 (m, 1H, ArH), 7.72-7.68 (m, 1H, ArH), 7.38 (s, 2H, NH_2), 4.74 (s, 1H, CH), 4.13-4.11 (m, 3H, CH), 3.96-3.95 (m, 2H, CH), 1.20 (t, J = 6.8 Hz, 3H, CH_3); ^{13}C NMR (100 MHz, DMSO-d6) δ: 172.7, 171.4, 171.3, 161.9, 161.8, 153.3, 153.2, 138.9, 137.2, 137.1, 129.6, 127.4, 118.6, 71.0, 61.2, 57.0, 55.7, 51.6, 37.3, 25.1, 21.0, 15.9, 14.5, 11.7; IR (KBr) υ: 3343, 3207, 2967, 2168, 1709, 1615, 1580, 1534, 1511, 1346, 1240, 1181, 1028, 831 cm^{-1}; MS (m/z): HRMS (ESI) Calcd. for $C_{17}H_{17}N_5NaO_6S$ ($[M+H]^+$): 442.0792, found: 442.0798.

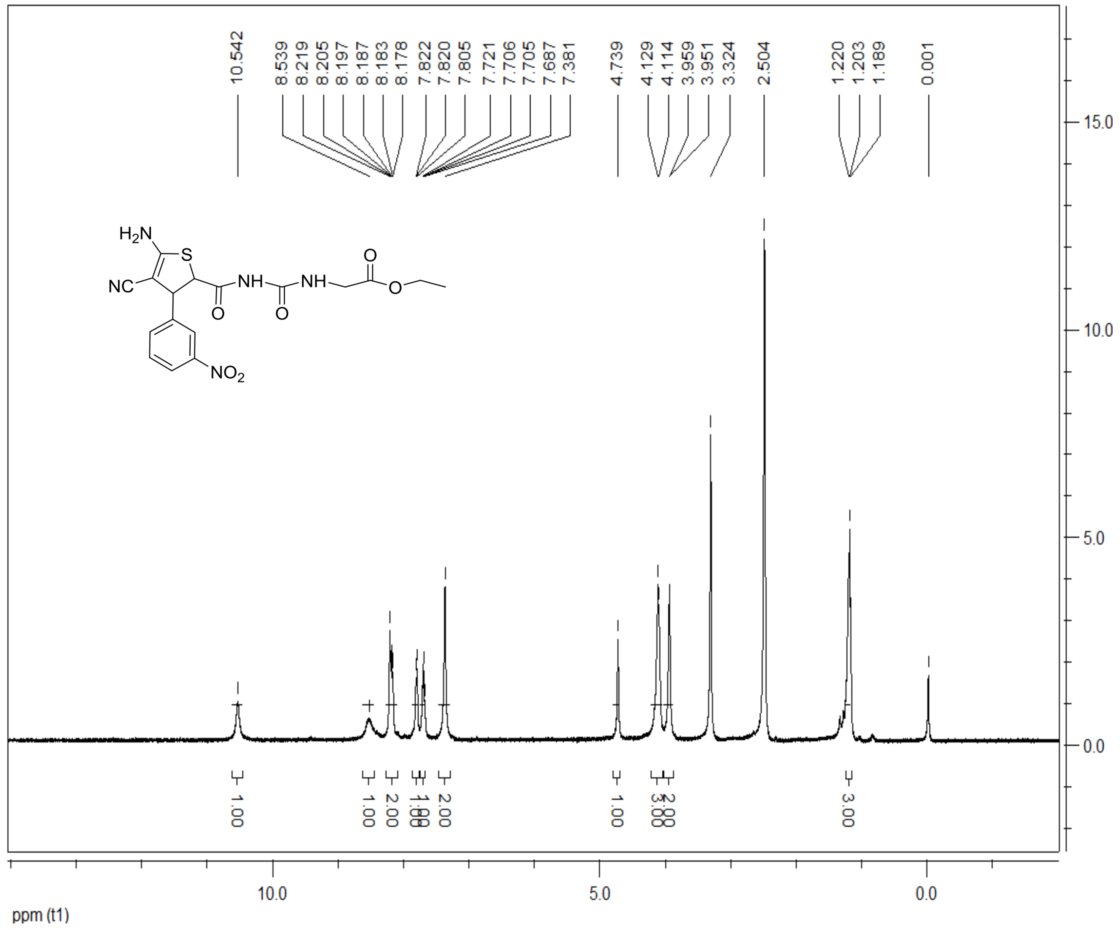

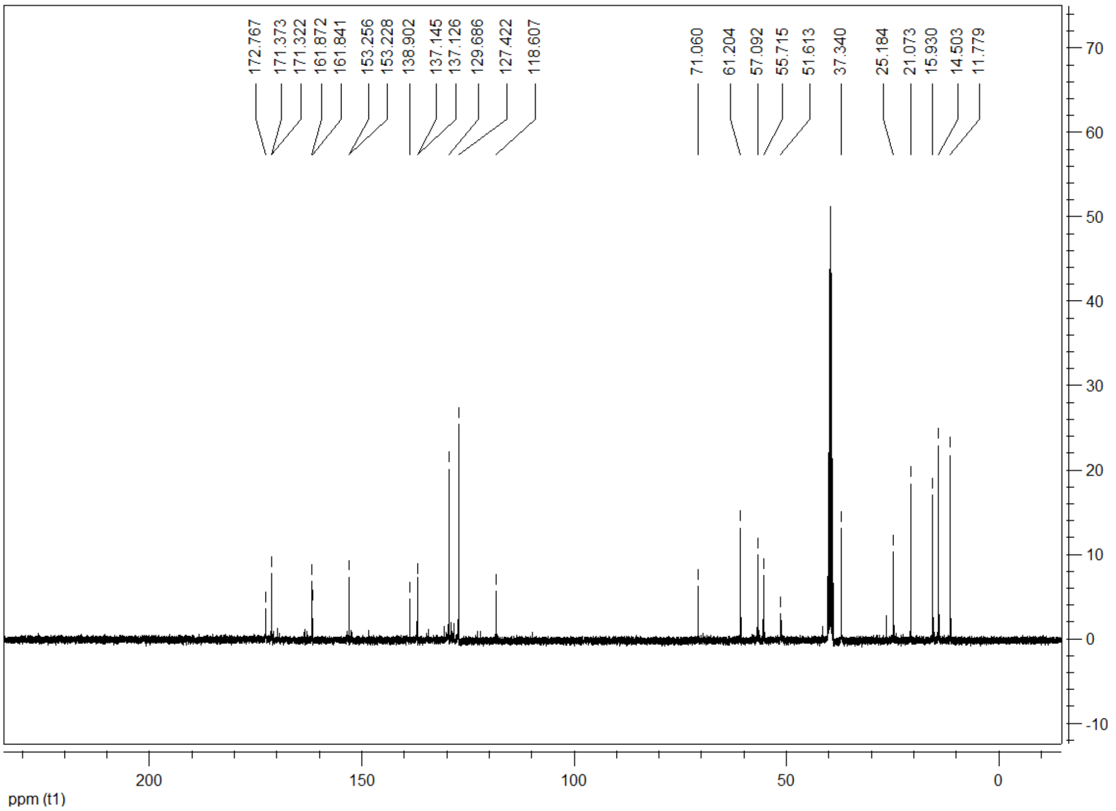
172.767
171.373
171.322
161.872
161.841
153.256
153.228
138.902
137.145
137.126
129.686
127.422
118.607
71.060
61.204
57.092
55.715
51.613
37.340
25.184
21.073
15.930
14.503
11.779
ppm (t1)

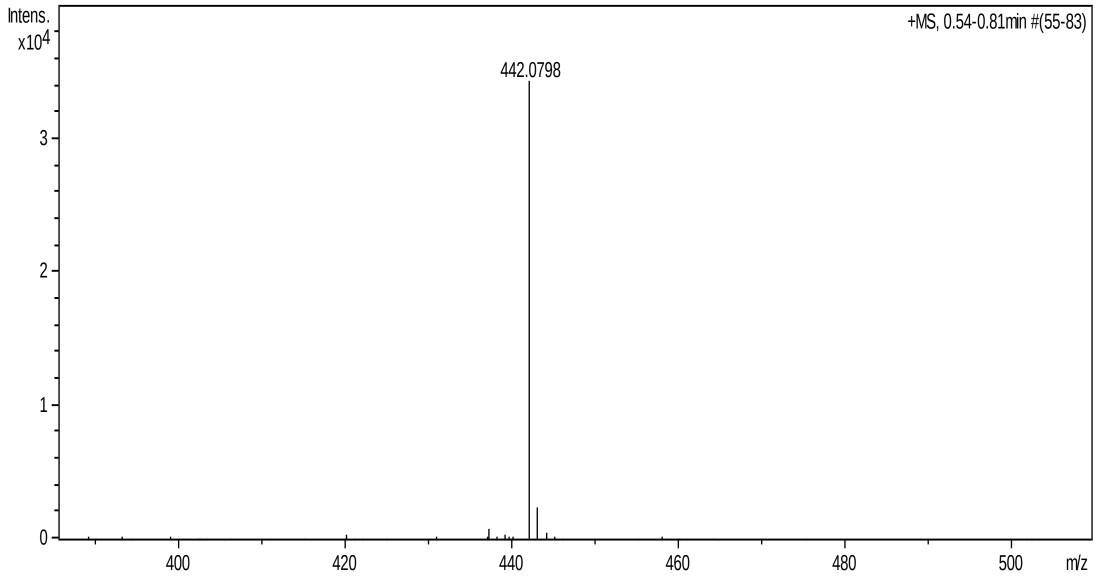
Intens.
x10^4
+MS, 0.54-0.81min #(55-83)
442.0798
m/z

Ethyl ((5'-amino-4'-cyano-2',3'-dihydro-[2,3'-bithiophene]-2'-carbonyl)carbamoyl)glycinate (1h): yellow solid, 60%, m.p. 179-181℃; ^{1}H NMR (400 MHz, DMSO-d_6) δ: 10.60 (s, 1H, NH), 8.52 (s, 1H, NH), 7.44-7.42 (m, 1H, ArH), 7.27 (s, 2H, NH_2), 7.07-7.04 (m, 1H, ArH), 7.01-6.99 (m, 1H, ArH), 4.86 (d, J = 2.4 Hz, 1H, CH), 4.15-4.09 (m, 3H, CH), 3.94 (d, J = 5.6 Hz, 2H, CH), 1.20 (t, J = 7.2 Hz, 3H, CH_3); ^{13}C NMR (100 MHz, DMSO-d6) δ: 171.6, 170.0, 161.9, 153.5, 146.1, 127.5, 125.5, 125.4, 118.4, 71.5, 61.0, 55.9, 47.3, 41.7, 14.5; IR (KBr) υ: 3469, 3308, 3393, 3117, 3073, 2997, 2253, 1704, 1691, 1554, 1536, 1318, 1235, 1210, 1118, 1077, 947, 856 cm^{-1}; MS (m/z): HRMS (ESI) Calcd. for $C_{15}H_{14}NaN_4O_4S_2$ ($[M+Na]^+$): 403.0505, found: 403.0498.

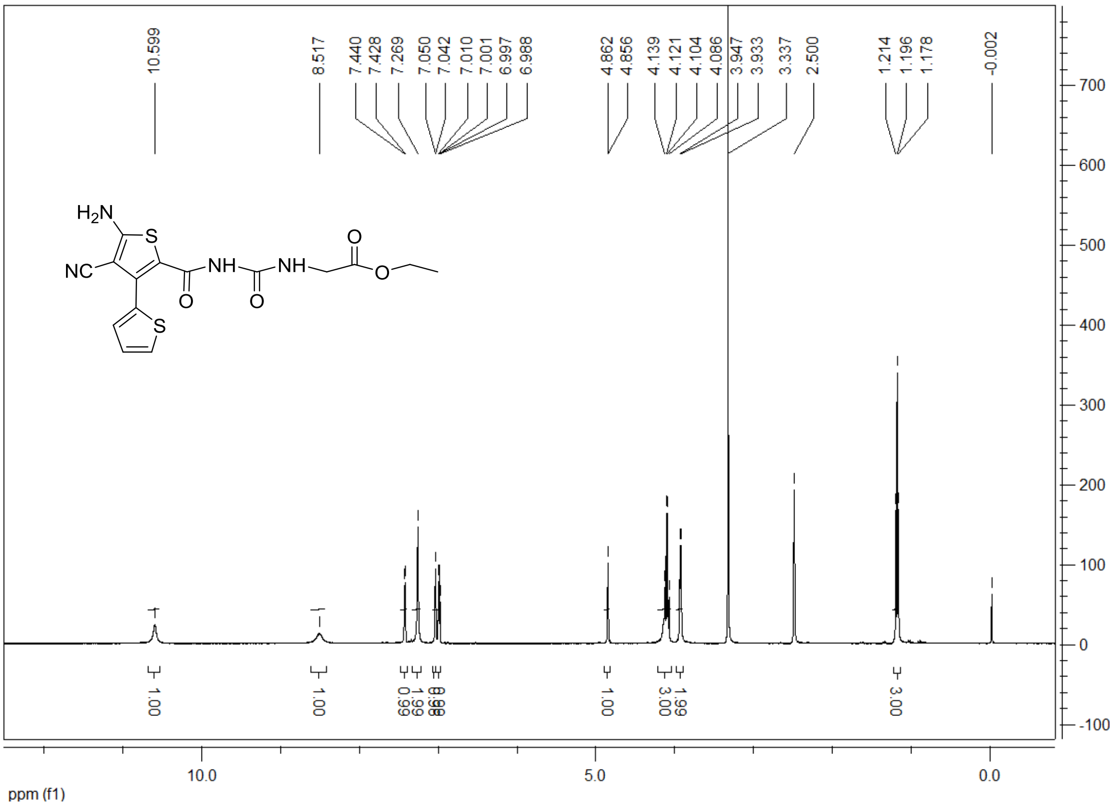

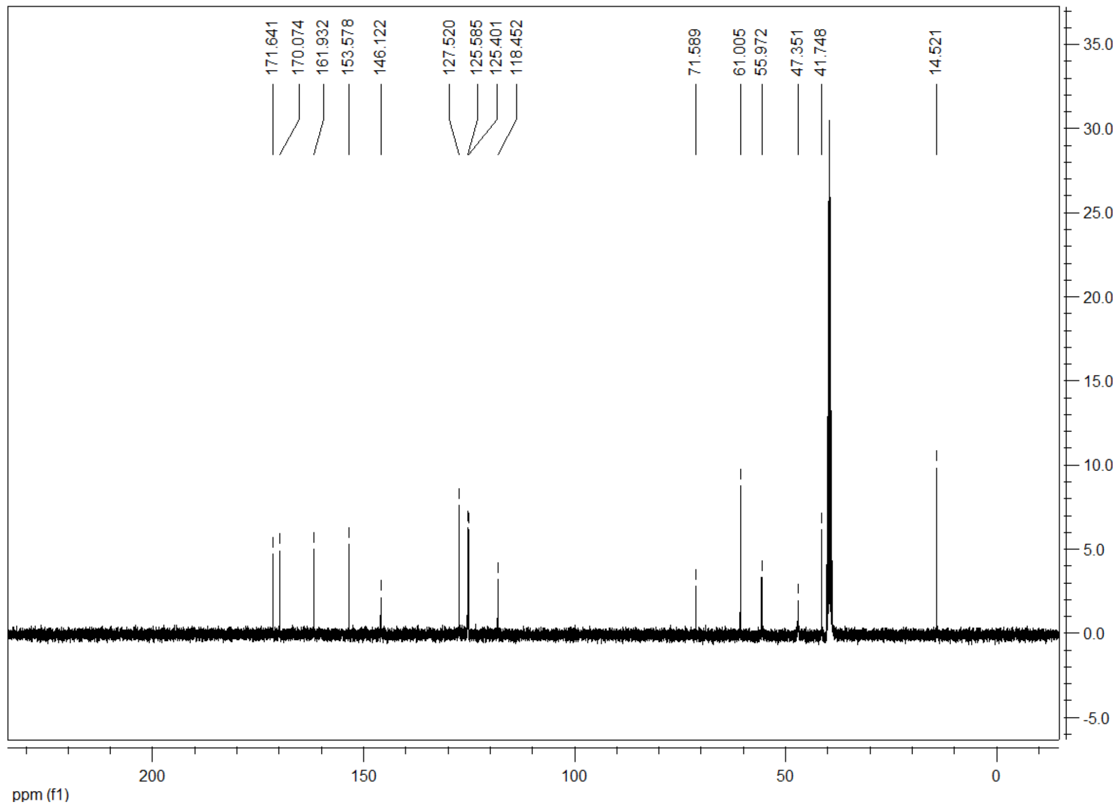
171.641
170.074
161.932
153.578
146.122
127.520
125.585
125.401
118.452
71.589
61.005
55.972
47.351
41.748
14.521
35.0
30.0
25.0
20.0
15.0
10.0
5.0
0.0
-5.0
200
150
100
50
0
ppm (f1)

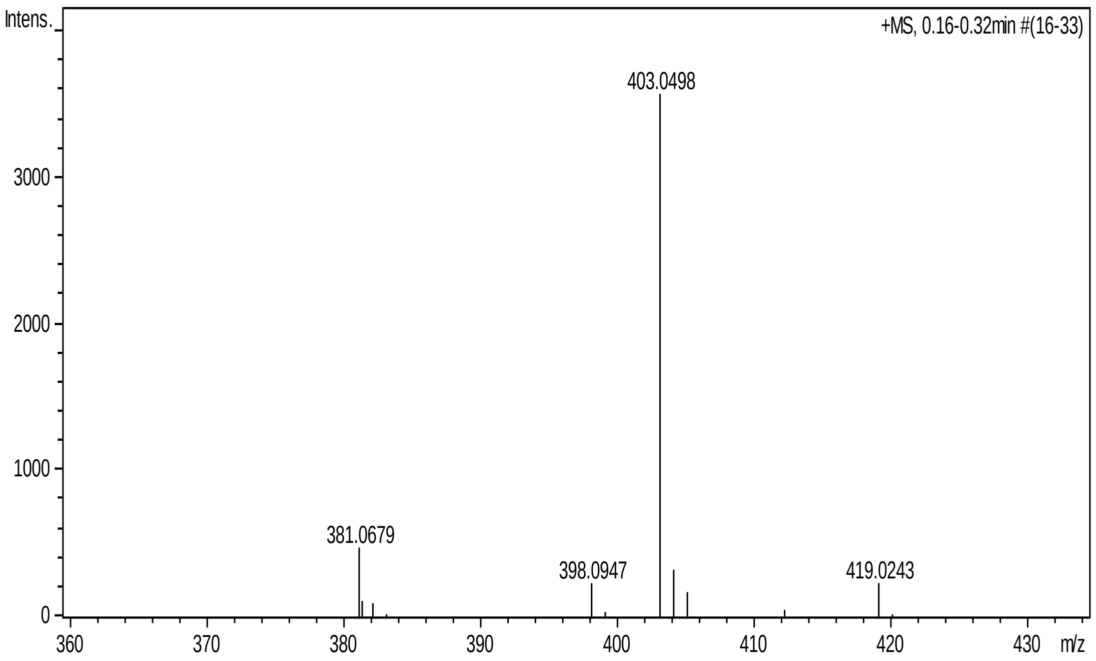
Intens.
+MS, 0.16-0.32min #(16-33)
403.0498
381.0679
398.0947
419.0243
3000
2000
1000
0
360
370
380
390
400
410
420
430
m/z

Ethyl ((5-amino-4-cyano-3-(4-methoxyphenyl)-2,3-dihydrothiophene-2-carbonyl)carbamoyl)alaninate (2a): white solid, 68%, m.p. 218-220℃; ^{1}H NMR (400 MHz, DMSO-*d*$_6$) δ: 10.58 (s, 1H, NH), 8.52 (s, 1H, NH), 7.23-7.18 (m, 4H, ArH, NH_2), 6.93 (d, *J* = 8.8 Hz, 2H, ArH), 4.51-4.50 (m, 1H, CH), 4.34-4.27 (m, 1H, CH), 4.12 (m, 2H, CH), 4.05 (s, 2H, CH), 3.75 (s, 3H, OCH_3), 1.36 (d, *J* = 7.2 Hz, 3H, CH_3), 1.20 (t, *J* = 7.2 Hz, 3H, CH_3); ^{13}C NMR (100 MHz, DMSO-d6) δ: 172.5, 172.3, 161.7, 159.1, 152.8, 133.6, 128.6, 118.6, 114.5, 71.1, 61.2, 55.8, 55.5, 51.4, 48.6, 18.0, 14.4; IR (KBr) υ: 3449, 3328, 3227, 3155, 2968, 2848, 2180, 1740, 1695, 1624, 1582, 1547, 1506, 1348, 1309, 1252, 1193, 1030, 829 cm^{-1}; MS (*m*/*z*): HRMS (ESI) Calcd. for $C_{19}H_{22}N_4O_5S$ ($[M+Na]^+$): 441.1311, found: 441.1206.

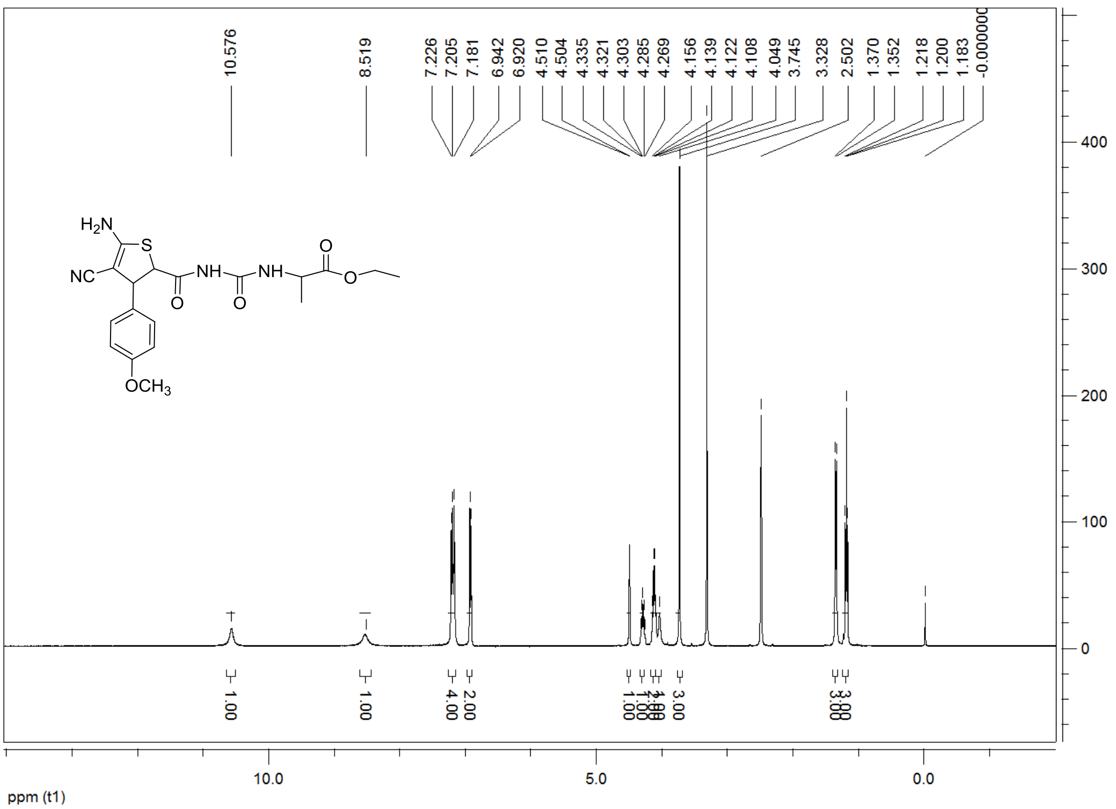

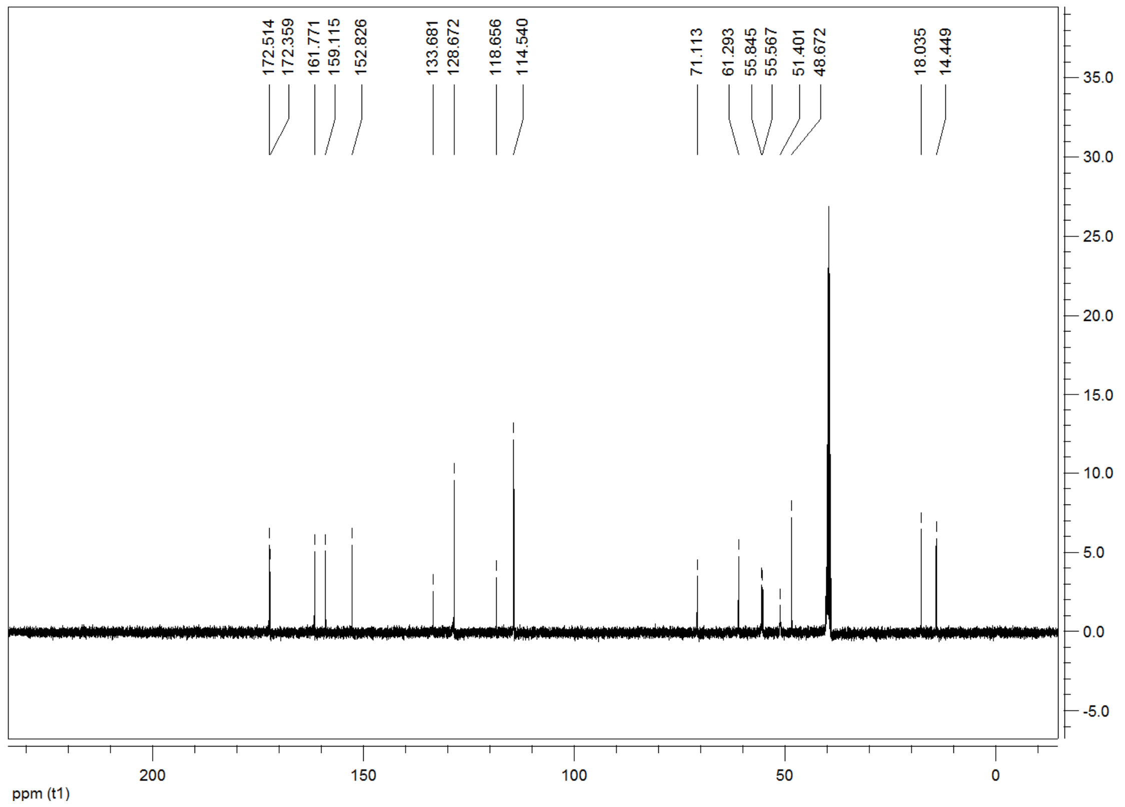
172.514
172.359
161.771
159.115
152.826
133.681
128.672
118.656
114.540
71.113
61.293
55.845
55.567
51.401
48.672
18.035
14.449
ppm (t1)

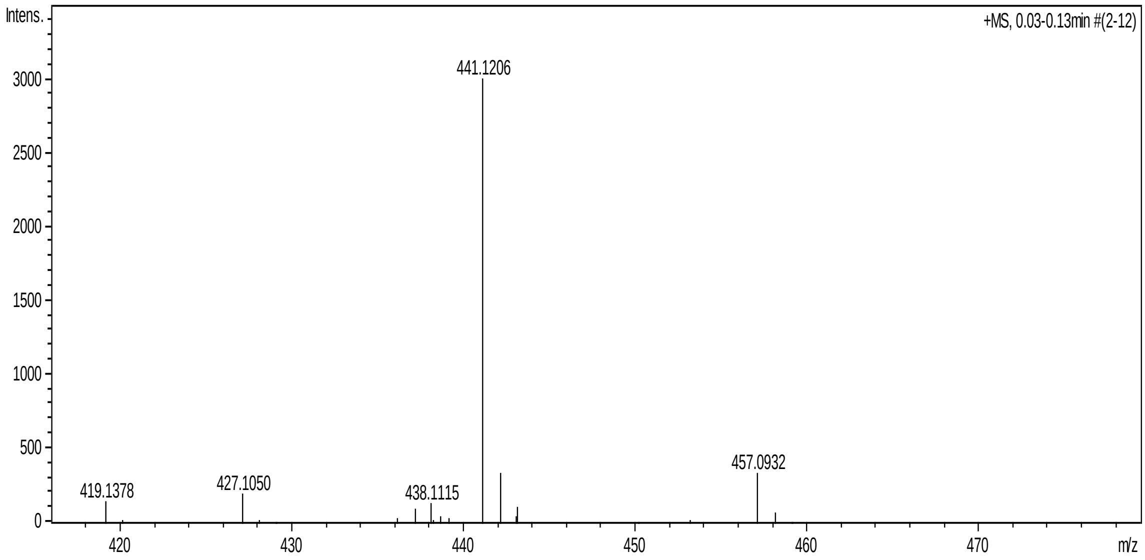
Intens.
+MS, 0.03-0.13min #(2-12)
441.1206
457.0932
419.1378
427.1050
438.1115
m/z

Ethyl ((5-amino-4-cyano-3-(3-nitrophenyl)-2,3-dihydrothiophene-2-carbonyl)carbamoyl)alaninate (2b): white solid, 55%, m.p. 248-250℃; ^{1}H NMR (400 MHz, DMSO-d_6) δ: 10.56 (s, 1H, NH), 8.54 (s, 1H, NH), 8.23-8.17 (m, 2H, ArH,), 7.81 (d, J = 7.2 Hz, 1H, ArH), 7.72-7.68 (m, 1H, ArH), 7.37 (s, 2H, NH_2), 4.74 (s, 1H, CH), 4.34-4.30 (m, 1H, CH), 4.16-4.11 (m, 3H, CH), 1.37 (d, J = 6.4 Hz, 3H, CH_3), 1.20 (t, J = 7.2 Hz, 3H, CH_3); ^{13}C NMR (100 MHz, DMSO-d6) δ: 172.5, 172.0, 163.0, 152.8, 148.4, 144.1, 134.6, 134.5, 134.4, 134.3, 134.4, 134.3, 130.7, 130.6, 130.5, 130.4, 123.0, 122.9, 122.3, 122.2, 118.3, 69.8, 61.2, 61.1, 61.0, 55.5, 55.4, 55.3, 55.2, 51.0, 50.9, 48.6, 48.5, 18.1, 18.0, 14.4, 14.3; IR (KBr) υ: 3440, 3329, 3221, 3138, 2974, 2921, 2851, 2183, 1738, 1692, 1632, 1585, 1530, 1348, 1251, 1200, 1140, 987, 922, 868 cm^{-1}; MS (m/z): HRMS (ESI) Calcd. for $C_{18}H_{19}N_5O_6S$ ($[M+Na]^+$): 456.1056, found: 456.0954.

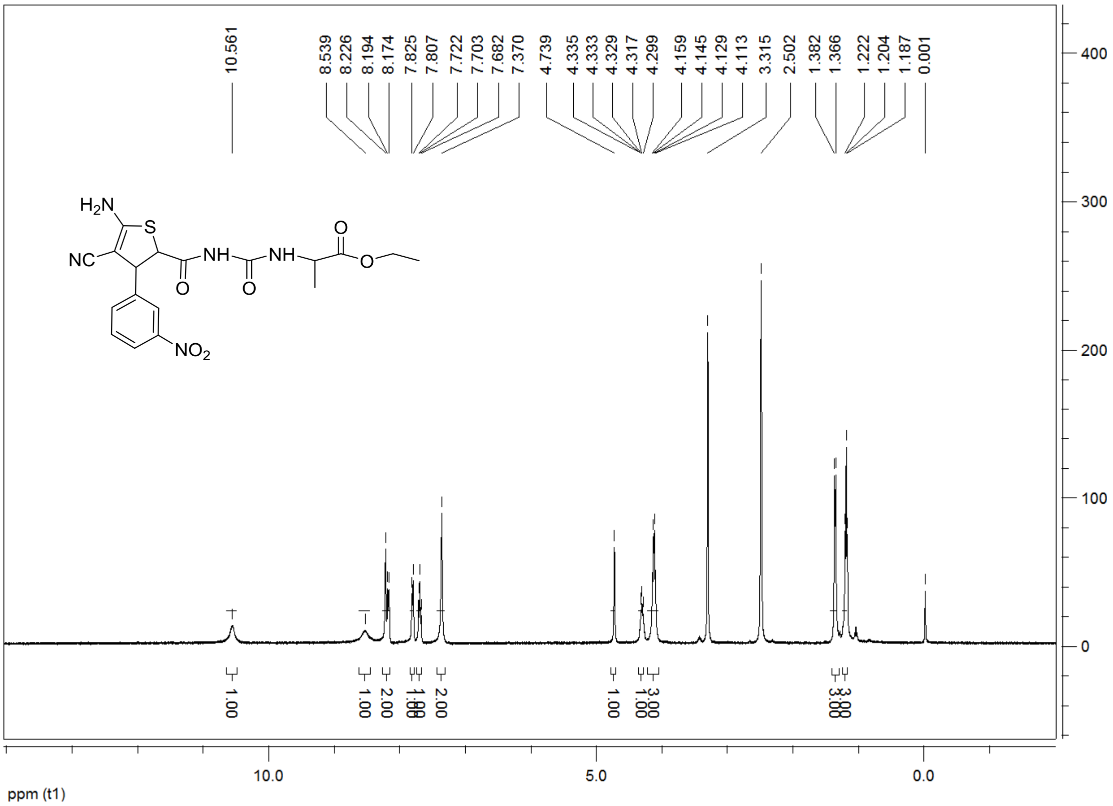

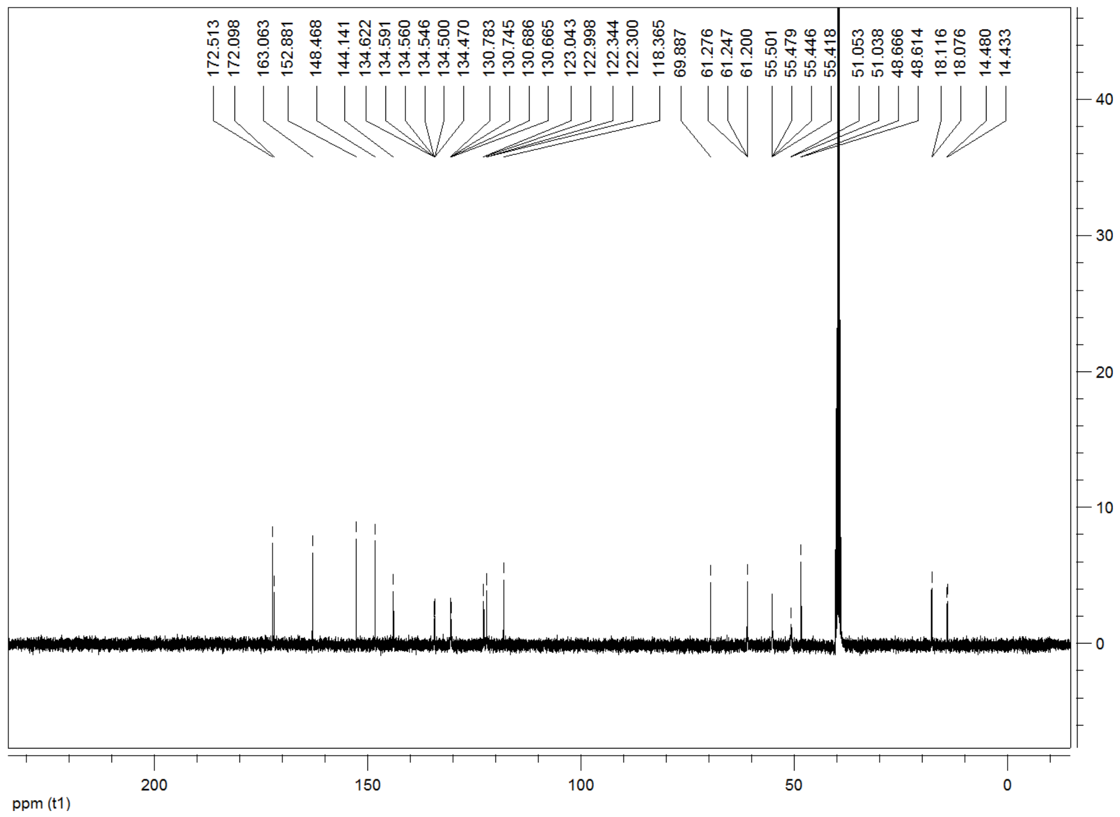

172.513
172.098
163.063
152.881
148.468
144.141
134.622
134.591
134.560
134.546
134.500
134.470
130.783
130.745
130.686
130.665
123.043
122.998
122.344
122.300
118.365
69.887
61.276
61.247
61.200
55.501
55.479
55.446
55.418
51.053
51.038
48.666
48.614
18.116
18.076
14.480
14.433
ppm (t1)

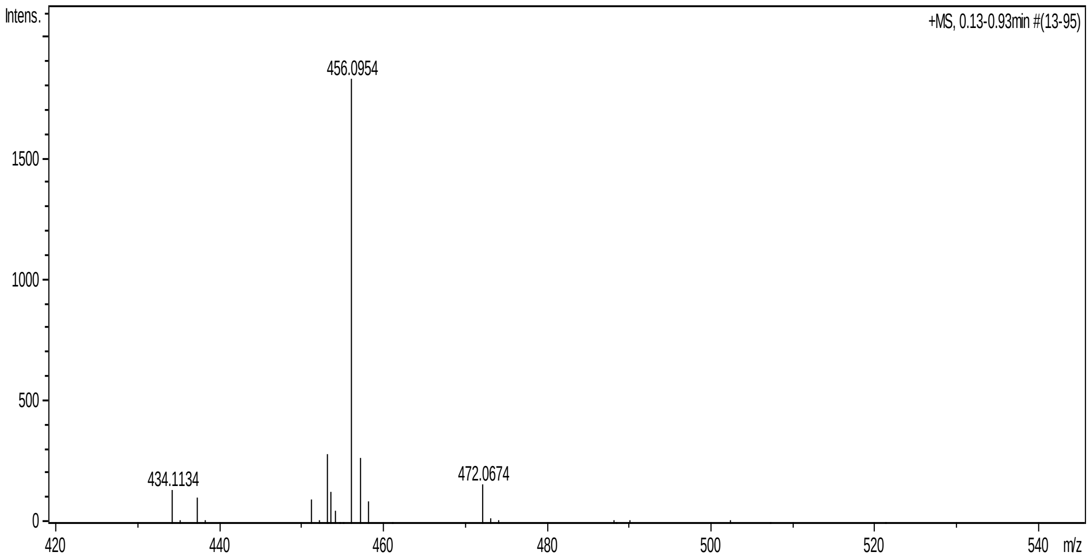

Intens.
+MS, 0.13-0.93min #(13-95)
456.0954
434.1134
472.0674
m/z

Ethyl ((5-amino-4-cyano-3-(p-tolyl)thiophene-2-carbonyl)carbamoyl)serinate (2c): white solid, 63%, m.p. 187-189℃; ^{1}H NMR (400 MHz, DMSO-d_6) δ: 10.61 (s, 1H, NH), 8.74 (s, 1H, NH), 7.20 (s, 2H, NH_2), 7.18 (brs, 4H, ArH), 5.29-5.26 (m, 1H, CH), 4.51 (d, J = 2.8 Hz, 1H, CH), 4.34-4.31 (m, 1H, CH), 4.16-4.11 (m, 2H, CH), 4.08 (s, 1H, OH), 3.83-3.78 (m, 1H, CH), 3.70-3.67 (m, 1H, CH), 2.29 (s, 3H, CH_3), 1.20 (t, J = 6.8 Hz, 3H, CH_3); ^{13}C NMR (100 MHz, DMSO-d6) δ: 172.2, 170.6, 161.9, 153.1, 138.8, 137.1, 129.7, 127.4, 118.6, 109.9, 70.8, 61.5, 61.2, 55.6, 55.2, 51.8, 51.7, 21.0, 14.5; IR (KBr) υ: 3409, 3358, 3293, 3107, 3093, 2977, 2208, 1764, 1651, 1594, 1546, 1388, 1245, 1280, 1138, 1027, 957, 816 cm^{-1}; MS (m/z): HRMS (ESI) Calcd. for $C_{19}H_{20}NaN_4O_5S$ ([M+Na]$^+$): 441.1203, found: 441.1188.

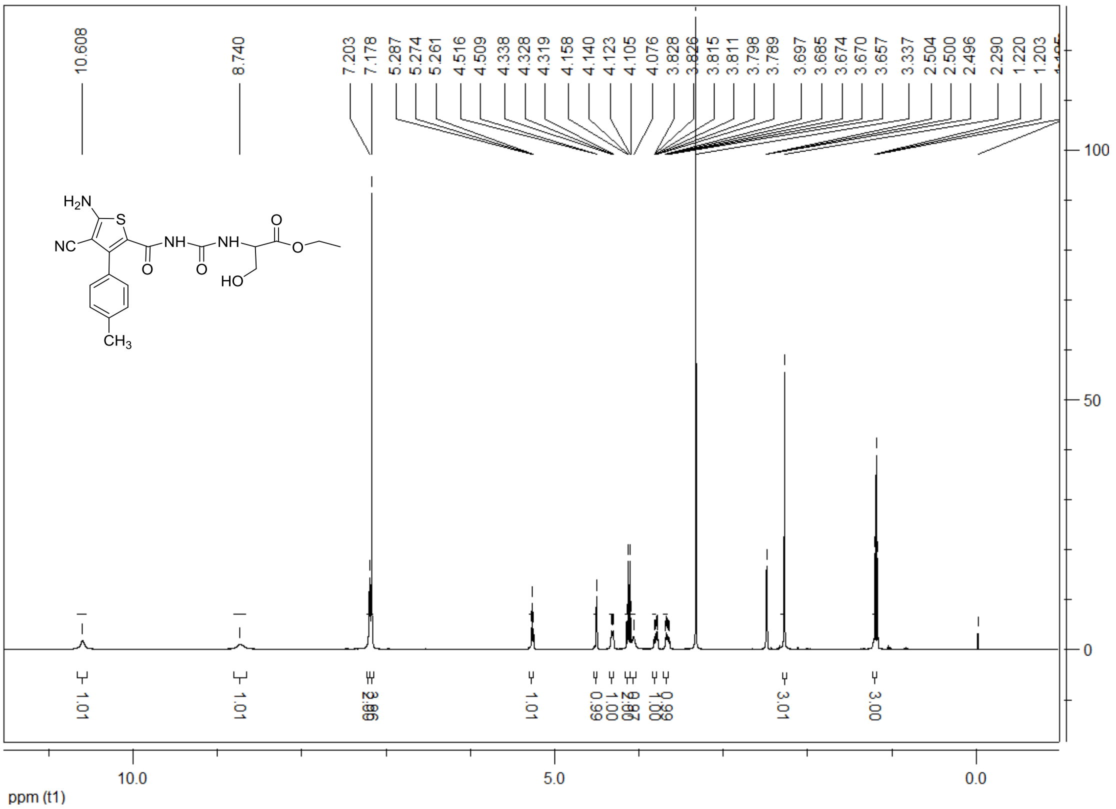

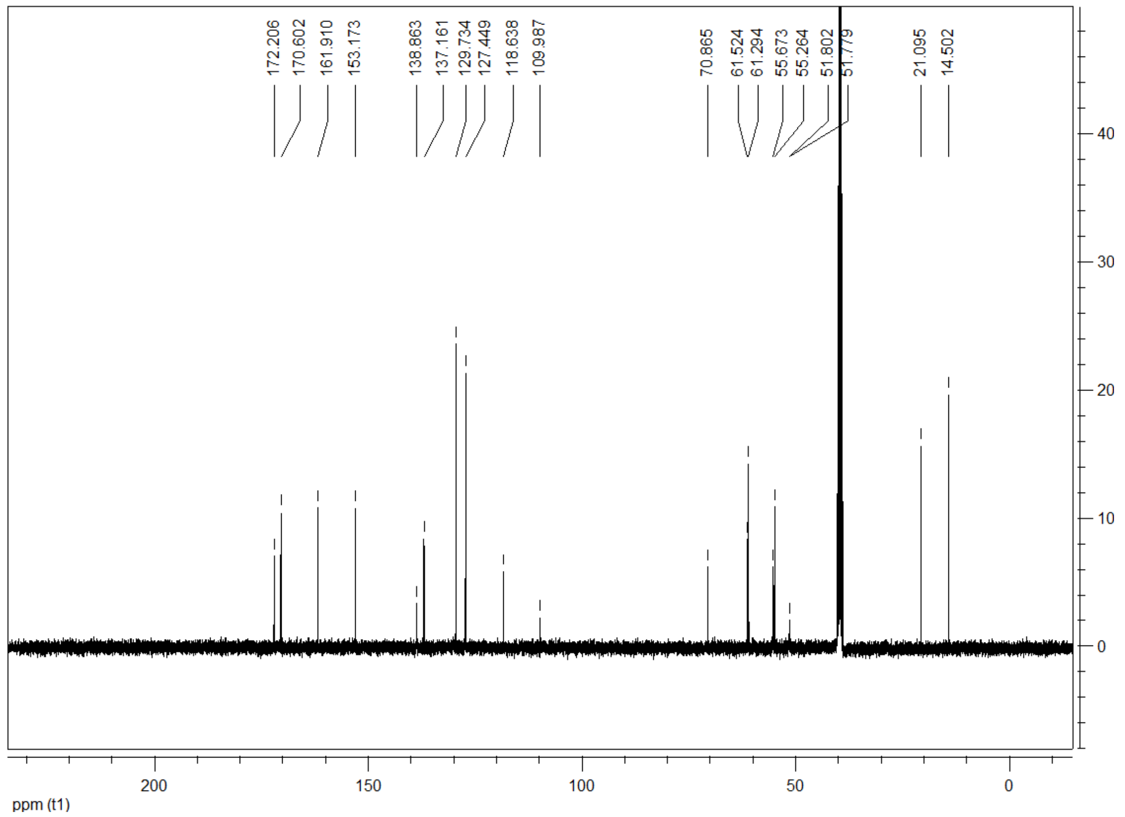
172.206
170.602
161.910
153.173
138.863
137.161
129.734
127.449
118.638
109.987
70.865
61.524
61.294
55.673
55.264
51.802
51.779
21.095
14.502
ppm (t1)

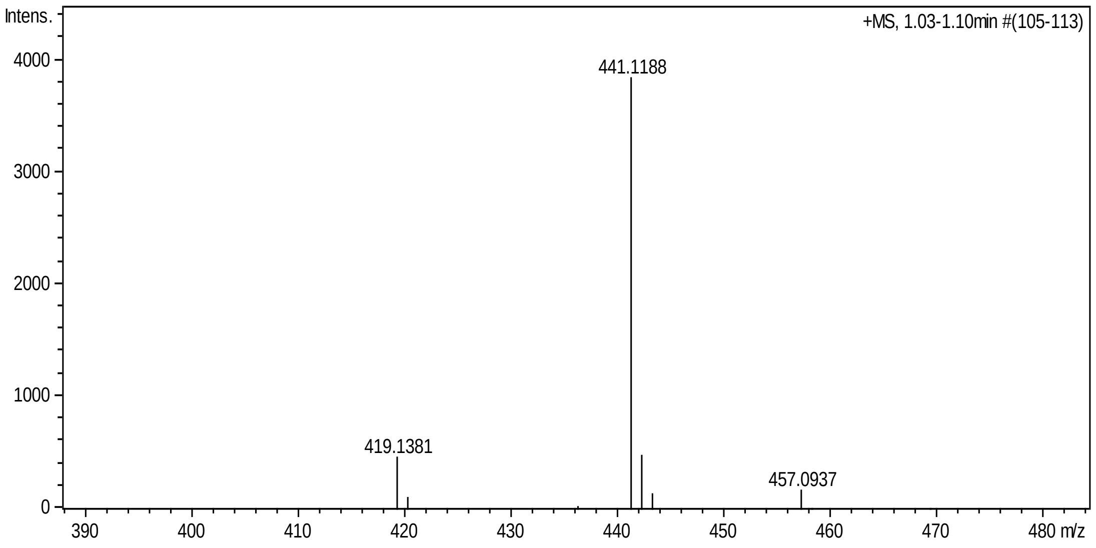
Intens.
+MS, 1.03-1.10min #(105-113)
441.1188
419.1381
457.0937
m/z

Ethyl 2-(3-(5-amino-4-cyano-3-(4-methoxyphenyl)-2,3-dihydrothiophene-2-carbonyl)ureido)-3-methylpentanoate (2d): yellow solid, 68%, m.p. 188-190℃; ^{1}H NMR (400 MHz, DMSO-d_6) δ: 10.60 (s, 1H, NH), 8.58 (s, 1H, NH), 7.22 (d, *J* = 8.4 Hz, 2H, ArH), 7.17 (s, 2H, NH_2), 6.92 (d, *J* = 8.4 Hz, 2H, ArH), 4.51-4.50 (m, 1H, CH), 4.28-4.25 (m, 1H, CH), 4.20-4.06 (m, 3H, CH), 3.75 (s, 3H, OCH_3), 1.89-1.86 (m, 1H, CH), 1.44-1.37 (m, 1H, CH), 1.26-1.24 (m, 1H, CH), 1.21 (t, *J* = 7.2 Hz, 3H, CH_3), 0.91-0.88 (m, 6H, CH_3); ^{13}C NMR (100 MHz, DMSO-d6) δ: 172.7, 171.3, 161.7, 159.1, 153.2, 133.7, 128.6, 118.6, 114.5, 71.2, 61.2, 57.0, 55.8, 55.5, 51.3, 37.3, 25.2, 15.9, 14.4, 11.7; IR (KBr) υ: 3323, 3227, 2967, 2186, 1701, 1635, 1580, 1544, 1511, 1346, 1250, 1181, 1028, 831 cm^{-1}; MS (*m/z*): HRMS (ESI) Calcd. for $C_{22}H_{28}N_4O_5S$ ($[M+H]^+$): 461.1780, found: 461.1848.

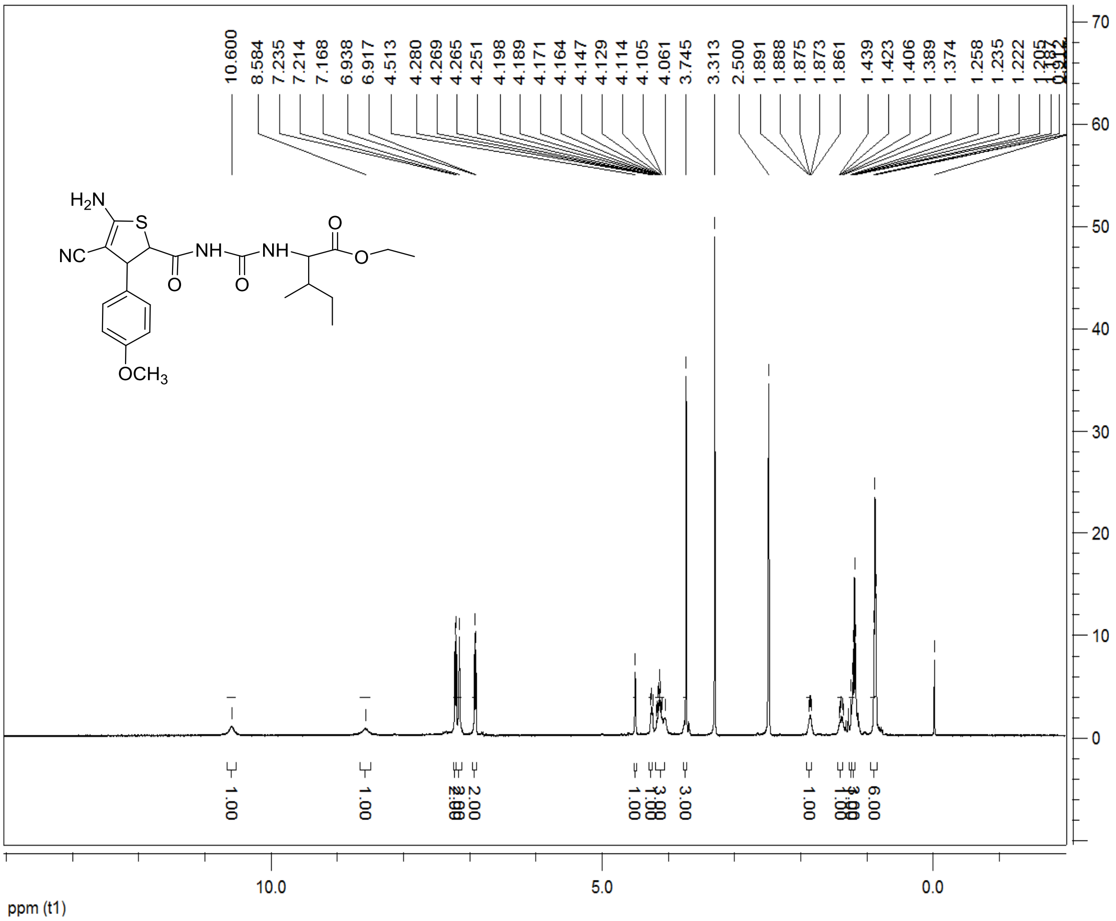

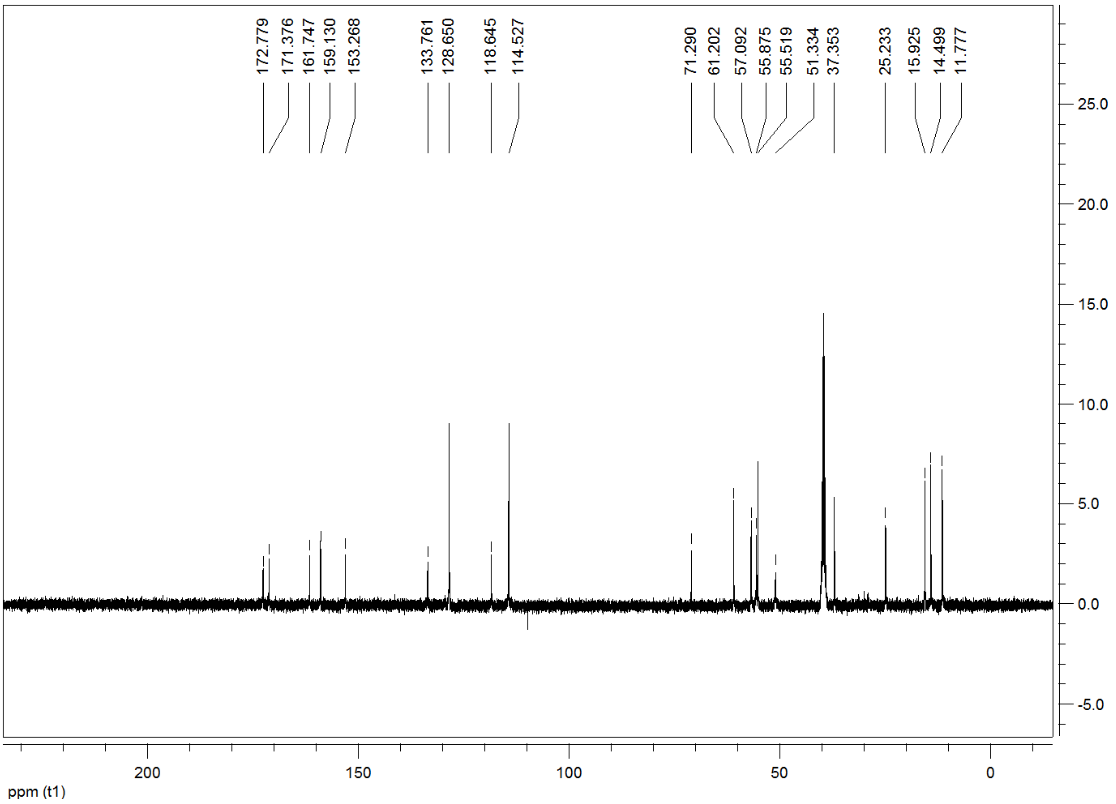
172.779
171.376
161.747
159.130
153.268
133.761
128.650
118.645
114.527
71.290
61.202
57.092
55.875
55.519
51.334
37.353
25.233
15.925
14.499
11.777
25.0
20.0
15.0
10.0
5.0
0.0
-5.0
200
150
100
50
0
ppm (t1)

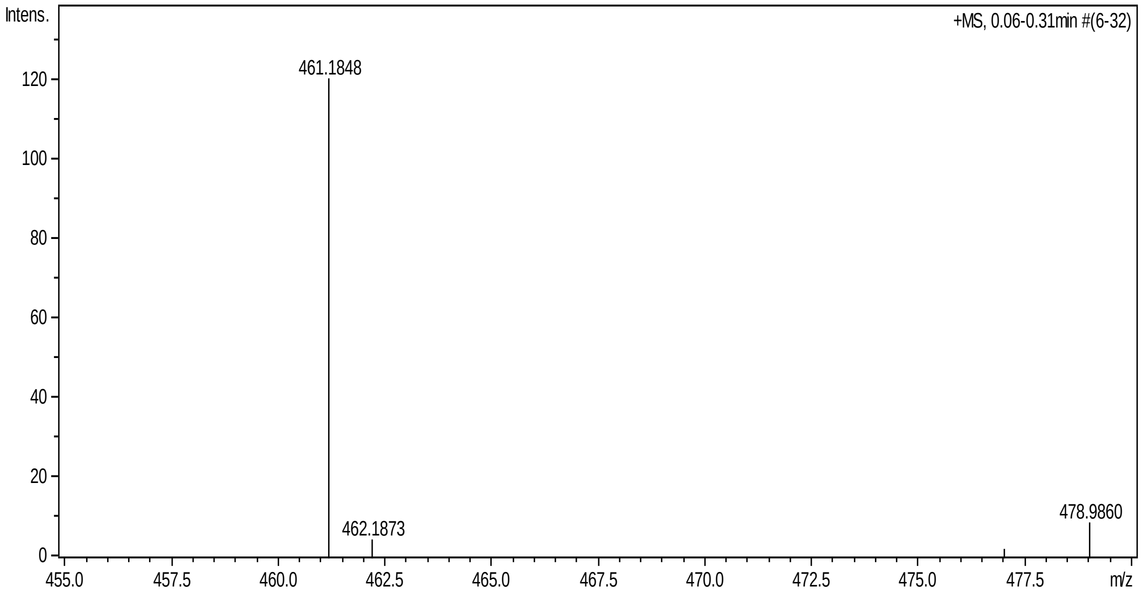
Intens.
+MS, 0.06-0.31min #(6-32)
461.1848
462.1873
478.9860
120
100
80
60
40
20
0
455.0
457.5
460.0
462.5
465.0
467.5
470.0
472.5
475.0
477.5
m/z

Ethyl 2-(3-(5-amino-4-cyano-3-(p-tolyl)-2,3-dihydrothiophene-2-carbonyl)ureido)-3-methylpentanoate (2e): yellow solid, 64%, m.p. 190-192℃; ^{1}H NMR (400 MHz, DMSO-d_6) δ: 10.60 (s, 1H, NH), 8.57 (s, 1H, NH), 7.17-7.08 (m, 6H, ArH, NH_2), 4.52 (s, 1H, CH), 4.26 (s, 1H, CH), 4.15-4.07 (m, 3H, CH), 2.29-2.23 (m, 3H, CH_3), 1.87 (s, 1H, CH), 1.40 (m, 1H, CH), 1.22-1.18 (m, 3H, CH_3), 1.16-1.11 (m, 1H, CH), 0.89-0.80 (m, 6H, CH_3); ^{13}C NMR (100 MHz, DMSO-d6) δ: 172.5, 172.0, 163.0, 152.8, 148.4, 144.1, 134.5, 130.7, 123.0, 122.3, 118.3, 69.9, 61.2, 55.4, 50.9, 48.6, 18.0, 14.4; IR (KBr) υ: 3447, 3329, 3225, 2968, 2376, 2339, 2186, 1702, 1634, 1544, 1474, 1349, 1195, 1149, 1023, 859, 810 cm^{-1}; MS (*m*/*z*): HRMS (ESI) Calcd. for $C_{22}H_{28}N_4O_4SNa$ ([M+Na]$^+$): 485.2071, found: 467.1735.

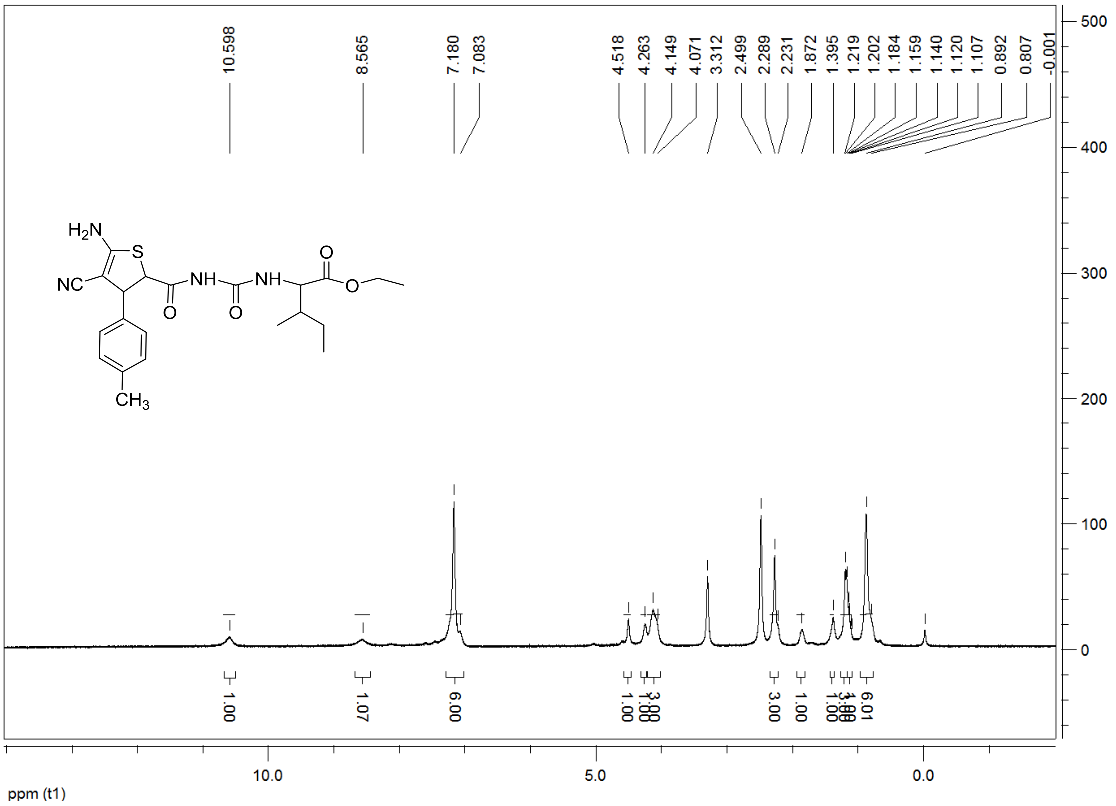

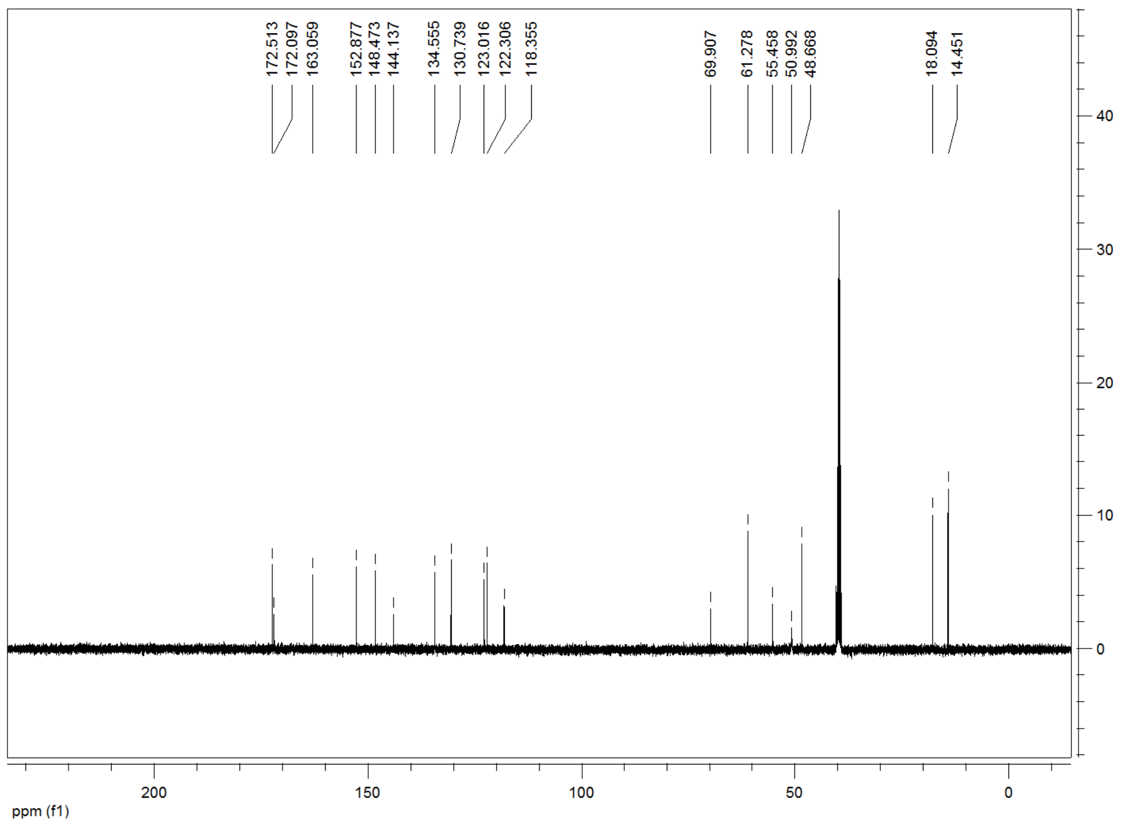
172.513
172.097
163.059
152.877
148.473
144.137
134.555
130.739
123.016
122.306
118.355
69.907
61.278
55.458
50.992
48.668
18.094
14.451
ppm (f1)

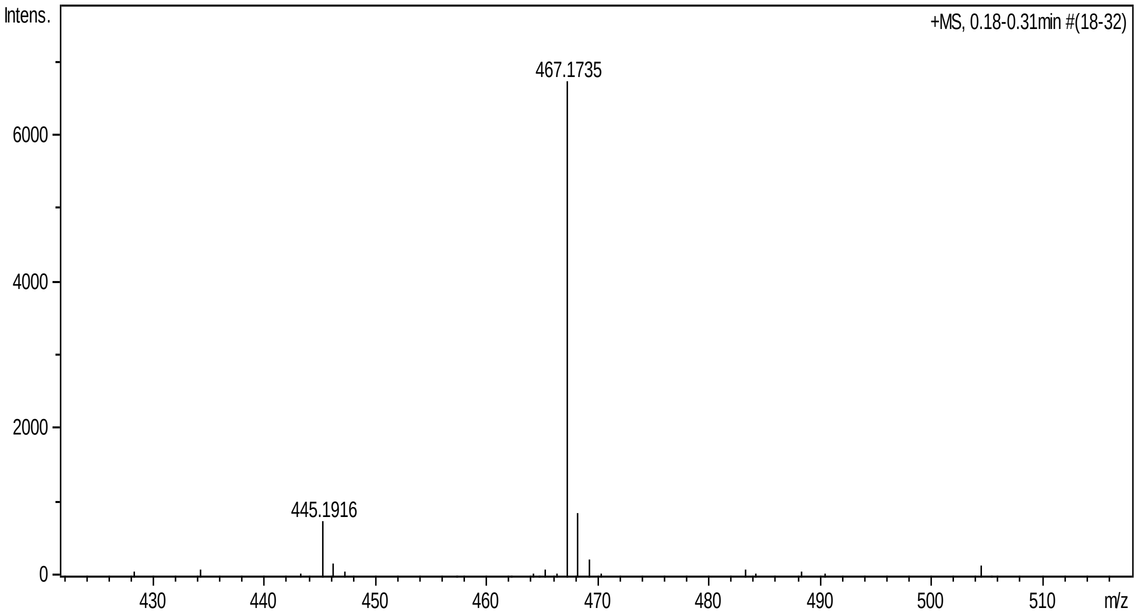
Intens.
+MS, 0.18-0.31min #(18-32)
467.1735
445.1916
m/z

Ethyl ((5-amino-4-cyano-3-phenyl-2,3-dihydrothiophene-2-carbonyl)carbamoyl)phenylalaninate (2f): yellow solid, 48%, m.p. 185-187℃; ^{1}H NMR (400 MHz, DMSO-d6) δ: 10.55 (s, 1H, NH), 8.48 (s, 1H, NH), 7.39-7.35 (m, 2H, ArH), 7.33-7.29 (m, 5H, ArH), 7.25 (d, *J* = 7.2 Hz, 1H, ArH), 7.21-7.17 (m, 4H, ArH, NH_2), 4.58-4.53 (m, 2H, CH), 4.12-4.06 (m, 3H, CH), 3.12-3.04 (m, 2H, CH), 1.15 (t, *J* = 6.8 Hz, 3H, CH_3); ^{13}C NMR (100 MHz, DMSO-d6) δ: 172.2, 171.2, 162.0, 152.9, 141.9, 136.7, 129.6, 129.1, 128.8, 127.9, 127.5, 127.2, 118.6, 70.8, 61.3, 55.6, 54.3, 54.2, 51.9, 37.4, 14.3; IR (KBr) υ: 3414, 3302, 3220, 3079, 2974, 2182, 1751, 1688, 1631, 1591, 1555, 1503, 1453, 1345, 1301, 1247, 1202, 1107, 1040, 975, 856, 813 cm^{-1}; MS (*m/z*): HRMS (ESI) Calcd. for $C_{24}H_{24}N_4O_4S$ ($[M+Na]^+$): 487.1518, found: 487.1412.

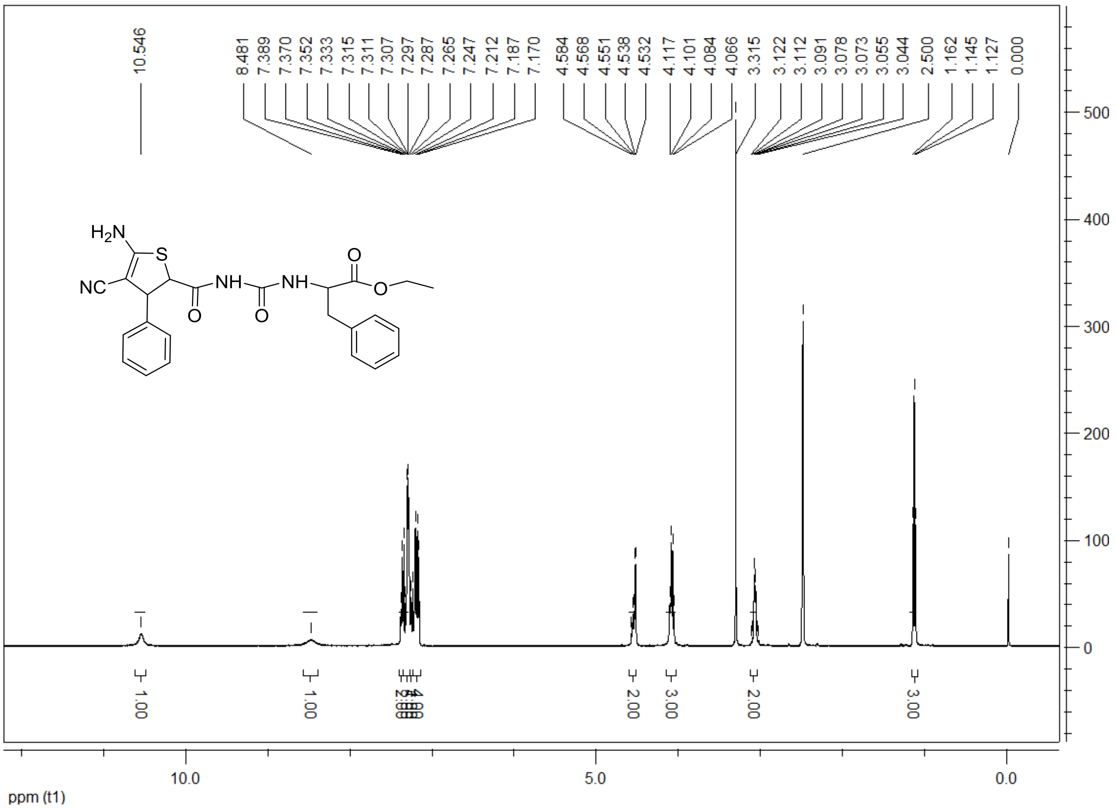

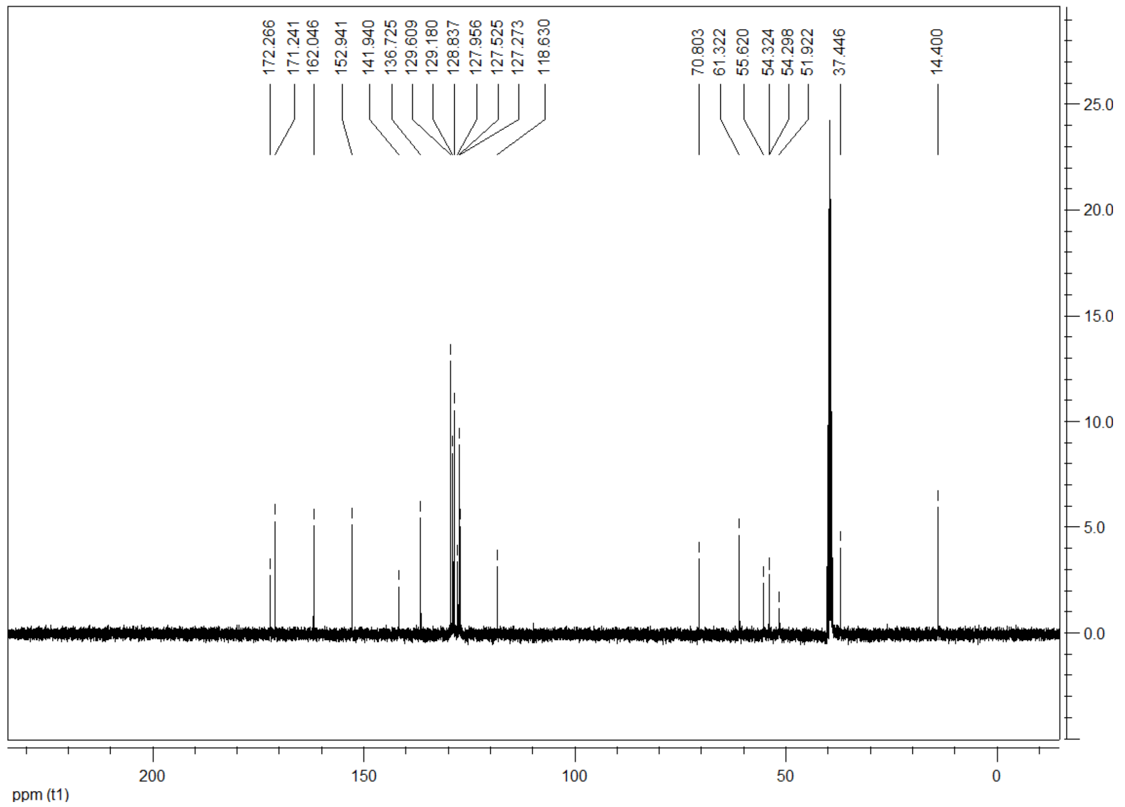
172.266
171.241
162.046
152.941
141.940
136.725
129.609
129.180
128.837
127.956
127.525
127.273
118.630
70.803
61.322
55.620
54.324
54.298
51.922
37.446
14.400
25.0
20.0
15.0
10.0
5.0
0.0
200
150
100
50
0
ppm (t1)

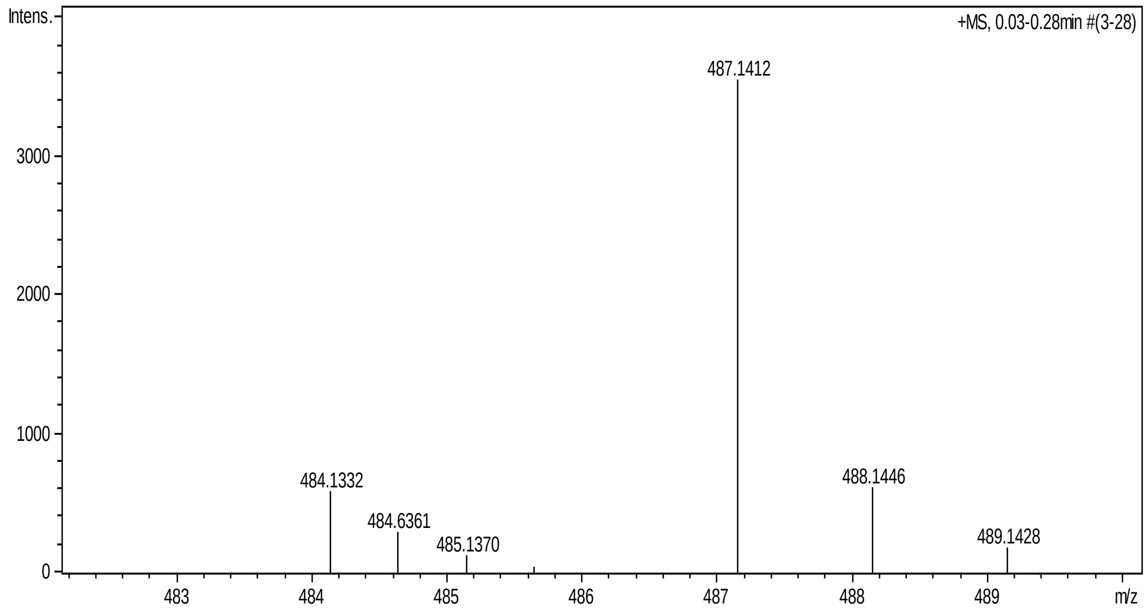
Intens.
+MS, 0.03-0.28min #(3-28)
487.1412
484.1332
484.6361
485.1370
488.1446
489.1428
3000
2000
1000
0
483
484
485
486
487
488
489
m/z

ethyl ((5-amino-4-cyano-3-(4-methoxyphenyl)-2,3-dihydrothiophene-2-carbonyl)carbamoyl)phenylalaninate (2g): yellow solid, 62%, m.p. 163-165℃; ^{1}H NMR (400 MHz, DMSO-d_6) δ: 10.56 (s, 1H, NH), 8.50 (s, 1H, NH), 7.33-7.30 (m, 2H, ArH), 7.27-7.24 (m, 1H, ArH), 7.21-7.17 (m, 6H, ArH, NH_2), 6.94-6.91 (m, 2H, ArH), 4.58-4.53 (m, 1H, CH), 4.49-4.46 (m, 1H, CH), 4.13-4.04 (m, 3H, CH), 3.75 (s, 3H, OCH_3), 3.11-3.04 (m, 2H, CH), 1.15 (t, J = 7.2 Hz, 3H, CH_3); *dr*-isomer: 3.74 (s, 3H, OCH_3), 1.14 (t, J = 7.2 Hz, 3H, CH_3). *dr*-ratio = 0.53:0.47. ^{13}C NMR (100 MHz, DMSO-d6) δ: 172.2, 171.2, 171.1, 161.7, 161.6, 159.1, 152.9, 152.8, 136.7, 136.6, 133.7, 133.6, 129.6, 128.8, 128.6, 127.2, 118.6, 114.5, 71.1, 61.3, 55.8, 55.7, 55.5, 54.3, 54.2, 51.4, 51.3, 51.2, 37.4, 37.3, 14.4; IR (KBr) υ: 3444, 3315, 3220, 3145, 2967, 2838, 2179, 1747, 1693, 1623, 1580, 1540, 1502, 1349, 1302, 1250, 1182, 1117, 1034, 977, 886, 828 cm^{-1}; MS (m/z): HRMS (ESI) Calcd. for $C_{25}H_{26}N_4O_5S$ ($[M+H]^+$): 495.1624, found: 495.1692.

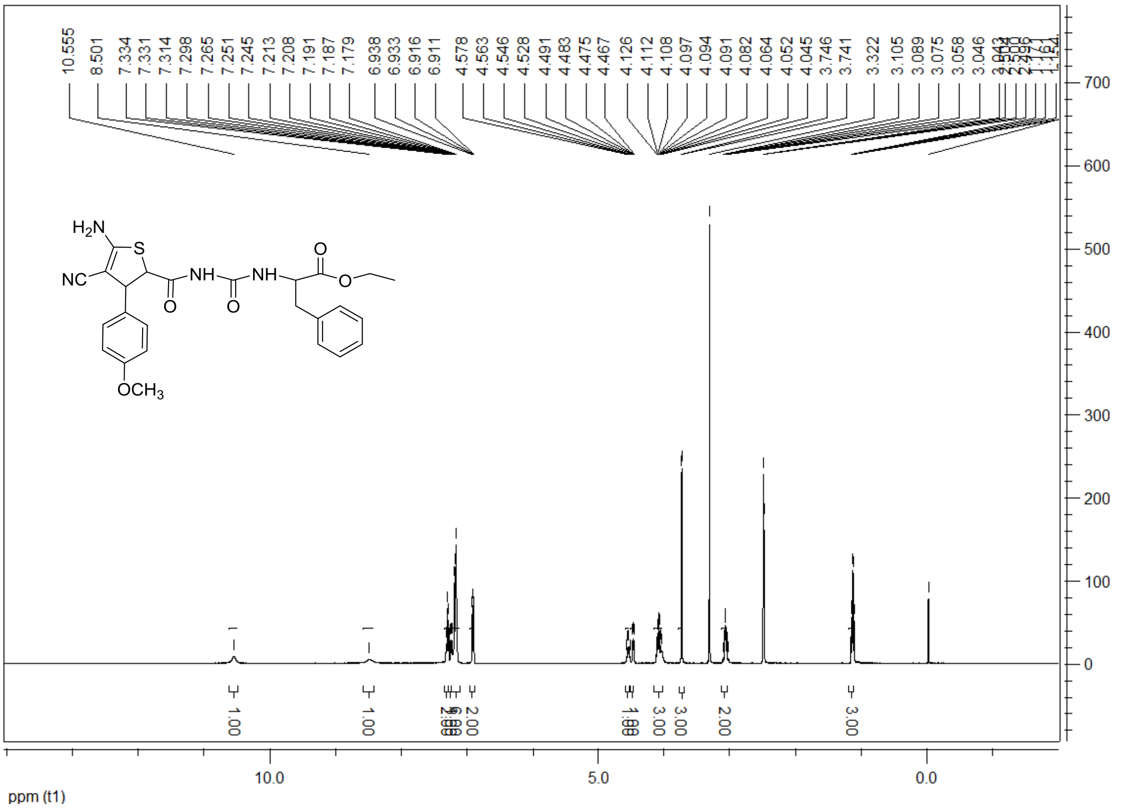

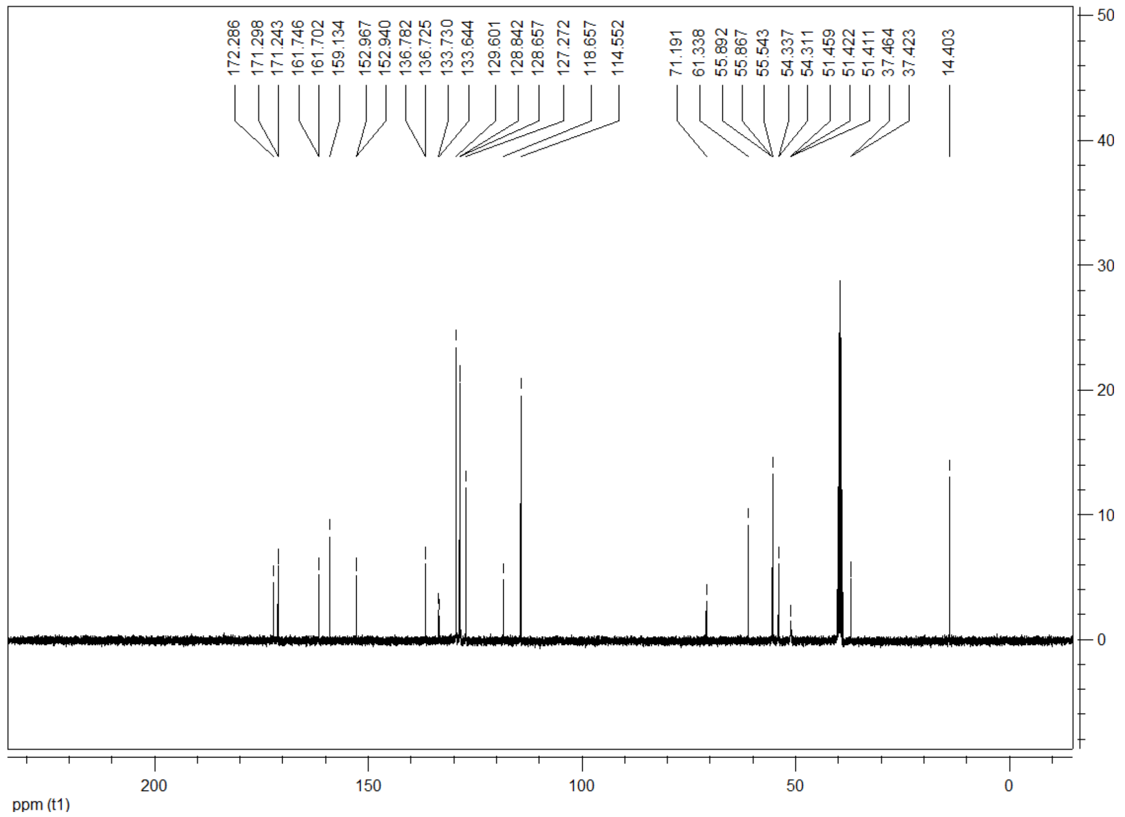

172.286
171.298
171.243
161.746
161.702
159.134
152.967
152.940
136.782
136.725
133.730
133.644
129.601
128.842
128.657
127.272
118.657
114.552
71.191
61.338
55.892
55.867
55.543
54.337
54.311
51.459
51.422
51.411
37.464
37.423
14.403
ppm (t1)

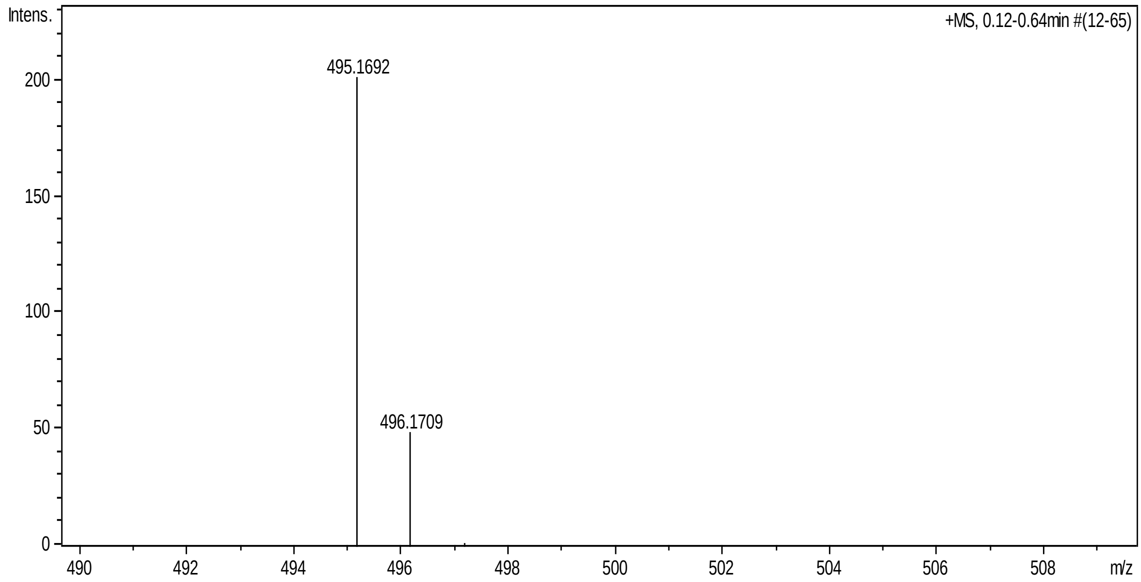

Intens.
+MS, 0.12-0.64min #(12-65)
495.1692
496.1709
m/z

Ethyl ((5-amino-4-cyano-3-(3-methoxyphenyl)-2,3-dihydrothiophene-2-carbonyl)carbamoyl)phenylalaninate (2h): yellow solid, 60%, m.p. 183-185℃; ^{1}H NMR (400 MHz, DMSO-d_6) δ: 10.55 (s, 1H, NH), 8.50 (s, 1H, NH), 7.33-7.30 (m, 4H, ArH), 7.21-7.17 (m, 4H, ArH, NH_2), 6.89-6.86 (m, 3H, ArH), 4.60-4.55 (m, 1H, CH), 4.51-4.49 (m, 1H, CH), 4.13-4.07 (m, 3H, CH), 3.75 (s, 3H, OCH_3), 3.11-3.06 (m, 2H, CH), 1.15 (t, J = 7.2 Hz, 3H, CH_3); *dr*-isomer: 3.74 (s, 3H, OCH_3), 1.14 (t, J = 7.2 Hz, 3H, CH_3). *dr*-ratio = 0.51:0.49. ^{13}C NMR (100 MHz, DMSO-d6) δ: 172.2, 172.1, 171.3, 171.2, 162.1, 162.0, 159.9, 153.0, 152.9, 143.5, 143.4, 136.7, 136.6, 130.2, 129.6, 129.5, 128.8, 127.2, 119.5, 118.6, 113.5, 113.0, 70.6, 70.5, 61.3, 61.2, 55.5, 55.4, 55.3, 54.3, 54.2, 54.1, 51.8, 51.7, 37.4, 37.3, 14.4; IR (KBr) υ: 3439, 3305, 3221, 3146, 2964, 2835, 2181, 1750, 1695, 1627, 1585, 1539, 1492, 1348, 1303, 1246, 1184, 1044, 975, 868 cm^{-1}; MS (m/z): HRMS (ESI) Calcd. for $C_{25}H_{26}N_4O_5S$ ($[M+H]^+$): 495.1624, found: 495.1699.

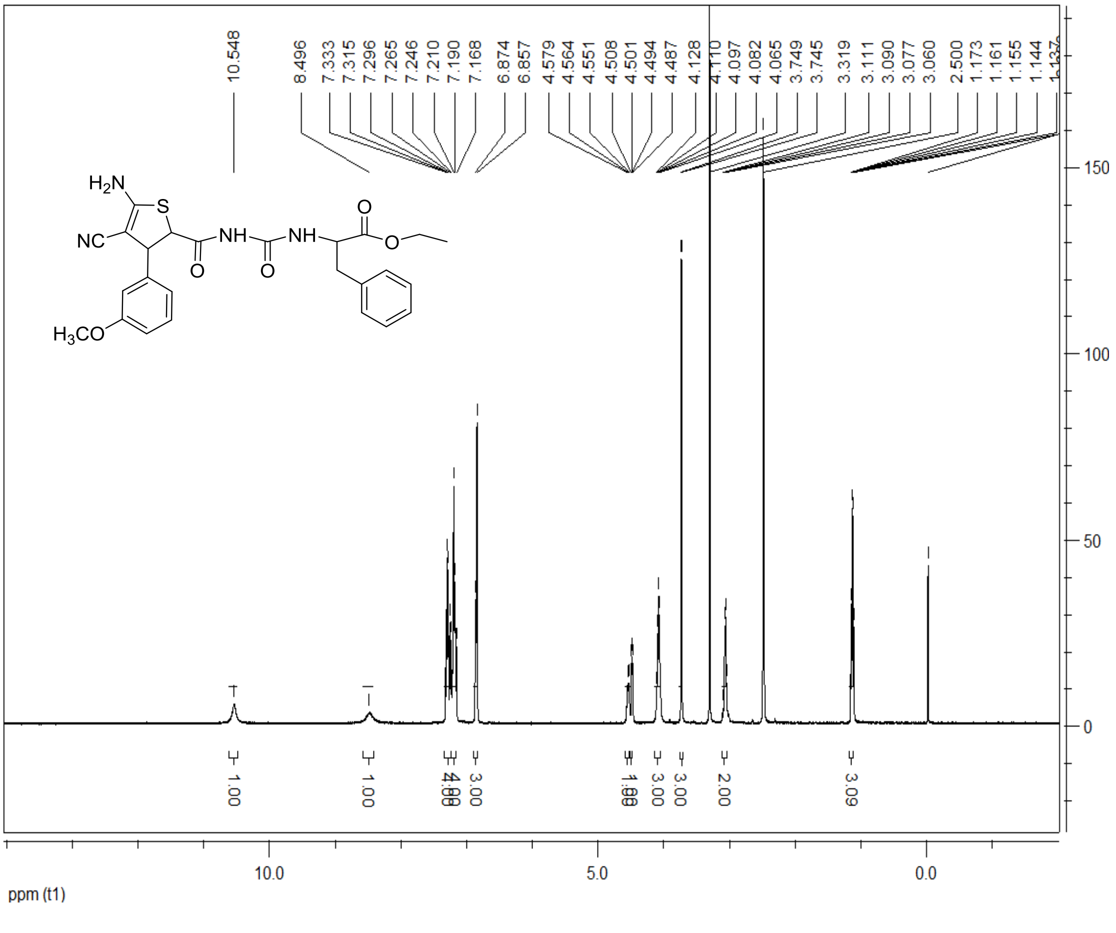

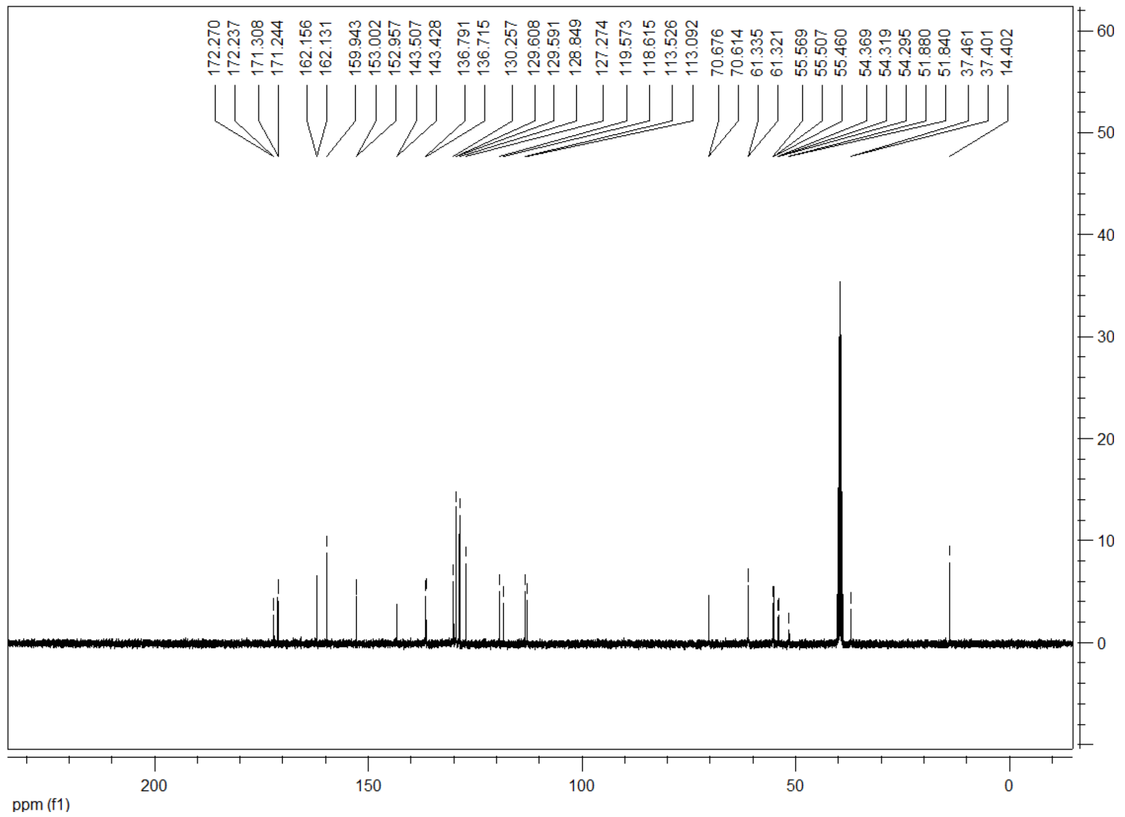
172.270
172.237
171.308
171.244
162.156
162.131
159.943
153.002
152.957
143.507
143.428
136.791
136.715
130.257
129.608
129.591
128.849
127.274
119.573
118.615
113.526
113.092
70.676
70.614
61.335
61.321
55.569
55.507
55.460
54.369
54.319
54.295
51.880
51.840
37.461
37.401
14.402
ppm (f1)

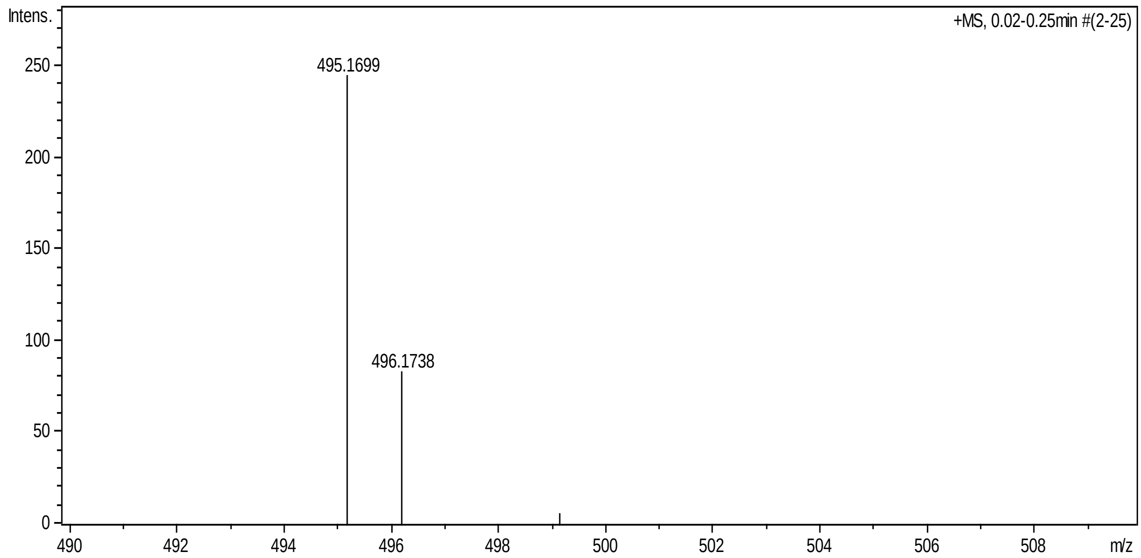
Intens.
+MS, 0.02-0.25min #(2-25)
495.1699
496.1738
m/z

ethyl ((5-amino-4-cyano-3-(2-methoxyphenyl)-2,3-dihydrothiophene-2-carbonyl)carbamoyl)phenylalaninate (2i): yellow solid, 56%, m.p. 187-189℃; ^{1}H NMR (400 MHz, DMSO-*d*$_6$) δ: 10.51 (s, 1H, NH), 8.54 (s, 1H, NH), 7.34-7.19 (m, 8H, ArH, NH_2), 7.13 (d, *J* = 7.2 Hz, 1H, ArH) 7.05-6.97 (m, 2H, ArH), 4.79 (s, 1H, CH), 4.57 (s, 1H, CH), 4.14-4.09 (m, 2H, CH), 3.89 (s, 1H, CH), 3.76 (s, 3H, OCH_3), 3.16-3.06 (m, 2H, CH), 1.17 (t, *J* = 6.8 Hz, 3H, CH_3); *dr*-isomer: 3.74 (s, 3H, OCH_3), 1.16 (t, *J* = 6.8 Hz, 3H, CH_3). *dr*-ratio = 0.51:0.49. ^{13}C NMR (100 MHz, DMSO-d6) δ: 172.8, 172.7, 172.6, 171.2, 162.6, 156.6, 153.0, 152.9, 136.8, 136.7, 129.6, 129.1, 129.0, 128.8, 128.7, 127.2, 127.1, 120.8, 118.6, 111.5, 68.1, 61.3, 61.2, 56.0, 55.9, 54.5, 54.4, 54.3, 45.9, 37.3, 14.4; IR (KBr) υ: 3408, 3315, 3229, 2966, 2839, 2174, 1736, 1697, 1638, 1584, 1535, 1493, 1354, 1247, 1187, 1108, 1026, 982, 892, 854 cm^{-1}; MS (*m/z*): HRMS (ESI) Calcd. for $C_{25}H_{26}N_4O_5S$ ([M+H]$^+$): 495.1624, found: 495.1699.

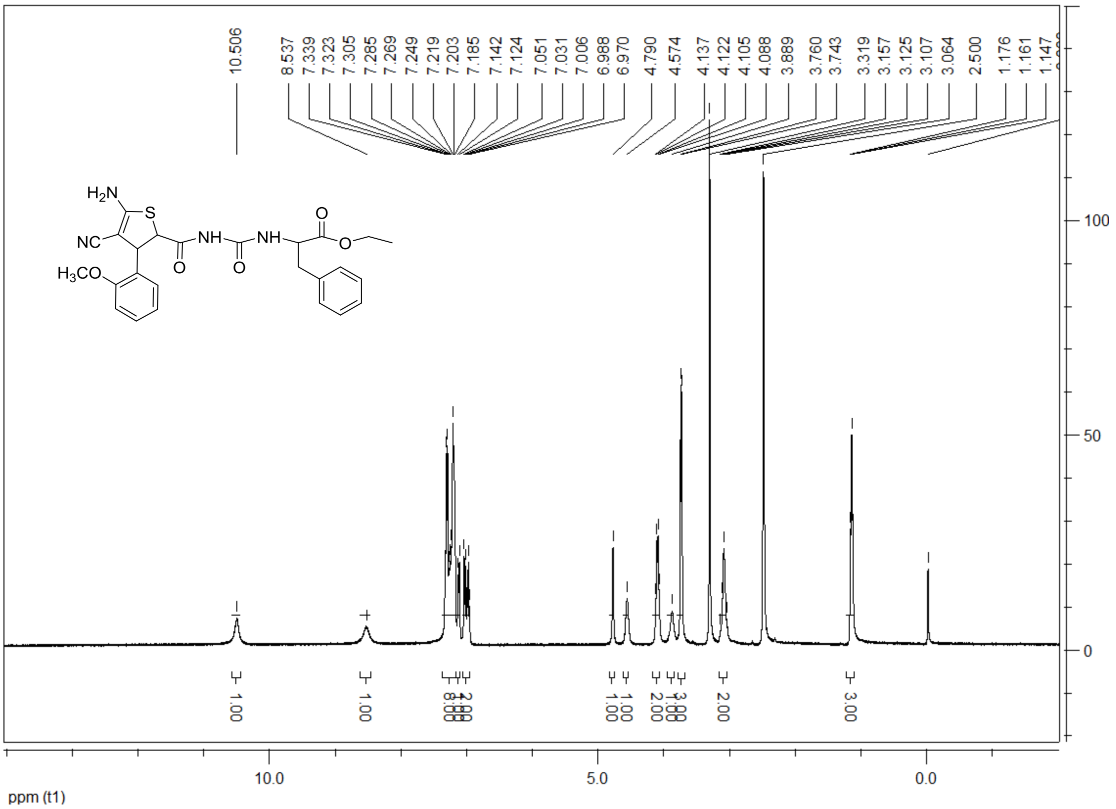

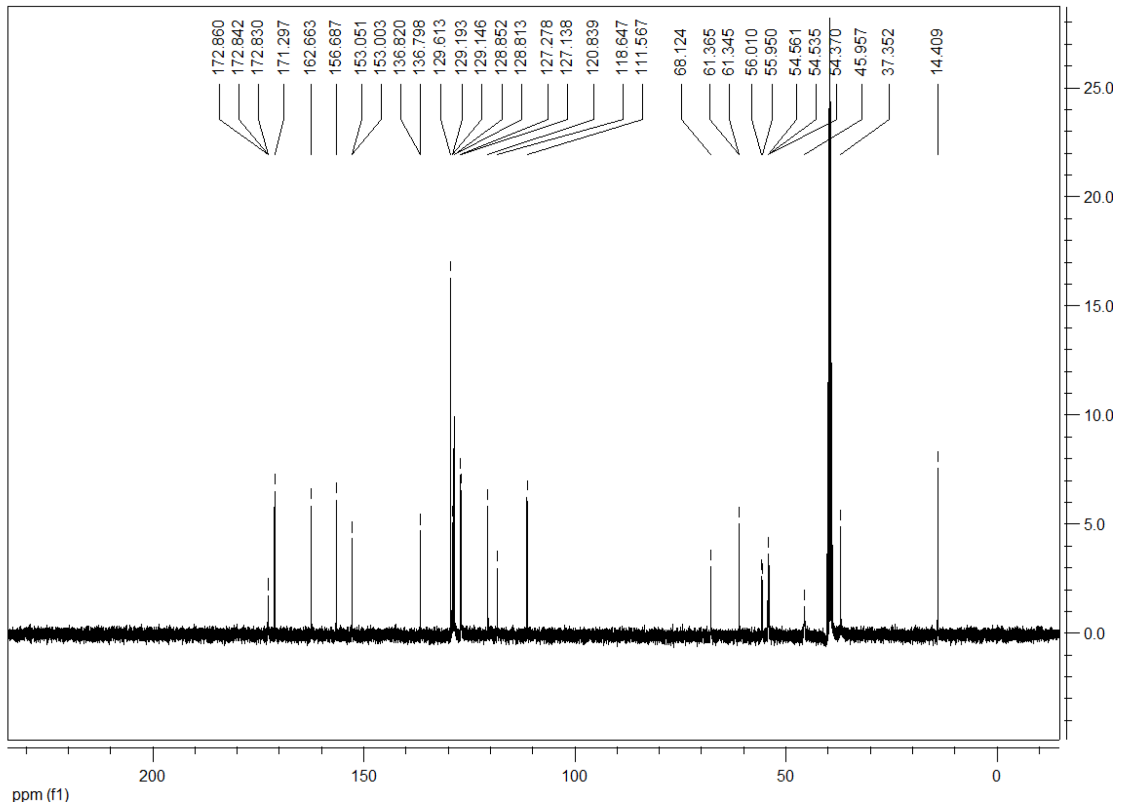
172.860
172.842
172.830
171.297
162.663
156.687
153.051
153.003
136.820
136.798
129.613
129.193
129.146
128.852
128.813
127.278
127.138
120.839
118.647
111.567
68.124
61.365
61.345
56.010
55.950
54.561
54.535
54.370
45.957
37.352
14.409
ppm (f1)

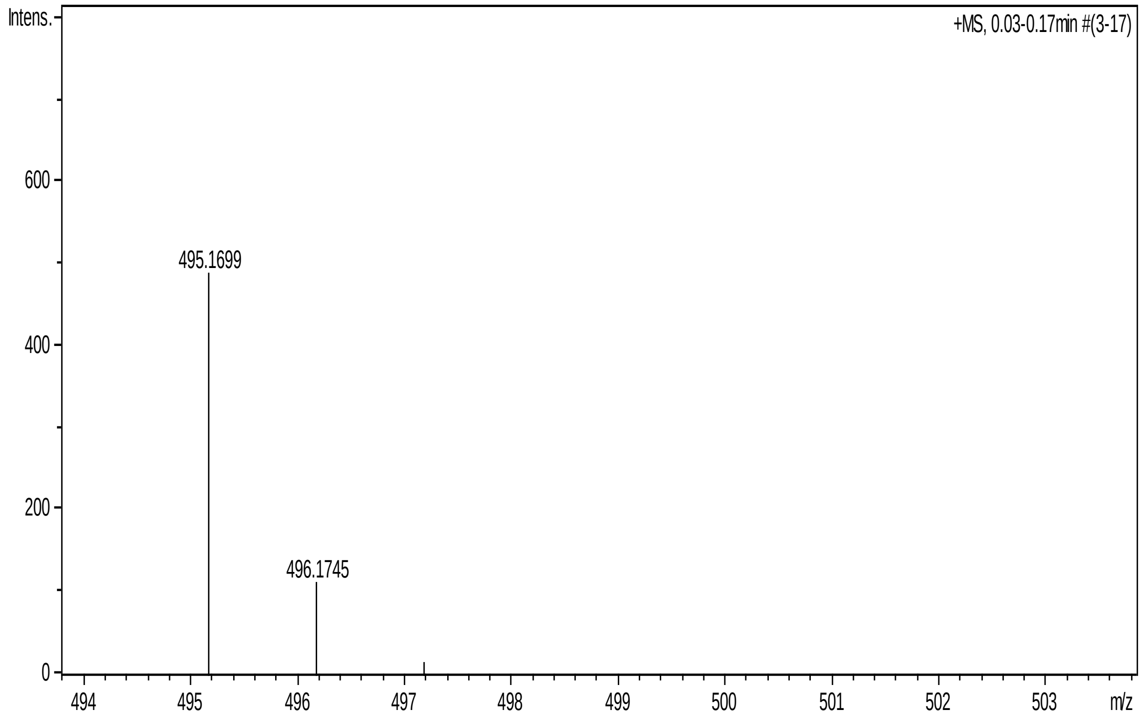
Intens.
+MS, 0.03-0.17min #(3-17)
495.1699
496.1745
m/z

ethyl ((5-amino-4-cyano-3-(p-tolyl)-2,3-dihydrothiophene-2-carbonyl)carbamoyl)phenylalaninate (2j): yellow solid, 55%, m.p. 180-182℃; ^{1}H NMR (400 MHz, DMSO-d_6) δ: 10.56 (s, 1H, NH), 8.49 (s, 1H, NH), 7.33-7.30 (m, 2H, ArH), 7.27-7.25 (m, 1H, ArH), 7.23-7.17 (m, 8H, ArH, NH_2), 4.58-4.53 (m, 1H, CH), 4.50-4.47 (m, 1H, CH), 4.13-4.06 (m, 3H, CH), 3.12-3.06 (m, 2H, CH), 2.88 (s, 3H, CH_3), 1.15 (t, J = 6.8 Hz, 3H, CH_3); *dr*-isomer: 2.89 (s, 3H, CH_3), 1.14 (t, J = 6.8 Hz, 3H, CH_3). *dr*-ratio = 0.52:0.48. ^{13}C NMR (100 MHz, DMSO-d6) δ: 172.2, 171.2, 171.1, 161.8, 161.7, 152.9, 152.9, 138.8, 138.7, 137.1, 136.7, 136.6, 129.7, 129.6, 128.8, 127.4, 127.2, 118.6, 70.9, 70.8, 61.3, 55.7, 55.6, 54.3, 54.2, 51.6, 51.5, 37.4, 37.3, 21.0, 14.3; IR (KBr) υ: 3439, 3313, 3221, 3149, 3031, 2972, 2178, 1750, 1695, 1628, 1583, 1539, 1494, 1348, 1246, 1184, 1119, 1034, 975, 887 cm^{-1}; MS (*m*/*z*): HRMS (ESI) Calcd. for $C_{25}H_{26}N_4NaO_4S$ ($[M+Na]^+$): 501.1567, found: 501.1569.

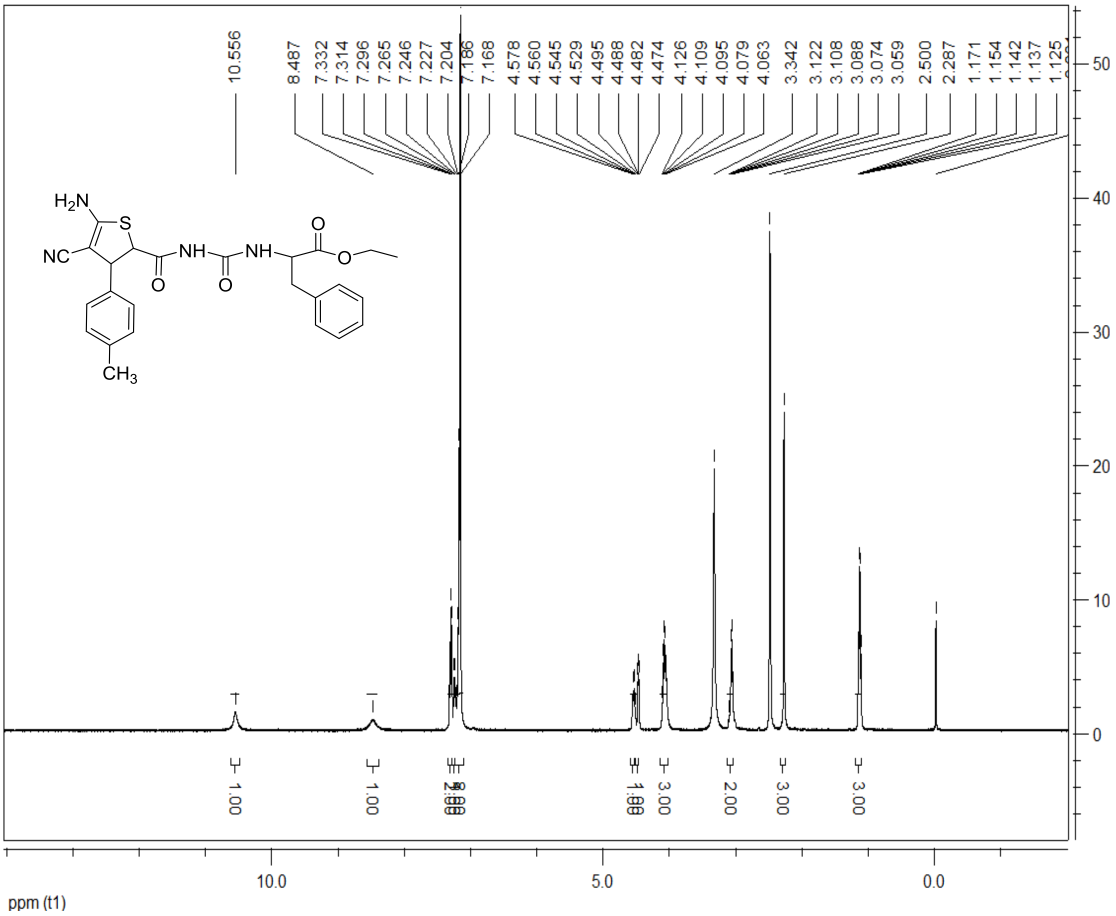

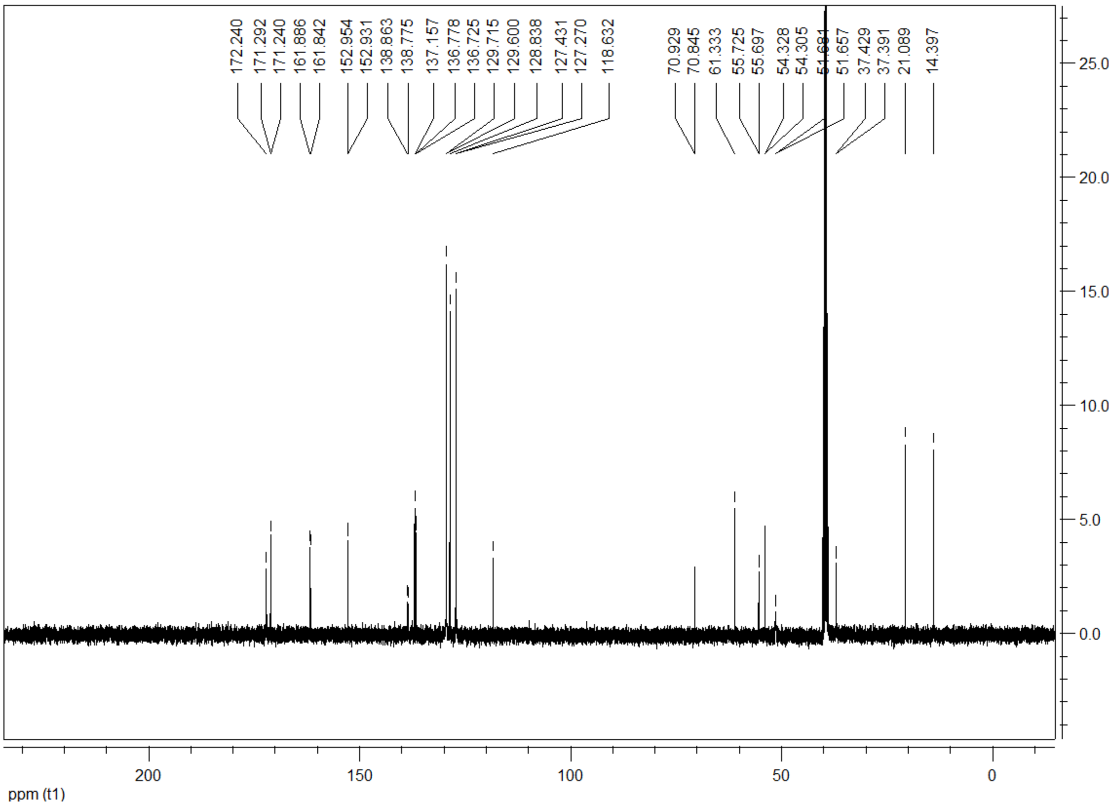
172.240
171.292
171.240
161.886
161.842
152.954
152.931
138.863
138.775
137.157
136.778
136.725
129.715
129.600
128.838
127.431
127.270
118.632
70.929
70.845
61.333
55.725
55.697
54.328
54.305
51.681
51.657
37.429
37.391
21.089
14.397
25.0
20.0
15.0
10.0
5.0
0.0
200
150
100
50
0
ppm (t1)

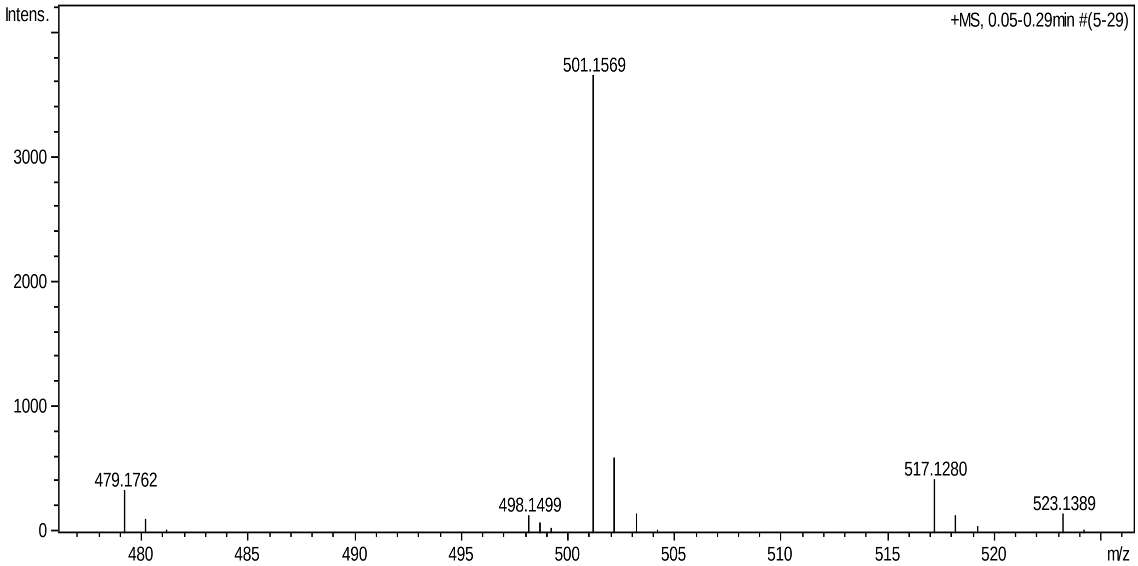
Intens.
+MS, 0.05-0.29min #(5-29)
501.1569
3000
2000
1000
0
479.1762
498.1499
517.1280
523.1389
480
485
490
495
500
505
510
515
520
m/z

Ethyl ((5-amino-3-(4-chlorophenyl)-4-cyano-2,3-dihydrothiophene-2-carbonyl)carbamoyl)phenylalaninate (2k): yellow solid, 53%, m.p. 180-182℃; ^{1}H NMR (400 MHz, DMSO-d_6) δ: 10.56 (s, 1H, NH), 8.48 (s, 1H, NH), 7.44 (d, J = 7.6 Hz, 2H, ArH), 7.33-7.25 (m, 7H, ArH, NH_2), 7.21-7.17 (m, 2H, ArH), 4.57-4.52 (m, 2H, CH), 4.13-4.06 (m, 3H, CH), 3.09-3.08 (m, 2H, CH), 1.15 (t, J = 7.2 Hz, 3H, CH_3); *dr*-isomer: 1.14 (t, J = 7.2 Hz, 3H, CH_3). *dr*-ratio = 0.53:0.47. ^{13}C NMR (100 MHz, DMSO-d6) δ: 172.1, 172.0, 171.3, 171.2, 162.3, 162.2, 153.0, 152.9, 140.9, 140.8, 136.7, 136.6, 132.5, 129.6, 129.5, 129.4, 129.1, 128.8, 128.7, 127.2, 118.5, 109.9, 70.4, 70.3, 61.3, 61.2, 55.4, 55.3, 54.2, 54.1, 51.1, 51.0, 37.4, 37.3, 14.4; IR (KBr) υ: 3442, 3318, 3220, 3146, 2976, 2180, 1750, 1693, 1627, 1581, 1536, 1494, 1349, 1304, 1247, 1185, 1092, 1027, 976, 825 cm^{-1}; MS (m/z): HRMS (ESI) Calcd. for $C_{24}H_{23}ClN_4O_4S$ ($[M+H]^+$): 521.1129, found: 521.1017.

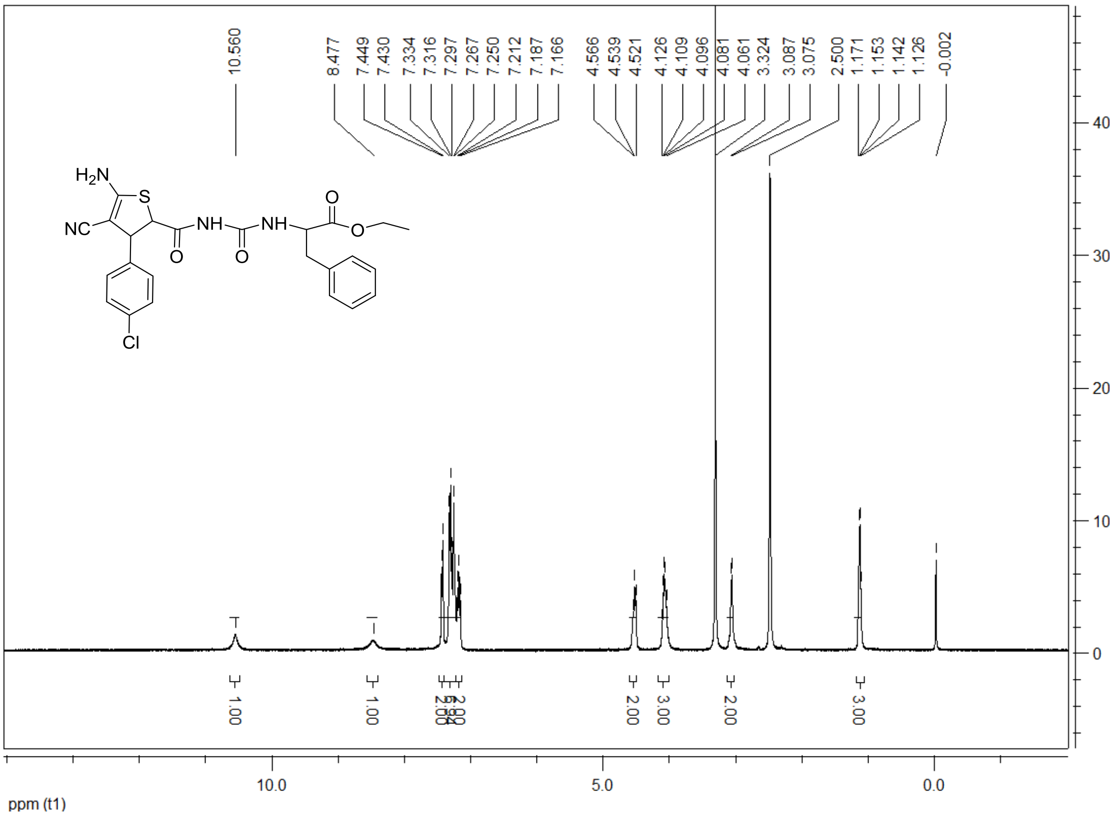

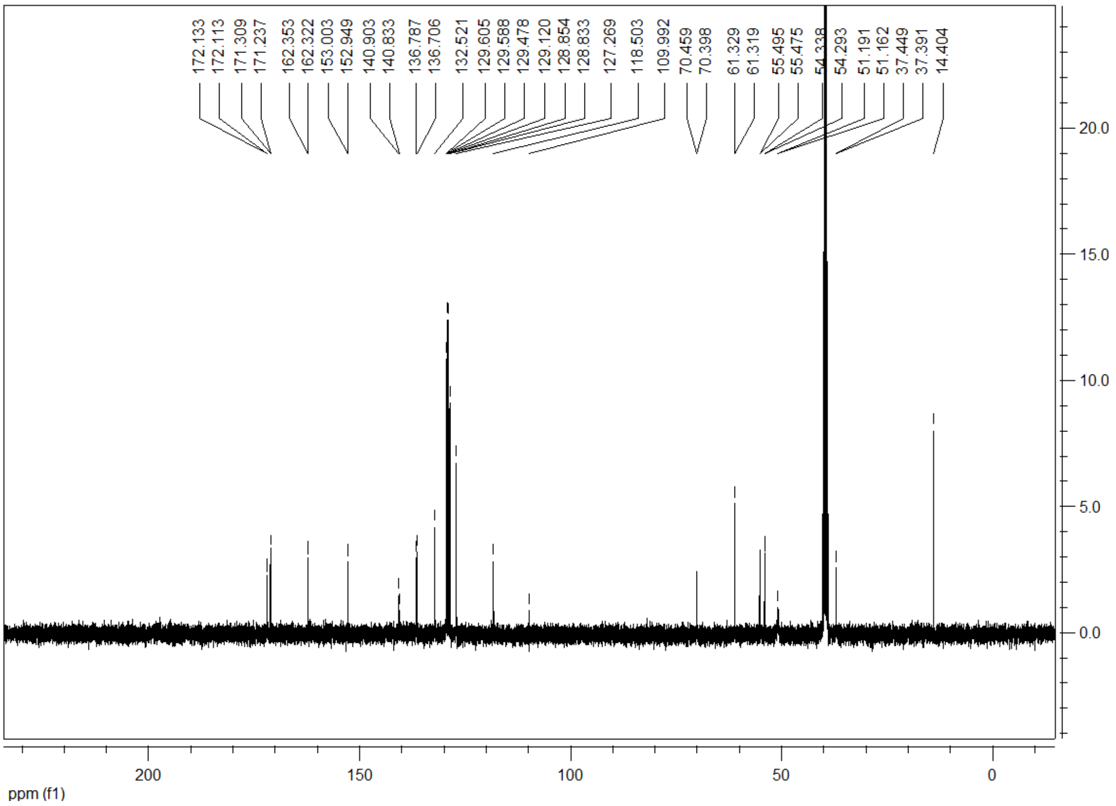
172.133
172.113
171.309
171.237
162.353
162.322
153.003
152.949
140.903
140.833
136.787
136.706
132.521
129.605
129.588
129.478
129.120
128.854
128.833
127.269
118.503
109.992
70.459
70.398
61.329
61.319
55.495
55.475
54.338
54.293
51.191
51.162
37.449
37.391
14.404
ppm (f1)

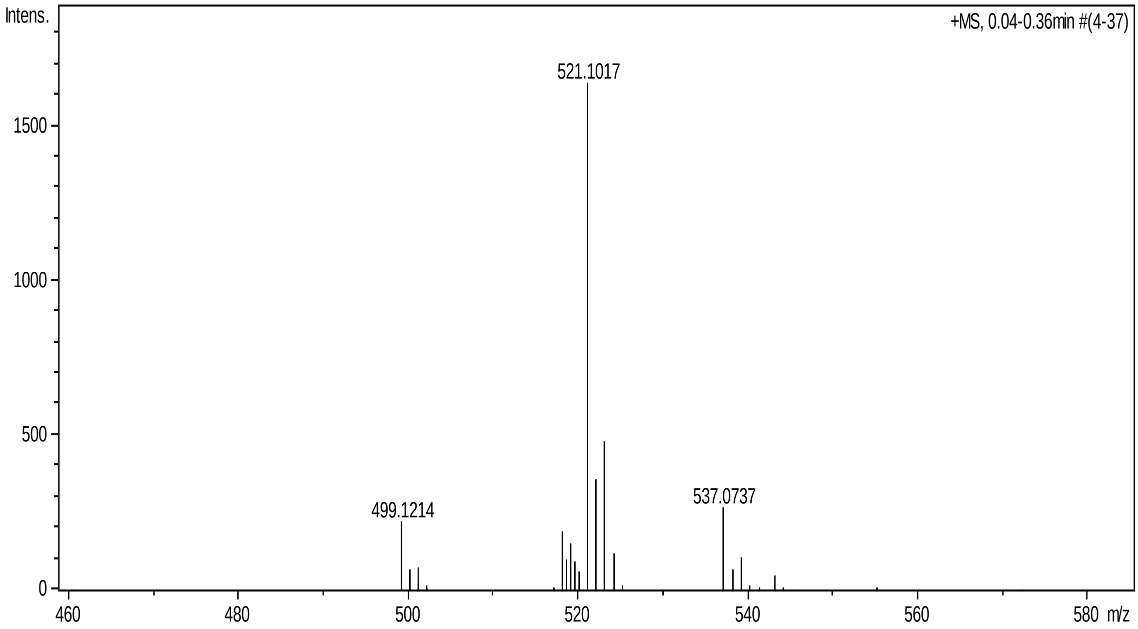
Intens.
+MS, 0.04-0.36min #(4-37)
521.1017
499.1214
537.0737
m/z

Ethyl ((5-amino-3-(4-bromophenyl)-4-cyano-2,3-dihydrothiophene-2-carbonyl)carbamoyl)phenylalaninate (2l): yellow solid, 55%, m.p. 174-176℃; ^{1}H NMR (400 MHz, DMSO-d_6) δ: 10.55 (s, 1H, NH), 8.47 (s, 1H, NH), 7.58-7.56 (m, 2H, ArH), 7.33-7.30 (m, 2H, ArH), 7.29-7.23 (m, 5H, ArH, NH_2), 7.21-7.17 (m, 2H, ArH), 4.58-4.55 (m, 1H, CH), 4.52-4.50 (m, 1H, CH), 4.13-4.07 (m, 3H, CH), 3.11-3.06 (m, 2H, CH), 1.15 (t, J = 7.2 Hz, 3H, CH_3); *dr*-isomer: 1.14 (t, J = 7.2 Hz, 3H, CH_3). *dr*-ratio = 0.53:0.47. ^{13}C NMR (100 MHz, DMSO-d6) δ: 172.1, 172.0, 171.3, 171.2, 162.3, 162.2, 153.0, 152.9, 136.7, 136.6, 132.0, 131.9, 132.0, 129.8, 129.7, 129.6, 128.8, 127.2, 121.0, 118.5, 70.3, 70.2, 61.3, 61.2, 61.3, 55.4, 55.3, 55.2, 54.3, 54.2, 54.1, 51.1, 37.4, 37.3, 14.4, 14.3; IR (KBr) υ: 3444, 3318, 3219, 3145, 2972, 2180, 1749, 1694, 1626, 1580, 1536, 1493, 1349, 1246, 1185, 1019, 976, 823 cm^{-1}; MS (m/z): HRMS (ESI) Calcd. for $C_{24}H_{23}BrN_4O_4SNa$ ($[M+Na]^+$): 565.0623, found: 565.0529.

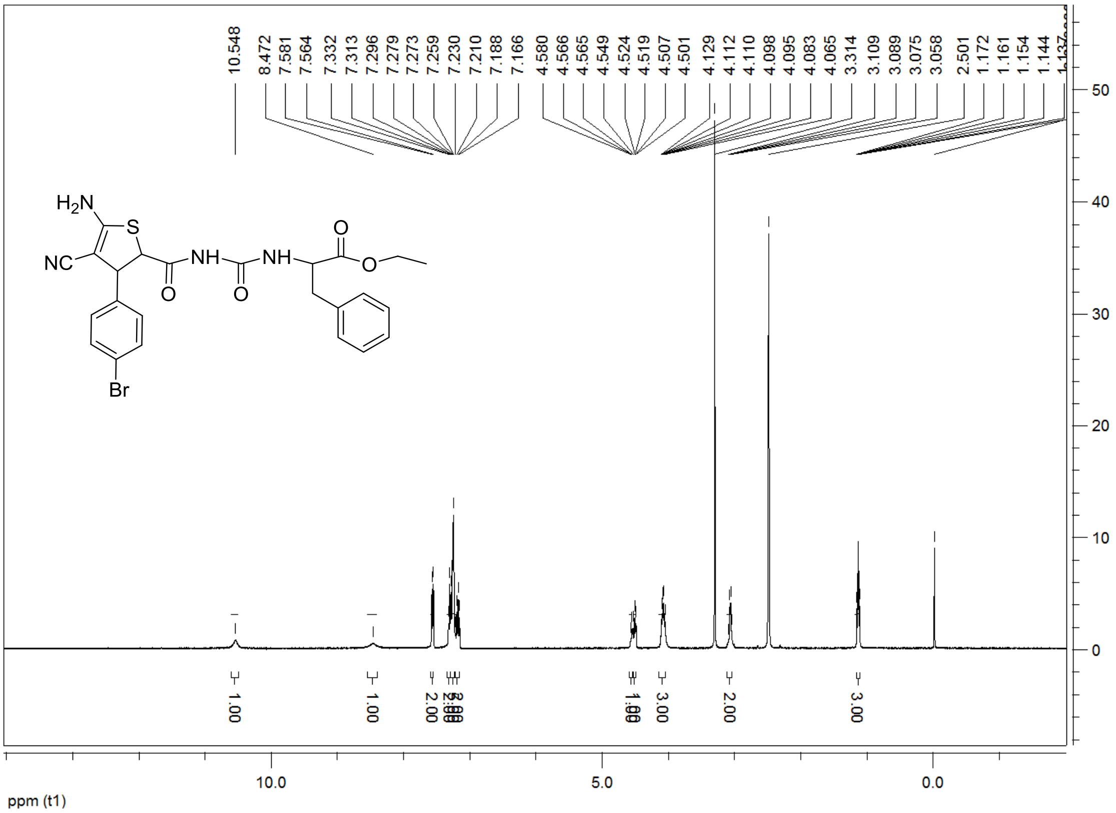

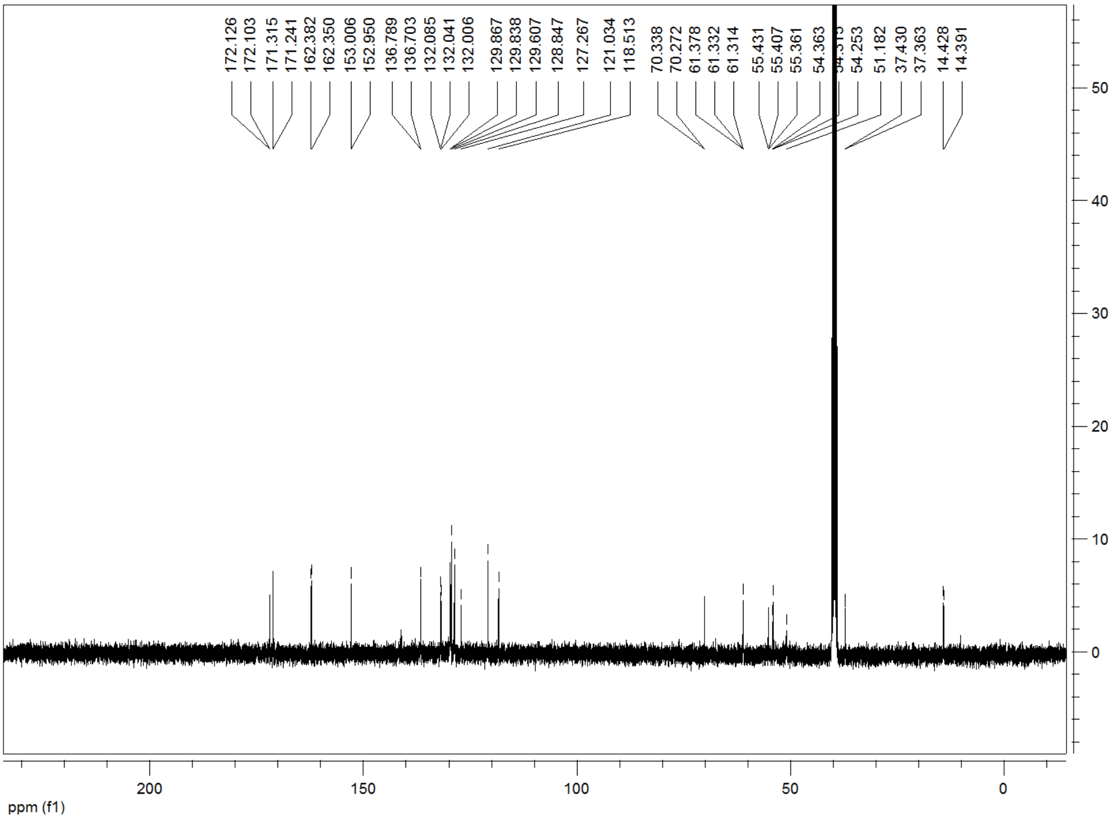
172.126
172.103
171.315
171.241
162.382
162.350
153.006
152.950
136.789
136.703
132.085
132.041
132.006
129.867
129.838
129.607
128.847
127.267
121.034
118.513
70.338
70.272
61.378
61.332
61.314
55.431
55.407
55.361
54.363
54.253
51.182
37.430
37.363
14.428
14.391
50
40
30
20
10
0
200
150
100
50
0
ppm (f1)

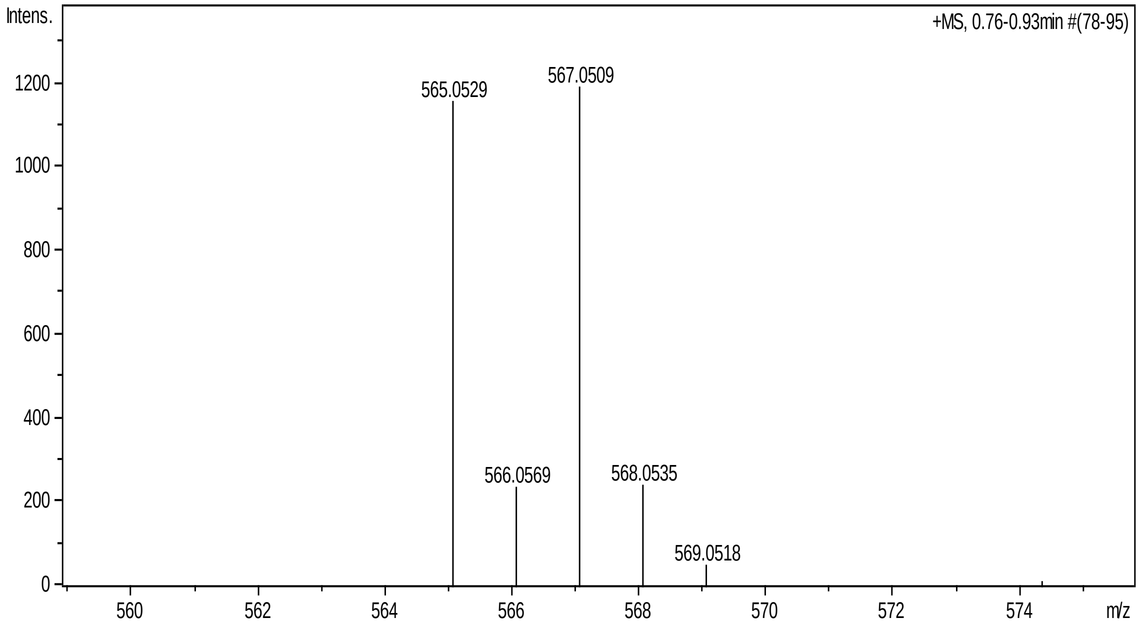
Intens.
+MS, 0.76-0.93min #(78-95)
565.0529
567.0509
566.0569
568.0535
569.0518
1200
1000
800
600
400
200
0
560
562
564
566
568
570
572
574
m/z

Ethyl 2-(3-(5-amino-4-cyano-3-(4-methoxyphenyl)-2,3-dihydrothiophene-2-carbonyl)ureido)-4-phenylbutanoate (2m): white solid, 67%, m.p. 218-220℃; ^{1}H NMR (400 MHz, DMSO-d_6) δ: 10.59 (s, 1H, NH), 8.58 (s, 1H, NH), 7.31-7.27 (m, 2H, ArH,), 7.24-7.15 (m, 7H, ArH, NH_2), 6.93 (d, J = 8.8 Hz, 2H, ArH), 4.54-4.53 (m, 1H, CH), 4.30-4.28 (m, 1H, CH), 4.13-4.10 (m, 3H, CH),, 3.75 (s, 3H, OCH_3), 2.65-2.60 (m, 2H, CH), 2.10-2.03 (m, 2H, CH), 1.20 (t, J = 7.2 Hz, 3H, CH_3); ; *dr*-isomer: 3.74 (s, 3H, OCH_3), 1.19 (t, J = 7.2 Hz, 3H, CH_3). *dr*-ratio = 0.54:0.46. ^{13}C NMR (100 MHz, DMSO-d6) δ: 172.4, 172.3, 171.8, 171.7, 161.7, 159.1, 153.1, 153.0, 140.9, 140.8, 133.7, 133.6, 128.8, 128.7, 128.6, 126.4, 118.6, 114.5, 71.2, 71.2, 61.3, 61.2, 55.9, 55.8, 55.5, 55.4, 52.4, 51.4, 51.3, 33.4, 31.5, 31.4, 14.4; IR (KBr) υ: 3423, 3313, 3224, 2925, 2855, 2182, 1698, 1632, 1581, 1543, 1505, 1348, 1306, 1249, 1181, 1029, 976, 830 cm^{-1}; MS (m/z): HRMS (ESI) Calcd. for $C_{26}H_{28}N_4O_5S$ ($[M+Na]^+$): 531.1780, found: 531.1680.

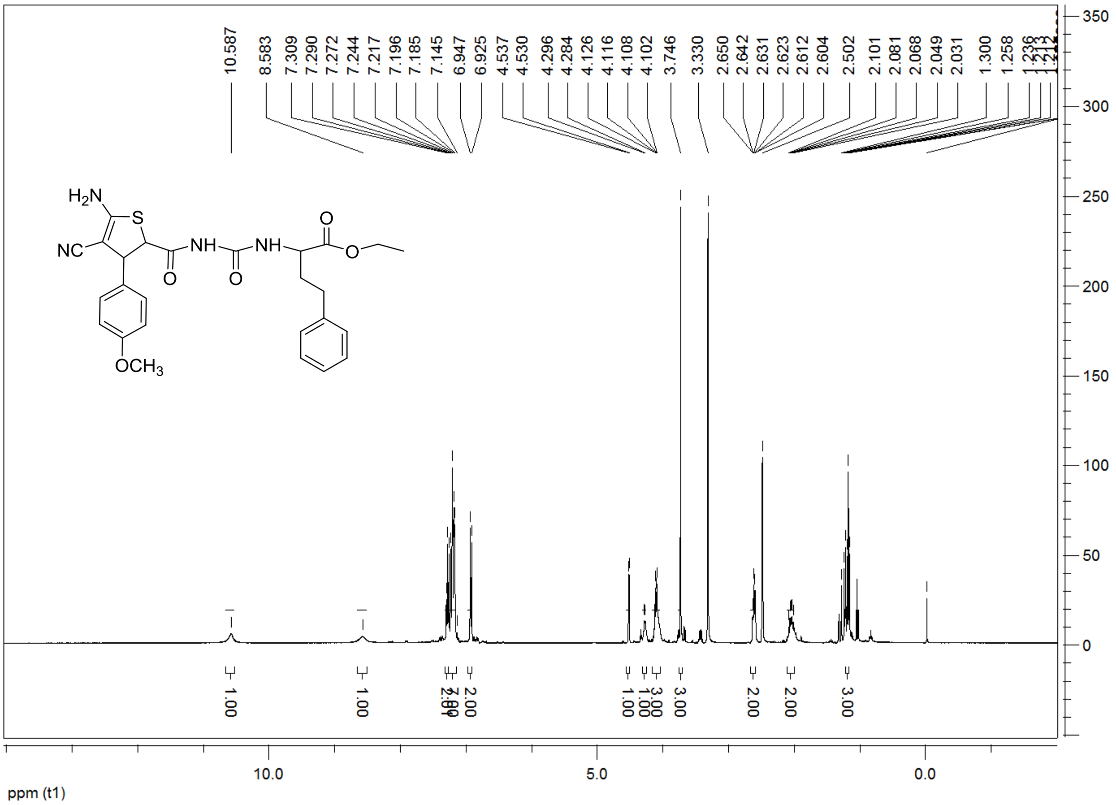

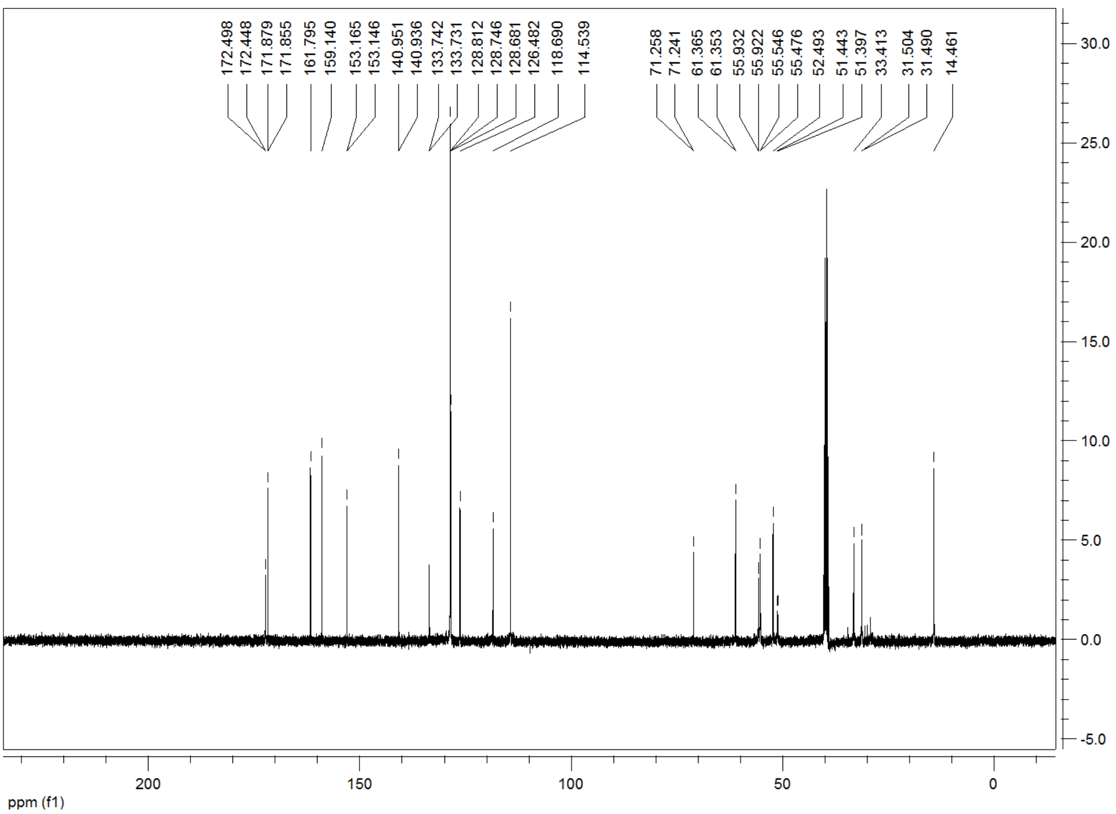
172.498
172.448
171.879
171.855
161.795
159.140
153.165
153.146
140.951
140.936
133.742
133.731
128.812
128.746
128.681
126.482
118.690
114.539
71.258
71.241
61.365
61.353
55.932
55.922
55.546
55.476
52.493
51.443
51.397
33.413
31.504
31.490
14.461
ppm (f1)

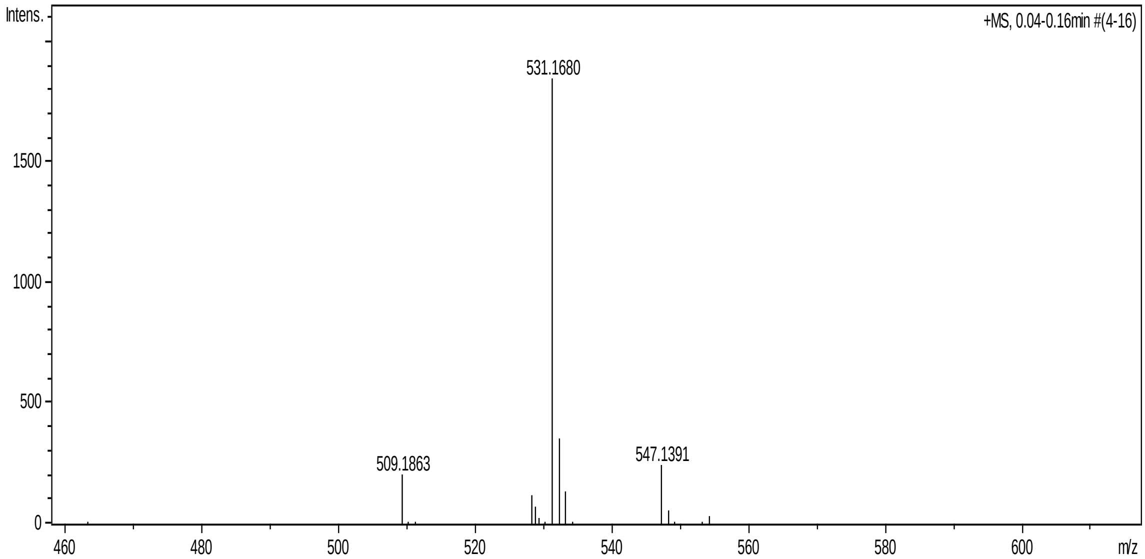
Intens.
+MS, 0.04-0.16min #(4-16)
531.1680
509.1863
547.1391
m/z

Ethyl ((5-amino-4-cyano-3-(4-methoxyphenyl)-2,3-dihydrothiophene-2-carbonyl)carbamoyl)tyrosinate (2n): white solid, 57%, m.p. 171-173℃; ^{1}H NMR (400 MHz, DMSO-d_6) δ: 10.55 (s, 1H, NH), 9.29 (d, J = 6.0 Hz, 1H, OH), 8.45 (s, 1H, NH), 7.27-7.17 (m, 2H, ArH, NH_2), 6.98-6.92 (m, 4H, ArH), 6.70-6.68 (m, 2H, ArH), 4.49-4.45 (m, 2H, CH), 4.12-4.06 (m, 3H, CH), 3.75 (s, 3H, OCH_3), 2.96 (m, 2H, CH), 1.17 (t, J = 6.8 Hz, 3H, CH_3); dr-isomer δ: 3.74 (s, 3H, OCH_3), 1.15 (t, J = 7.2 Hz, 3H, CH_3). *dr*-ratio = 0.55:0.45. ^{13}C NMR (100 MHz, DMSO-d6) δ: 172.2, 172.1, 171.4, 171.3, 161.7, 161.6, 159.1, 156.6, 152.9, 133.7, 133.6, 133.5, 130.5, 128.6, 126.6, 126.5, 118.6, 115.6, 114.5, 71.1, 71.0, 61.2, 61.1, 55.8, 55.7, 55.5, 54.5, 51.5, 51.4, 51.3, 36.7, 36.6, 14.4; IR (KBr) υ: 3442, 3310, 3221, 3145, 2969, 2840, 2178, 1747, 1692, 1623, 1580, 1544, 1509, 1349, 1303, 1247, 1180, 1113, 1033, 976, 826 cm^{-1}; MS (m/z): HRMS (ESI) Calcd. for $C_{25}H_{26}N_4O_6S$ ($[M+Na]^+$): 533.1573, found: 533.1474.

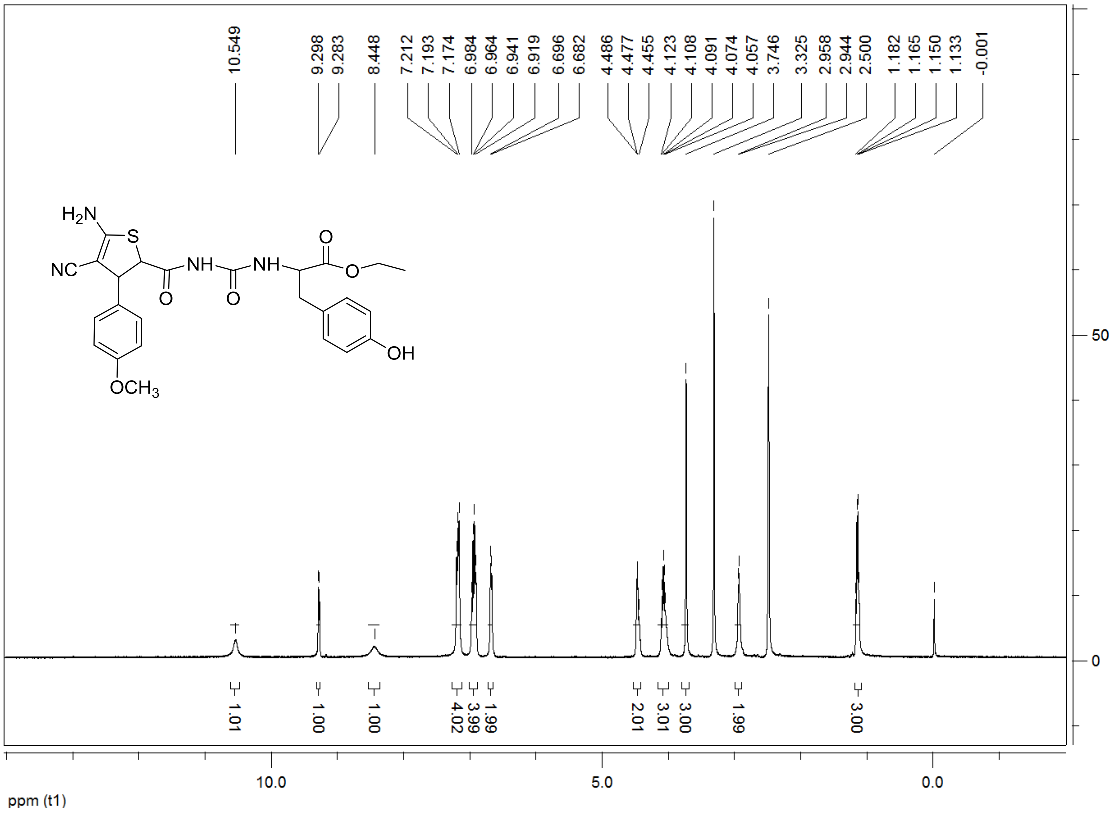

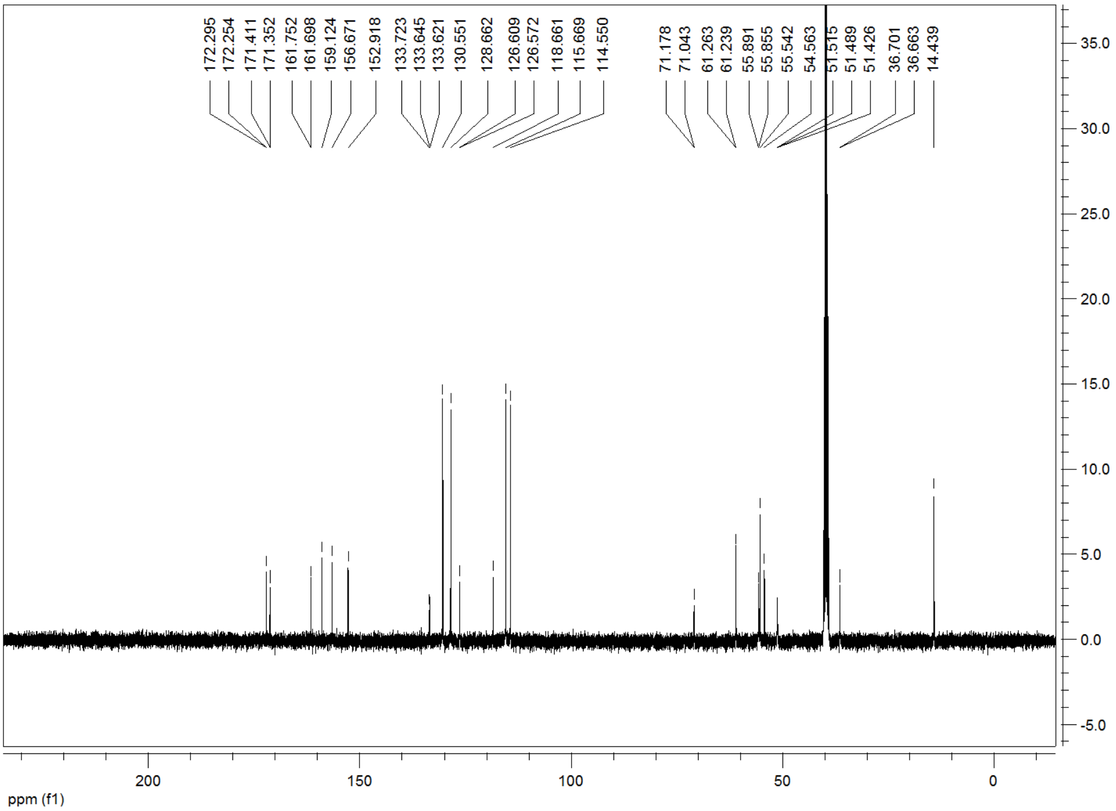
172.295
172.254
171.411
171.352
161.752
161.698
159.124
156.671
152.918
133.723
133.645
133.621
130.551
128.662
126.609
126.572
118.661
115.669
114.550
71.178
71.043
61.263
61.239
55.891
55.855
55.542
54.563
51.515
51.489
51.426
36.701
36.663
14.439
ppm (f1)

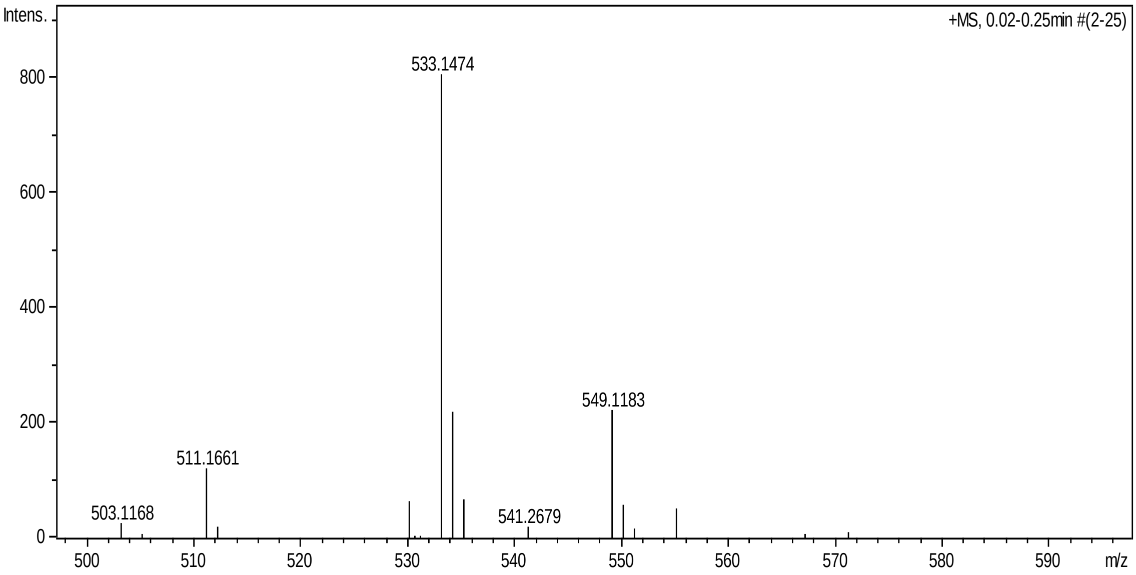
Intens.
+MS, 0.02-0.25min #(2-25)
533.1474
549.1183
511.1661
503.1168
541.2679
m/z

Ethyl

((5-amino-4-cyano-3-(4-methoxyphenyl)thiophene-2-carbonyl)carbamoyl)phenylalaninate

(3a): yellow solid, 62%, m.p. 177-179℃; ^{1}H NMR (400 MHz, DMSO-*d*$_6$) δ: 8.49 (d, *J* = 7.6 Hz, 1H, NH), 8.10 (d, *J* = 8.0 Hz, 3H, NH, NH_2), 7.35 (d, *J* = 8.4 Hz, 2H, ArH), 7.31-7.21 (m, 3H, ArH), 7.13 (d, *J* = 8.8 Hz, 2H, ArH), 7.08 (d, *J* = 8.8 Hz, 2H, ArH), 4.51-4.46 (m, 1H, CH), 4.06 (q, *J* = 7.2 Hz, 2H, CH), 3.82 (s, 3H, OCH_3), 3.07-2.97 (m, 2H, CH), 1.13 (t, *J* = 7.2 Hz, 3H, CH_3); ^{13}C NMR (100 MHz, DMSO-d6) δ: 171.2, 167.6, 161.9, 160.6, 152.0, 146.0, 136.7, 130.8, 129.5, 128.7, 127.2, 124.4, 115.3, 114.9, 111.9, 89.5, 61.3, 55.7, 54.2, 37.4, 14.3; IR (KBr) υ: 3438, 3373, 3281, 3168, 2967, 2211, 1730, 1680, 1648, 1516, 1467, 1412, 1316, 1256, 1181, 1078, 1024, 876, 840 cm^{-1}; MS (*m/z*): HRMS (ESI) Calcd. for $C_{25}H_{24}N_4O_5S$ ($[M+Na]^+$): 515.1467, found: 515.1357.

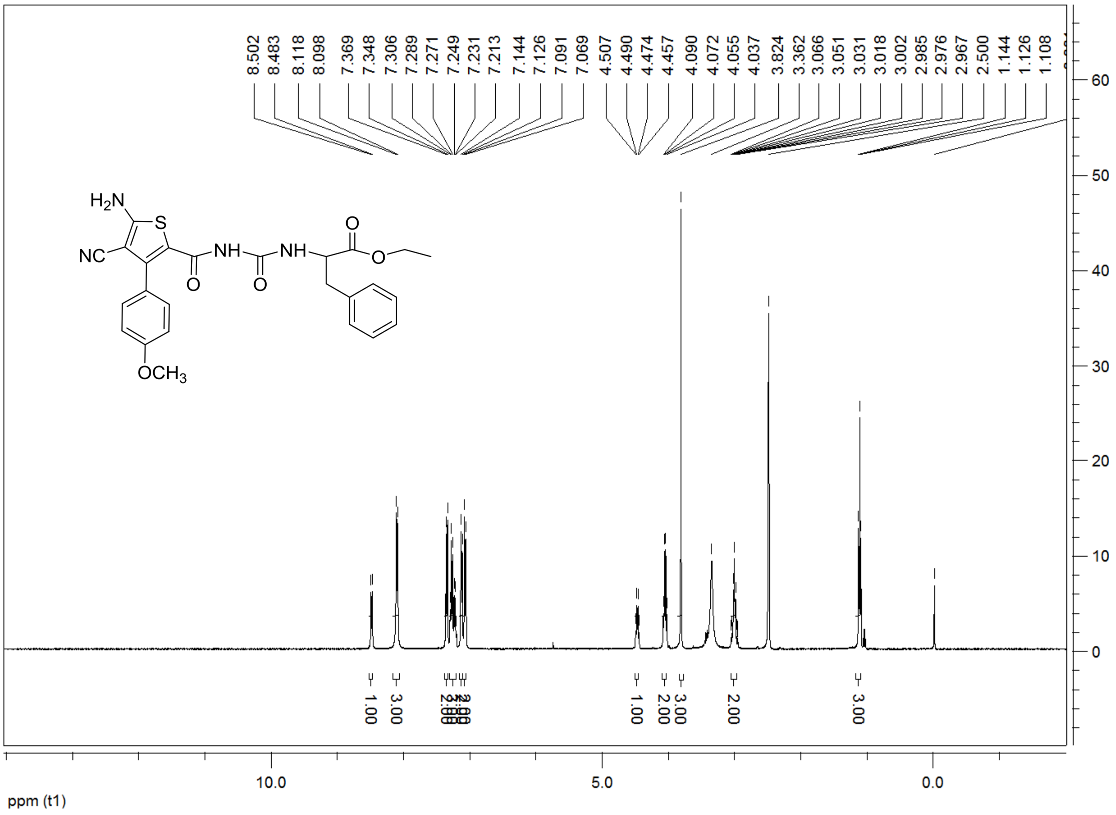

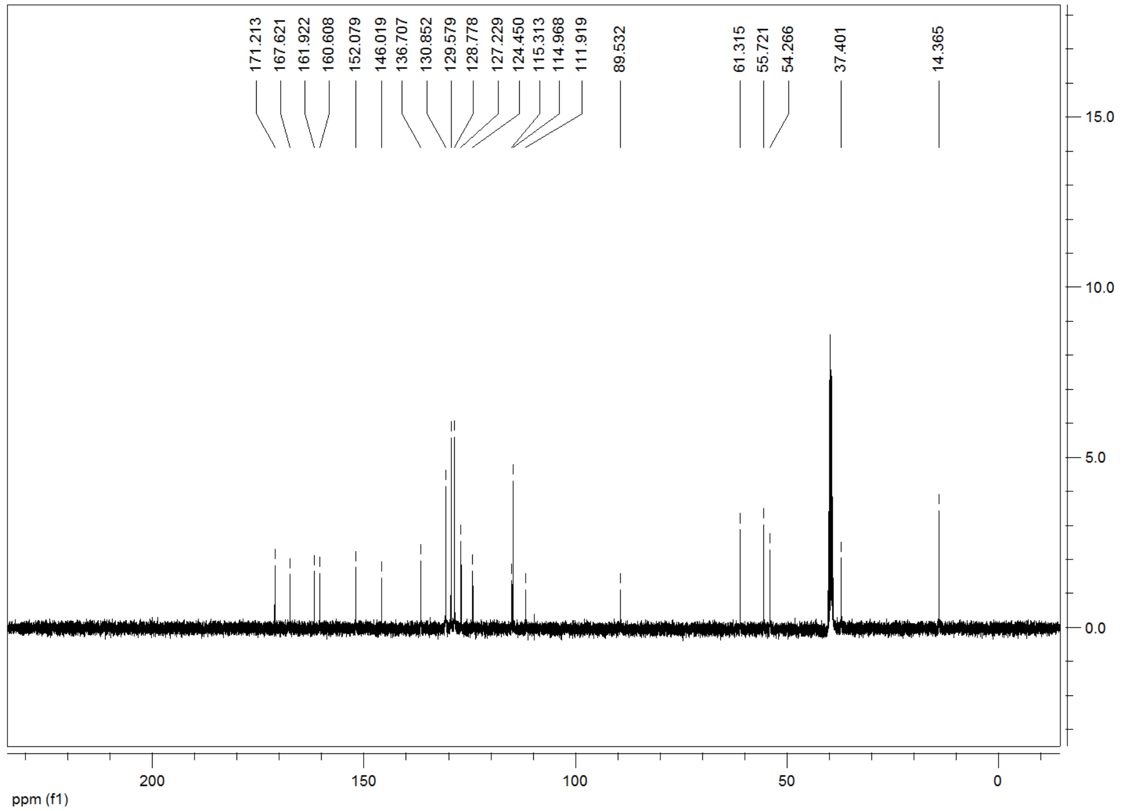

171.213
167.621
161.922
160.608
152.079
146.019
136.707
130.852
129.579
128.778
127.229
124.450
115.313
114.968
111.919
89.532
61.315
55.721
54.266
37.401
14.365
15.0
10.0
5.0
0.0
200
150
100
50
0
ppm (f1)

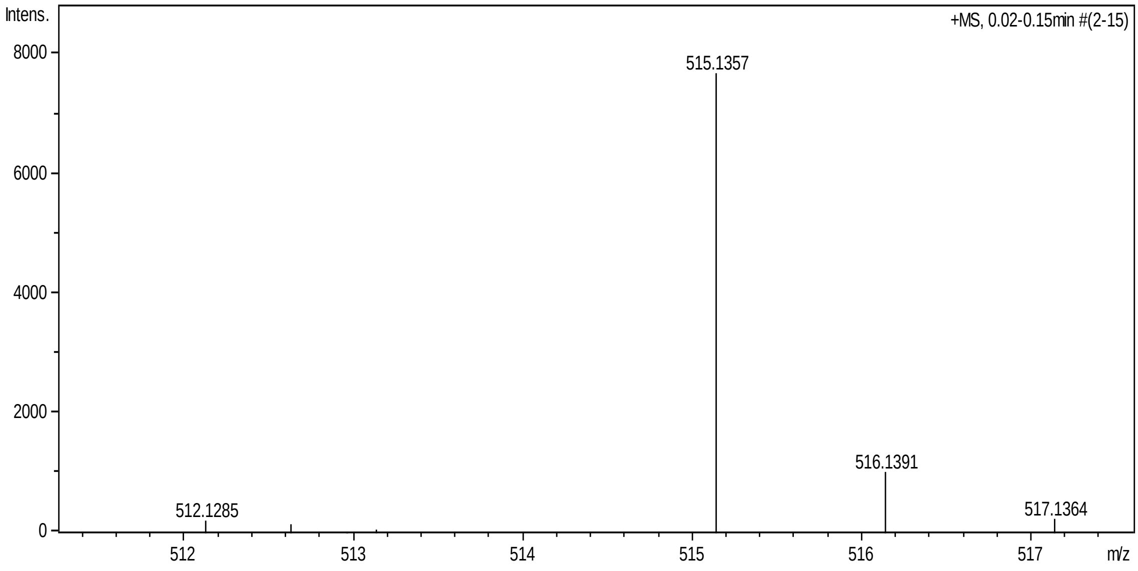

Intens.
+MS, 0.02-0.15min #(2-15)
515.1357
516.1391
512.1285
517.1364
8000
6000
4000
2000
0
512
513
514
515
516
517
m/z

ethyl ((5-amino-4-cyano-3-(3-methoxyphenyl)thiophene-2-carbonyl)carbamoyl)phenylalaninate (3b): yellow solid, 60%, m.p. 174-176℃; ^{1}H NMR (400 MHz, DMSO-d_6) δ: 8.48 (d, *J* = 7.2 Hz, 1H, NH), 8.15 (s, 3H, NH, NH_2), 7.45 (t, *J* = 7.6 Hz, 1H, ArH), 7.30-7.21 (m, 3H, ArH), 7.14-7.09 (m, 3H, ArH), 7.01-6.97 (m, 2H, ArH), 4.48 (q, *J* = 6.8 Hz, 1H, CH), 4.06 (q, *J* = 6.8 Hz, 2H, CH), 3.79 (s, 3H, OCH_3), 3.05-2.99 (m, 2H, CH), 1.13 (t, *J* = 6.8 Hz, 3H, CH_3); ^{13}C NMR (100 MHz, DMSO-d6) δ: 171.1, 167.6, 161.7, 159.8, 152.0, 145.6, 136.6, 133.8, 130.8, 129.5, 128.7, 127.2, 121.3, 115.8, 115.1, 114.8, 112.2, 89.3, 61.3, 55.7, 55.7, 54.2, 54.2, 37.3, 14.3; IR (KBr) υ: 3371, 3308, 3202, 2931, 2210, 1693, 1640, 1470, 1317, 1255, 1201, 1082, 1029, 857 cm^{-1}; MS (*m*/*z*): HRMS (ESI) Calcd. for $C_{25}H_{24}N_4O_5S$ ([M+Na]$^+$): 515.1467, found: 515.1357.

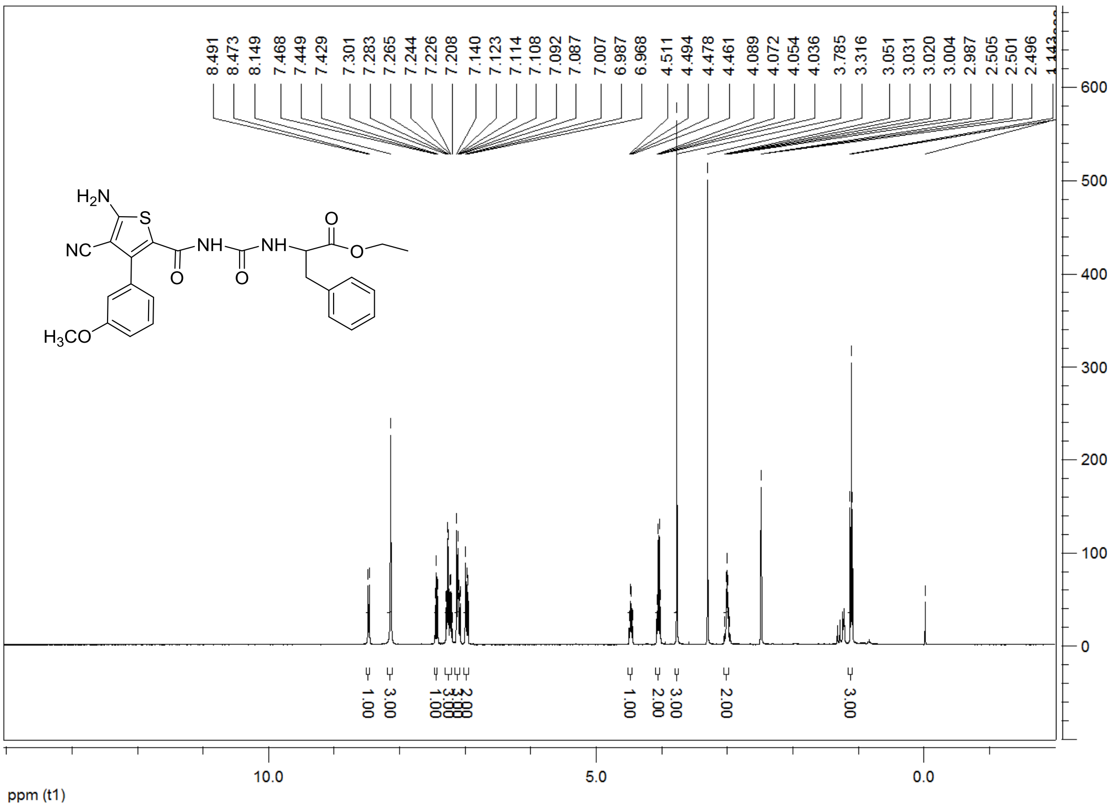

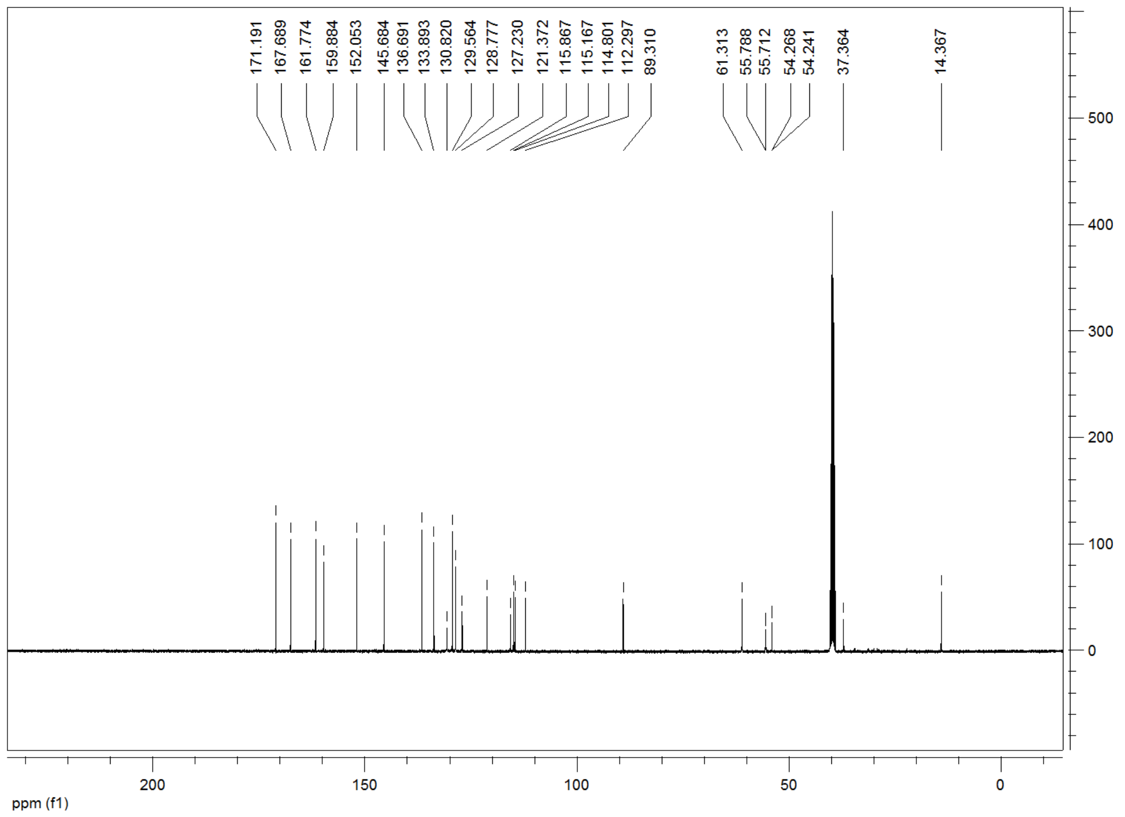
171.191
167.689
161.774
159.884
152.053
145.684
136.691
133.893
130.820
129.564
128.777
127.230
121.372
115.867
115.167
114.801
112.297
89.310
61.313
55.788
55.712
54.268
54.241
37.364
14.367
ppm (f1)

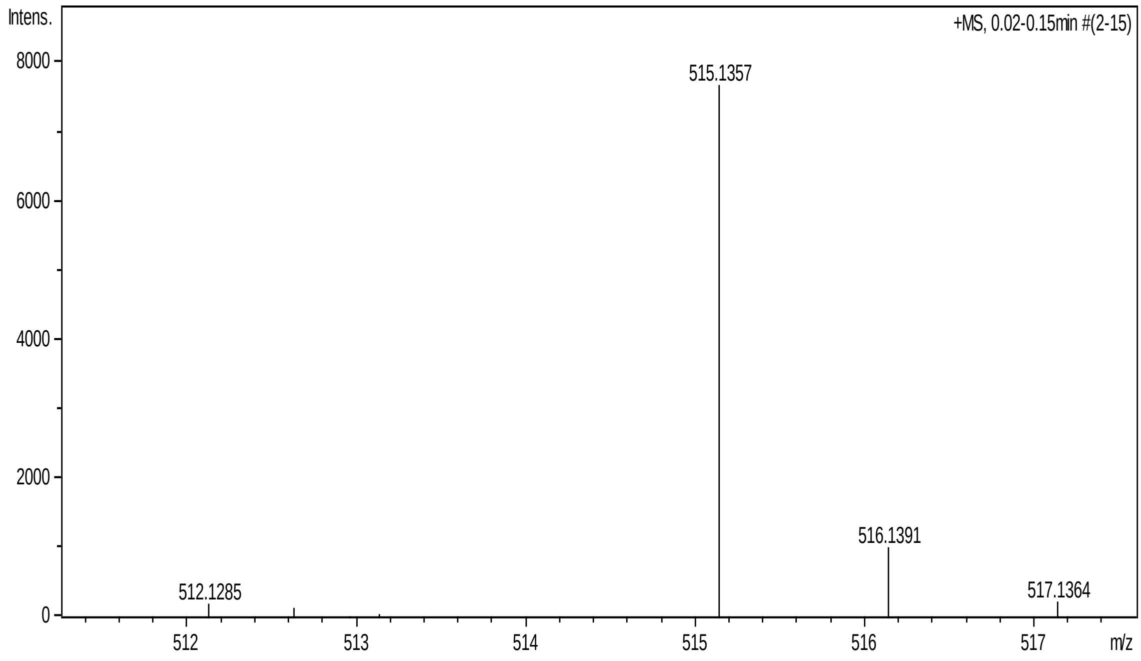
Intens.
+MS, 0.02-0.15min #(2-15)
515.1357
516.1391
512.1285
517.1364
m/z

ethyl ((5-amino-4-cyano-3-(2-methoxyphenyl)thiophene-2-carbonyl)carbamoyl)phenylalaninate (3c): yellow solid, 60%, m.p. 176-178℃; ^{1}H NMR (400 MHz, DMSO-d_6) δ: 8.48 (d, J = 7.2 Hz, 1H, NH), 8.09 (s, 2H, NH_2), 8.05 (s, 1H, NH), 7.53-7.49 (m, 1H, ArH), 7.31-7.26 (m, 3H, ArH), 7.24-7.19 (m, 2H, ArH), 7.14-7.08 (m, 3H, ArH), 4.50-4.45 (m, 1H, CH), 4.08-4.03 (m, 2H, CH), 3.72 (s, 3H, OCH_3), 3.06-2.96 (m, 2H, CH), 1.12 (t, J = 7.2 Hz, 3H, CH_3); ^{13}C NMR (100 MHz, DMSOd6) δ: 171.2, 167.3, 161.7, 156.5, 152.0, 142.3, 136.7, 132.0, 130.9, 129.5, 128.7, 127.2, 121.4, 120.8, 115.1, 112.8, 112.7, 89.6, 61.2, 56.0, 54.2, 37.3, 14.3; IR (KBr) υ: 3371, 3184, 2926, 2849, 2214, 1742, 1693, 1649, 1514, 1468, 1319, 1251, 1182, 1079, 1020, 871cm^{-1}; MS (m/z): HRMS (ESI) Calcd. for $C_{25}H_{24}N_4NaO_5S$ ($[M+Na]^+$): 515.1365, found: 515.1375.

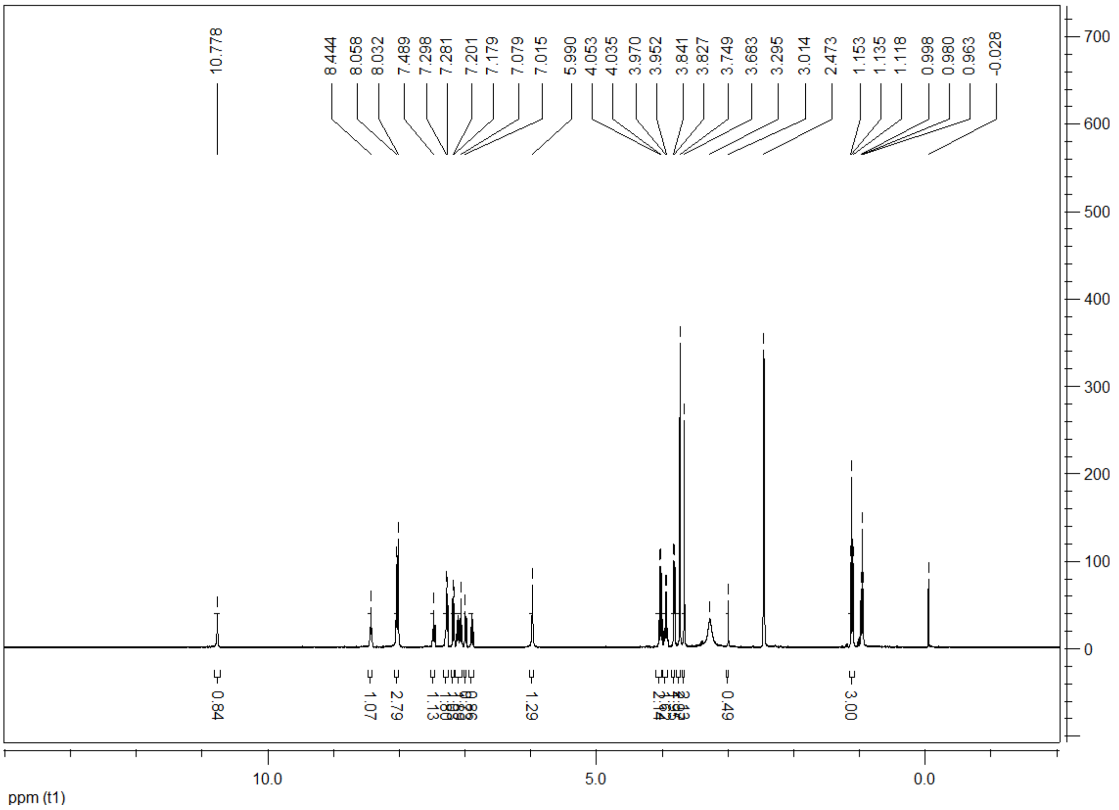

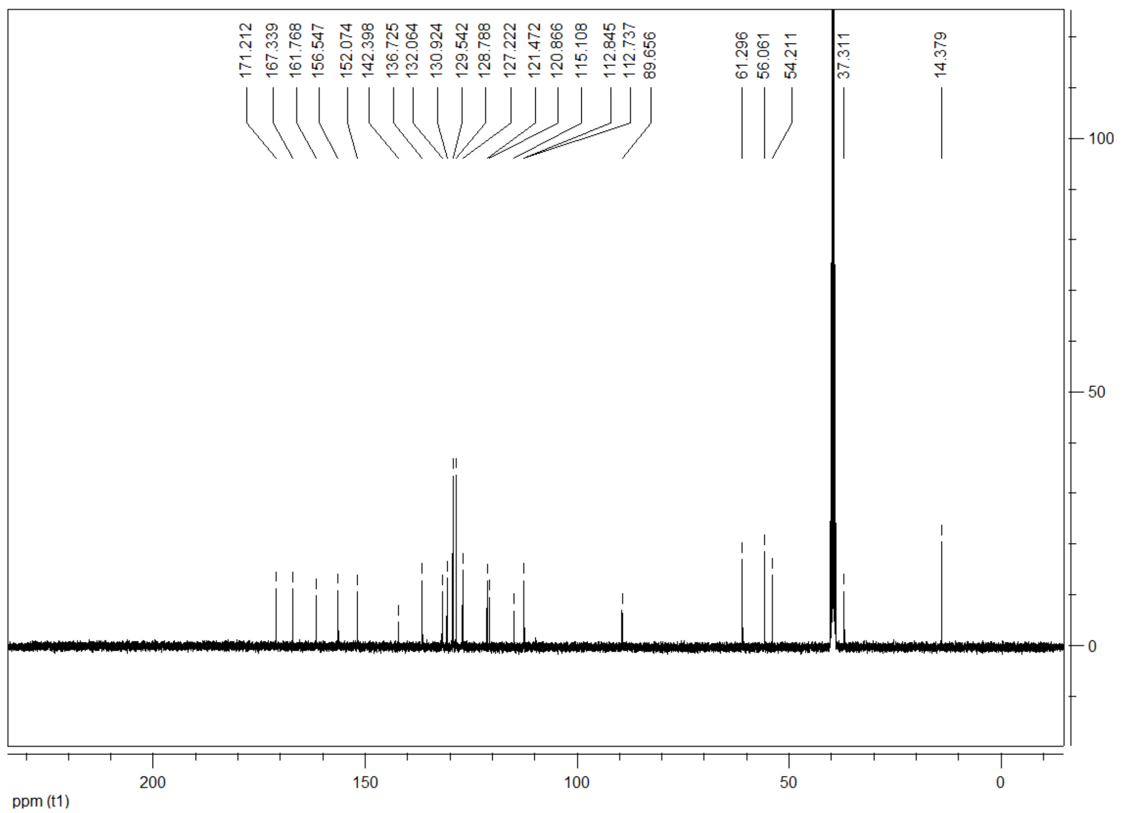
171.212
167.339
161.768
156.547
152.074
142.398
136.725
132.064
130.924
129.542
128.788
127.222
121.472
120.866
115.108
112.845
112.737
89.656
61.296
56.061
54.211
37.311
14.379
100
50
0
200
150
100
50
0
ppm (t1)

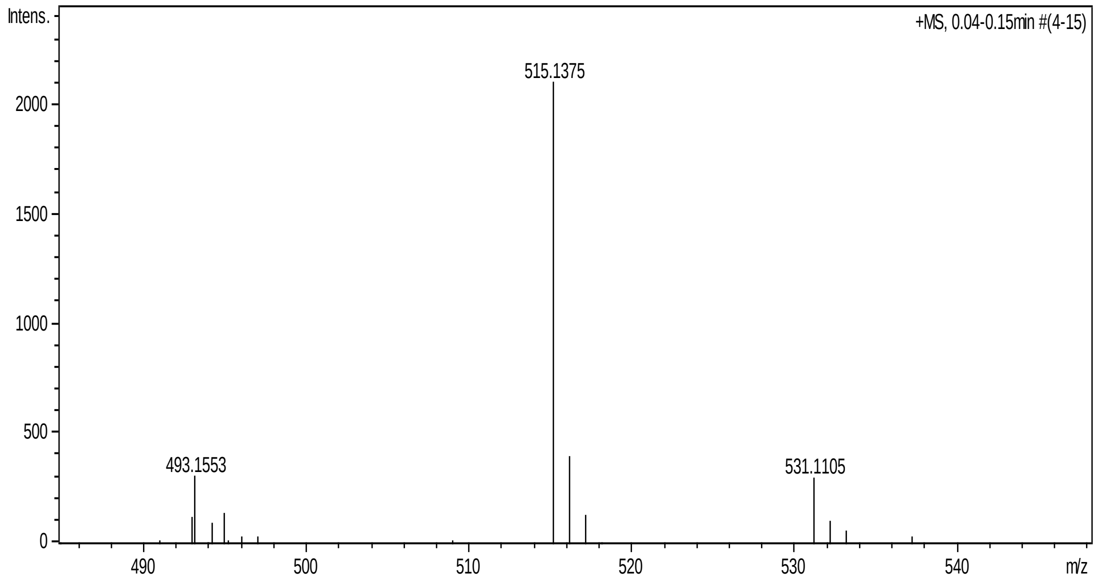
Intens.
+MS, 0.04-0.15min #(4-15)
515.1375
493.1553
531.1105
2000
1500
1000
500
0
490
500
510
520
530
540
m/z

ethyl ((5-amino-4-cyano-3-(p-tolyl)thiophene-2-carbonyl)carbamoyl)phenylalaninate (3d): yellow solid, 55%, m.p. 176-178℃; ^{1}H NMR (400 MHz, DMSO-d_6) δ: 8.49 (d, J = 7.2 Hz, 1H, NH), 8.14 (d, J = 7.6 Hz, 3H, NH NH_2), 7.35-7.21 (m, 7H, ArH), 7.13 (d, J = 7.2 Hz, 2H,ArH), 4.48 (q, J = 6.8 Hz, 1H, CH), 4.06 (q, J = 6.8 Hz, 2H, CH), 3.05- (s, 3H, OCH_3), 3.05-2.98 (m, 2H, CH), 2.38 (s, 3H, CH_3), 1.13 (t, J = 6.8 Hz, 3H, CH_3); ^{13}C NMR (100 MHz, DMSOd6) δ: 171.2, 167.6, 161.8, 152.0, 146.1, 139.7, 136.6, 130.1, 129.6, 129.5, 129.2, 128.7, 127.2, 115.2, 112.0, 89.4, 61.3, 54.2, 37.3, 21.3, 14.3; IR (KBr) υ: 3447, 3378, 3273, 3170, 2991, 2211, 1731, 1681, 1651, 1516, 1467, 1407, 1348, 1317, 1263, 1210, 1180, 1077, 1018, 881, 825cm^{-1}; MS (m/z): HRMS (ESI) Calcd. for $C_{25}H_{24}N_4O_4S$ ($[M+Na]^+$): 499.1518, found: 499.1412.

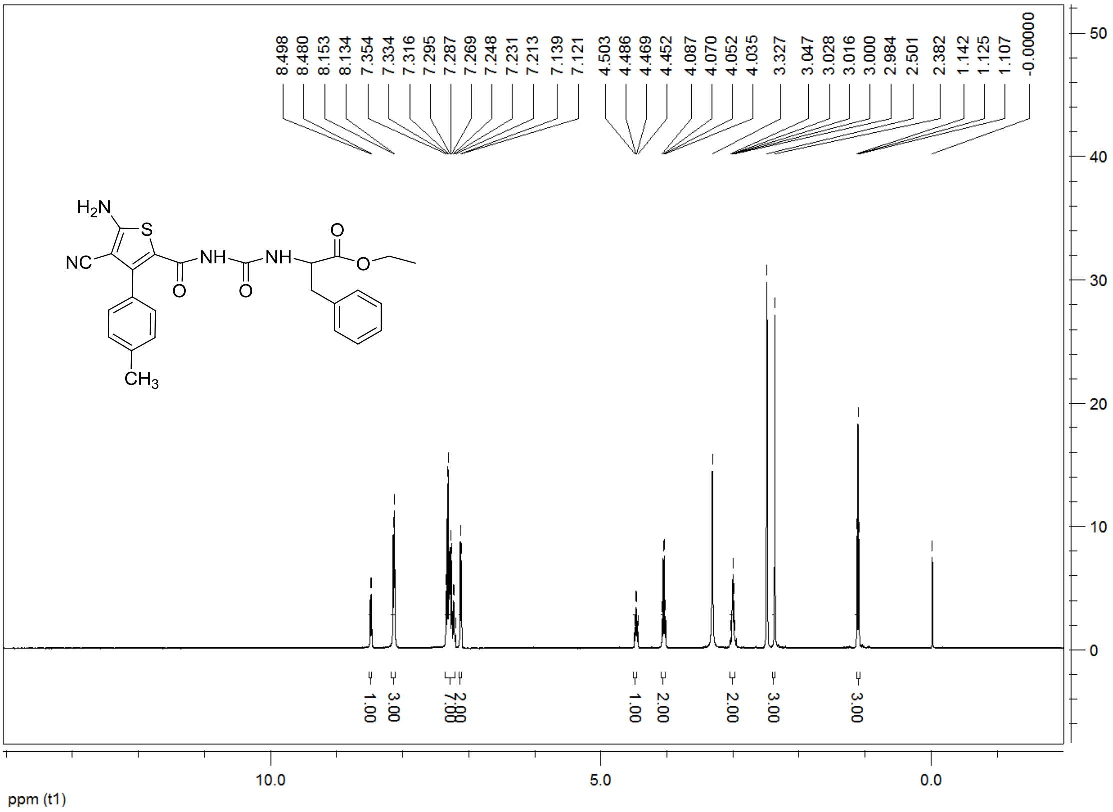

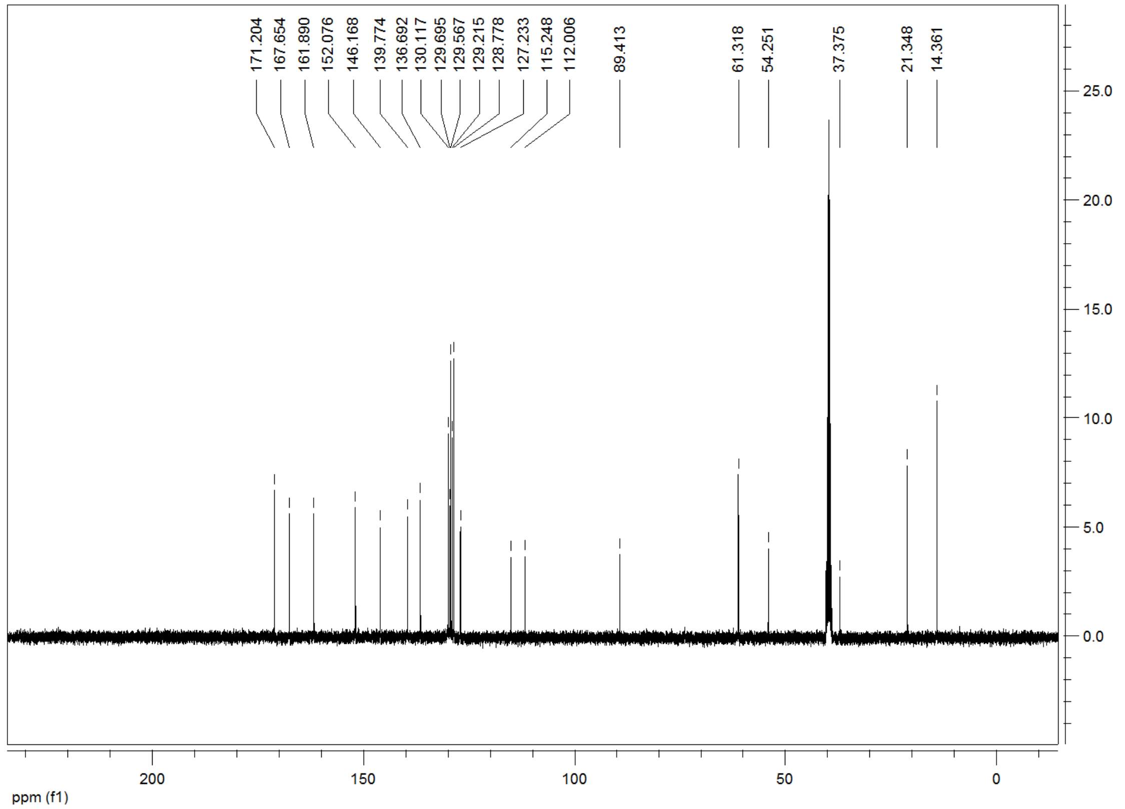
171.204
167.654
161.890
152.076
146.168
139.774
136.692
130.117
129.695
129.567
129.215
128.778
127.233
115.248
112.006
89.413
61.318
54.251
37.375
21.348
14.361
ppm (f1)

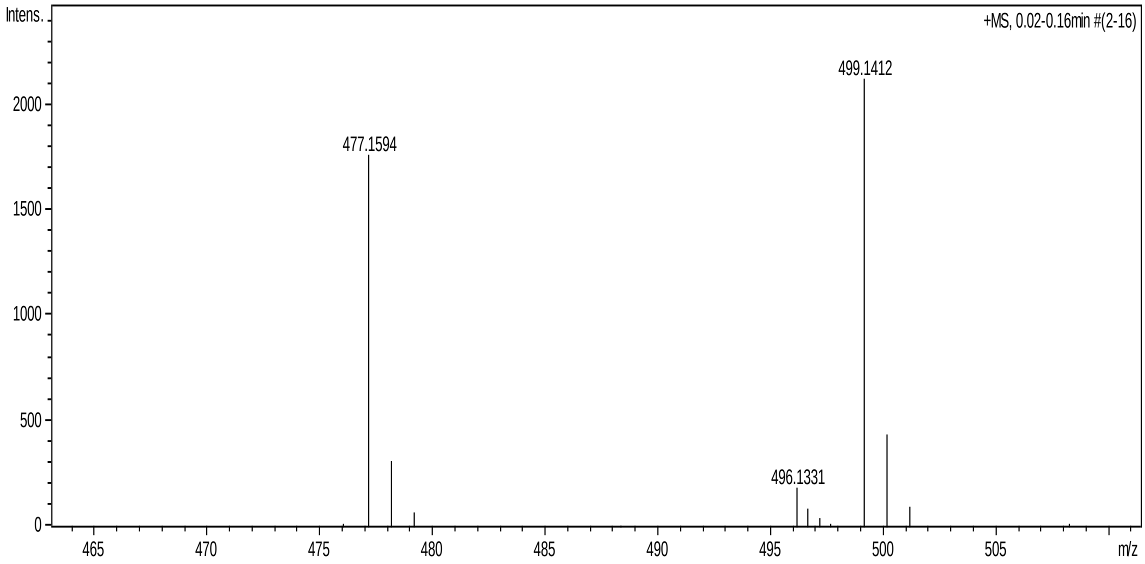
Intens.
+MS, 0.02-0.16min #(2-16)
499.1412
477.1594
496.1331
m/z

ethyl ((5-amino-3-(4-chlorophenyl)-4-cyanothiophene-2-carbonyl)carbamoyl)phenylalaninate (3e): yellow solid, 63%, m.p. 178-180℃; ^{1}H NMR (400 MHz, DMSO-d_6) δ: 8.89 (s, 1H, NH), 8.47 (d, J = 6.4 Hz, 1H, NH), 8.13 (s, 2H, NH_2), 7.56 (d, J = 7.6 Hz, 2H, ArH), 7.40 (d, J = 8.0 Hz, 2H, ArH), 7.28-7.23 (m, 3H, ArH), 7.13 (d, J = 6.0 Hz, 2H, ArH), 4.51-4.48 (m, 1H, CH), 4.08-4.04 (m, 2H, CH), 3.02-3.01 (m, 2H, CH), 1.13 (t, J = 6.8 Hz, 3H, CH_3); ^{13}C NMR (100 MHz, DMSOd6) δ: 171.2, 167.3, 162.1, 152.4, 144.7, 136.7, 134.5, 131.8, 131.3, 129.5, 129.1, 128.7, 127.2, 115.2, 112.1, 88.7, 61.3, 54.2, 37.3, 14.3; IR (KBr) υ: 3383, 3300, 2980, 2212, 1743, 1690, 1645, 1473, 1408, 1314, 1261, 1186, 1084, 1021, 845cm^{-1}; MS (m/z): HRMS (ESI) Calcd. for $C_{24}H_{21}ClN_4NaO_4S$ ($[M+Na]^+$): 519.0870, found: 519.0878.

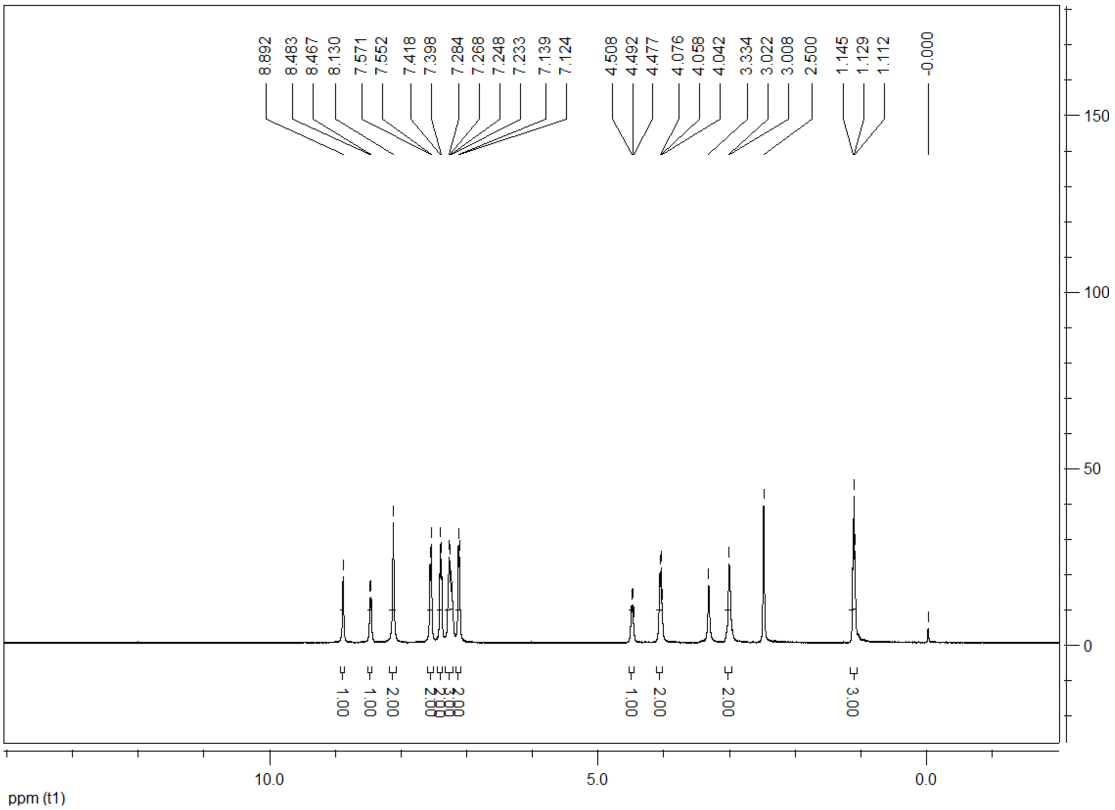

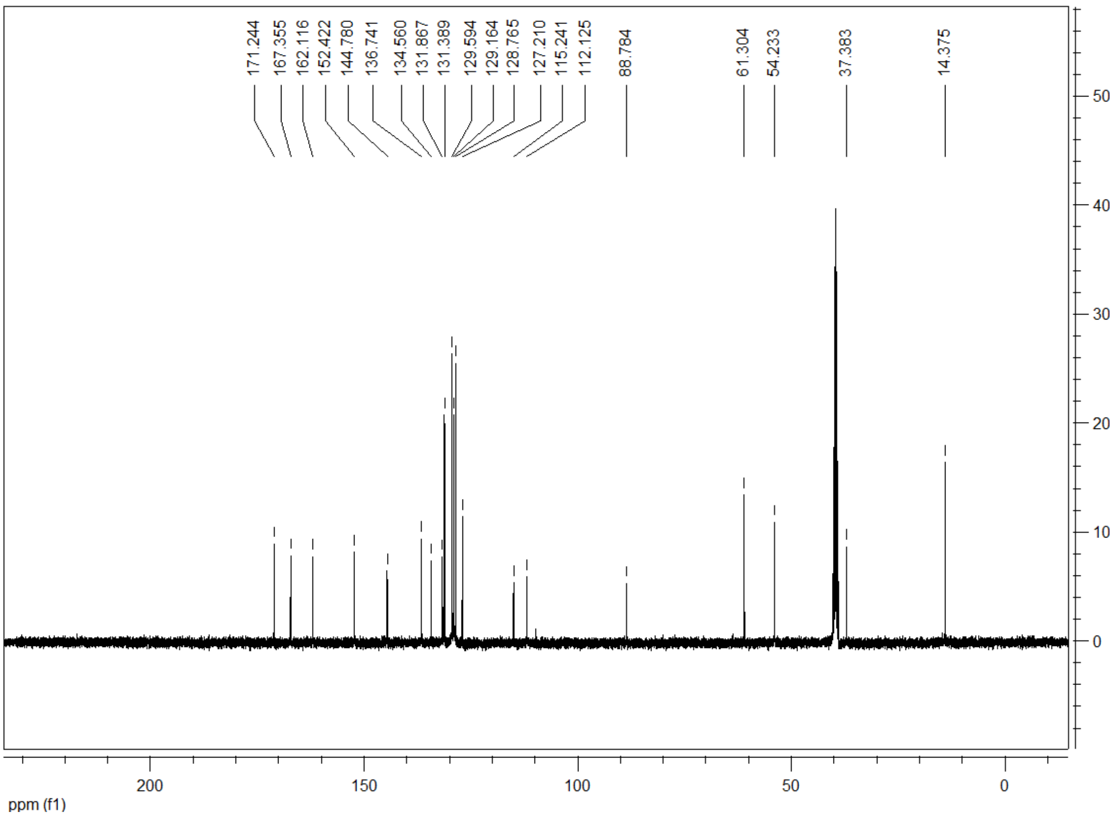
171.244
167.355
162.116
152.422
144.780
136.741
134.560
131.867
131.389
129.594
129.164
128.765
127.210
115.241
112.125
88.784
61.304
54.233
37.383
14.375
ppm (f1)

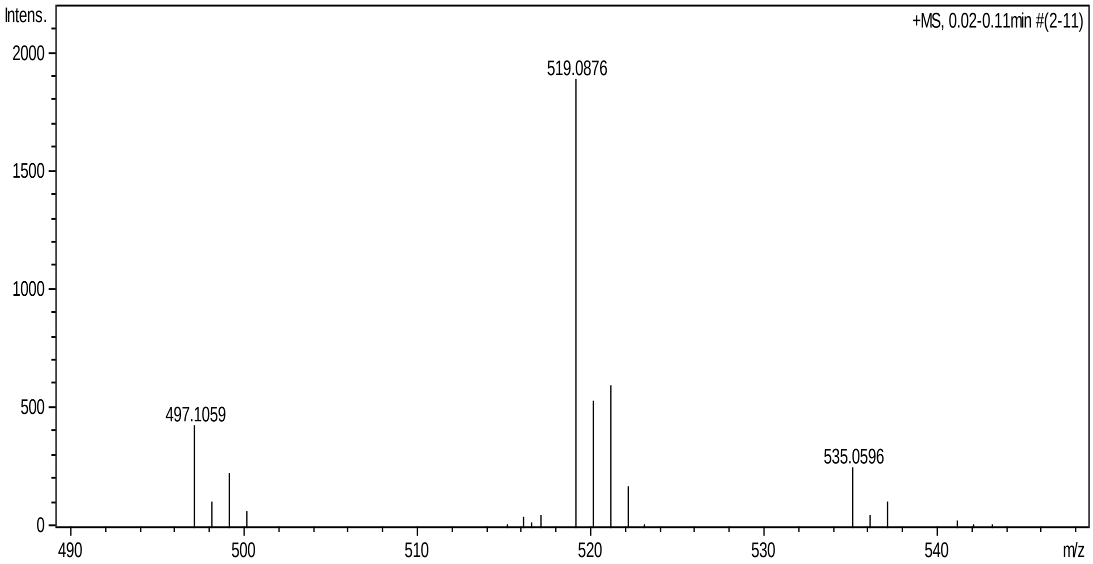
Intens.
+MS, 0.02-0.11min #(2-11)
519.0876
497.1059
535.0596
m/z

ethyl ((5-amino-3-(4-bromophenyl)-4-cyanothiophene-2-carbonyl)carbamoyl)phenylalaninate (3f): yellow solid, 55%, m.p. 156-158℃; ^{1}H NMR (400 MHz, DMSO-d_6) δ: 8.93 (s, 1H, NH), 8.47 (d, J = 7.2 Hz, 1H, NH), 8.12 (s, 2H, NH_2), 7.69 (d, J = 7.6 Hz, 2H, ArH), 7.33 (d, J = 8.0 Hz, 2H, ArH), 7.30-7.23 (m, 3H, ArH), 7.13 (d, J = 6.8 Hz, 2H, ArH), 4.50 (d, J = 6.8 Hz, 1H, CH), 4.07 (q, J = 7.2 Hz, 2H, CH), 3.04-3.01 (m, 2H, CH), 1.13 (t, J = 6.8 Hz, 3H, CH_3); ^{13}C NMR (100 MHz, DMSOd6) δ: 170.7, 166.9, 161.6, 151.9, 144.3, 136.2, 131.8, 131.6, 131.1, 129.1, 128.3, 126.7, 122.7, 114.7, 111.6, 88.2, 60.8, 53.7, 36.9, 13.9; IR (KBr) υ: 3383, 3300, 3199, 2922, 2213, 1691, 1646, 1474, 1411, 1317, 1262, 1186, 1077, 1017, 839 cm^{-1}; MS (m/z): HRMS (ESI) Calcd. for $C_{24}H_{21}BrN_4O_4S$ ($[M+H]^+$): 541.0467, found: 541.0542.

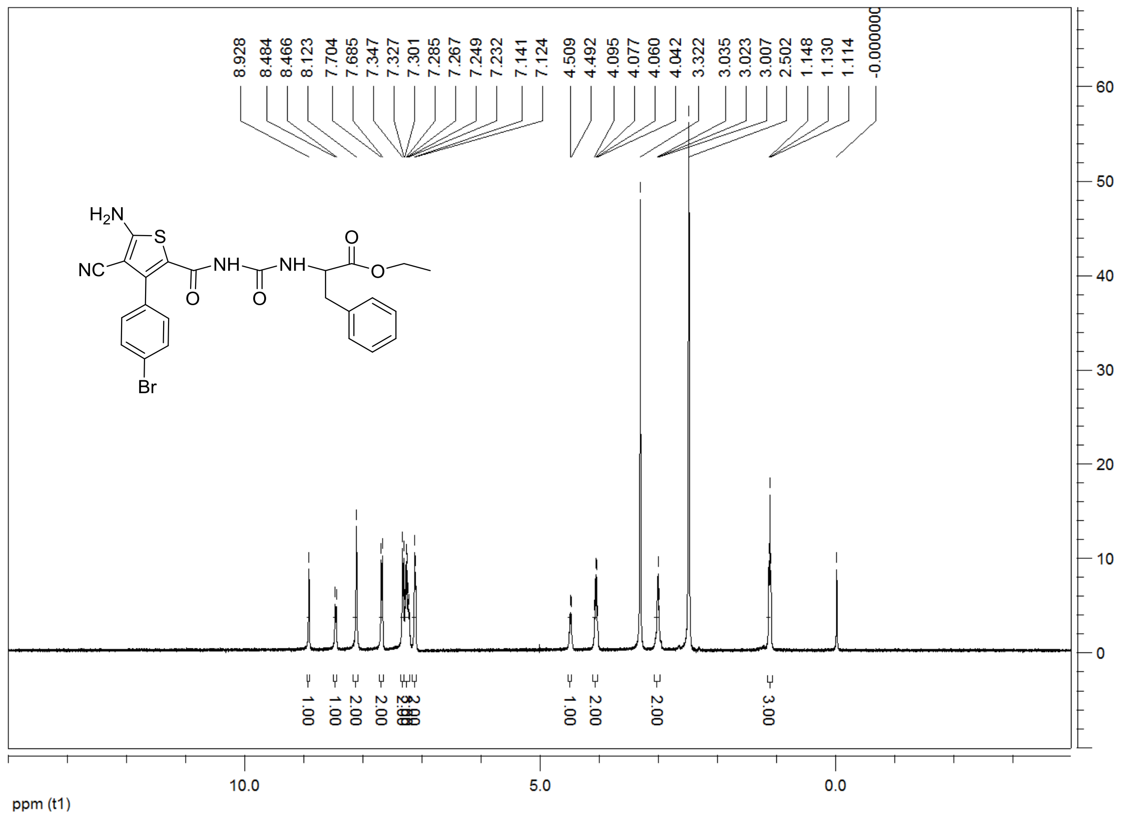

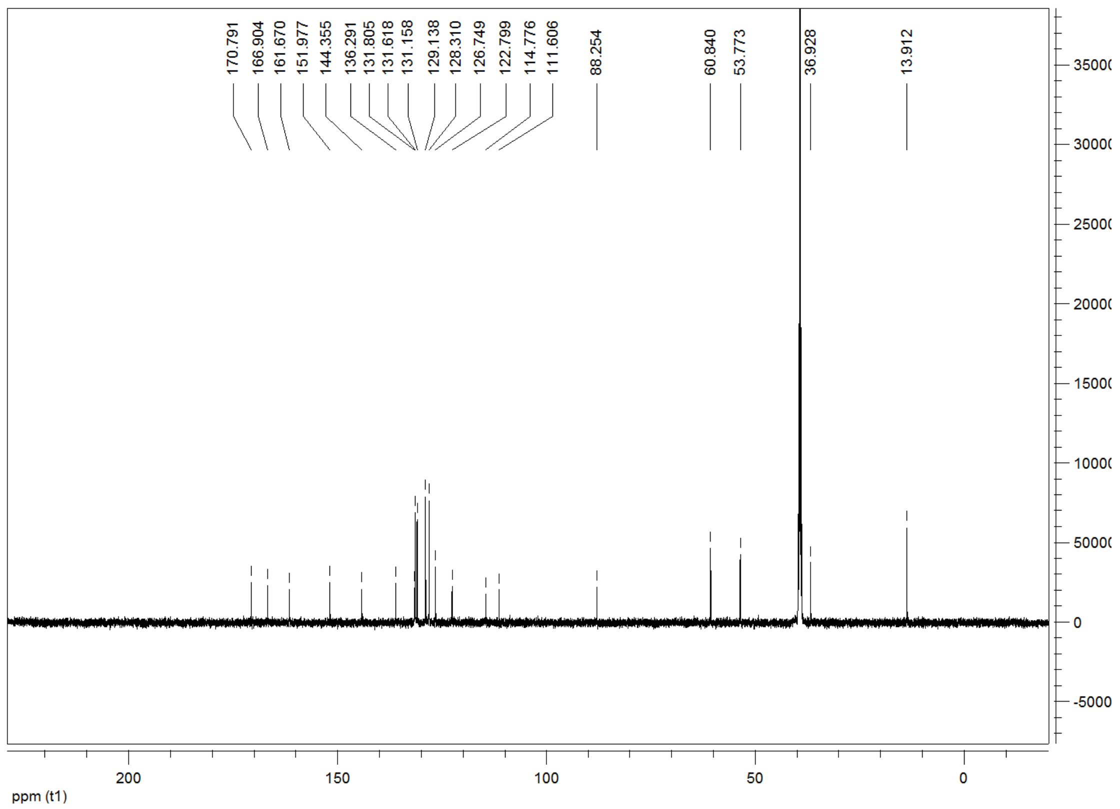
170.791
166.904
161.670
151.977
144.355
136.291
131.805
131.618
131.158
129.138
128.310
126.749
122.799
114.776
111.606
88.254
60.840
53.773
36.928
13.912
200
150
100
50
0
ppm (t1)

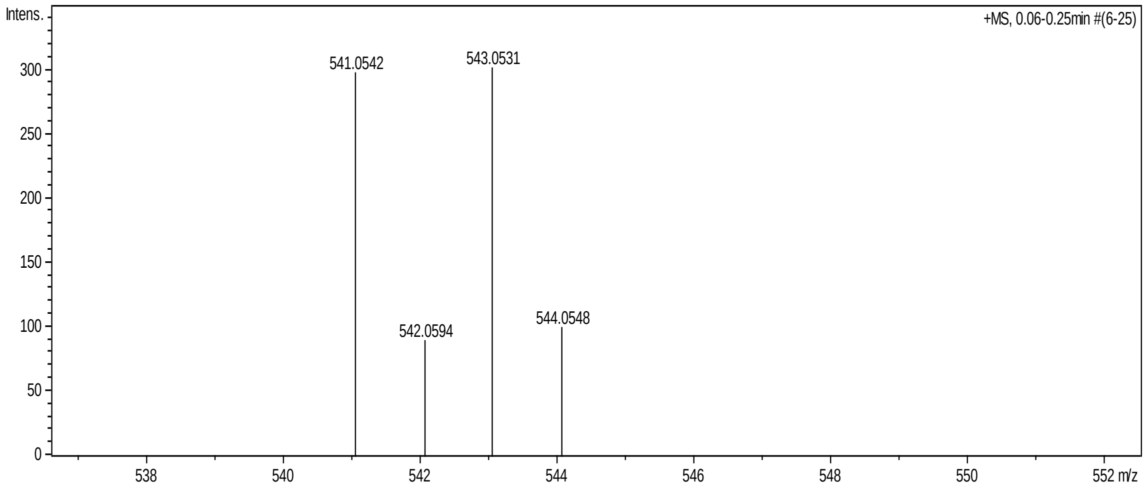
Intens.
+MS, 0.06-0.25min #(6-25)
541.0542
543.0531
542.0594
544.0548
300
250
200
150
100
50
0
538
540
542
544
546
548
550
552 m/z

ethyl ((5-amino-4-cyano-3-(p-tolyl)thiophene-2-carbonyl)carbamoyl)glycinate (3g): yellow solid, 60%, m.p. 234-236℃; ^{1}H NMR (400 MHz, DMSO-d_6) δ: 8.47 (t, J = 5.6 Hz, 1H, NH), 8.22 (s, 1H, NH), 8.10 (s, 2H, NH_2), 7.35-7.31 (m, 4H, ArH), 4.09-4.04 (q, J = 7.2 Hz, 2H, CH), 3.85 (d, J = 5.6 Hz, 2H, CH), 2.37 (s, 3H, CH_3), 1.16 (t, J = 7.2 Hz, 3H, CH_3); ^{13}C NMR (100 MHz, DMSOd6) δ: 170.0, 167.5, 161.6, 152.7, 146.1, 139.6, 130.0, 129.8, 129.2, 115.2, 112.1, 89.3, 60.9, 41.7, 21.3, 14.4; IR (KBr) υ: 3376, 3331, 3225, 2920, 2853, 2210, 1708, 1640, 1519, 1478, 1404, 1319, 1266, 1202, 1078, 1025, 864, 824 cm^{-1}; MS (m/z): HRMS (ESI) Calcd. for $C_{18}H_{18}N_4O_4S$ ($[M+Na]^+$): 409.1049, found: 409.0939.

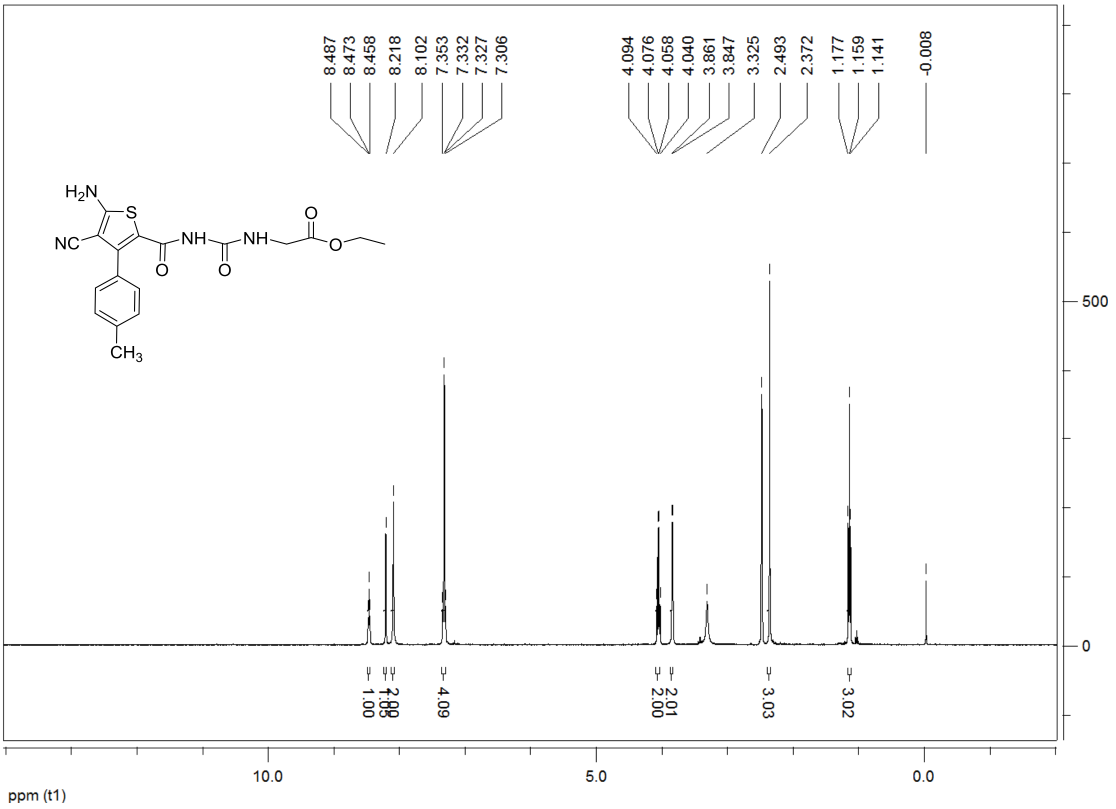

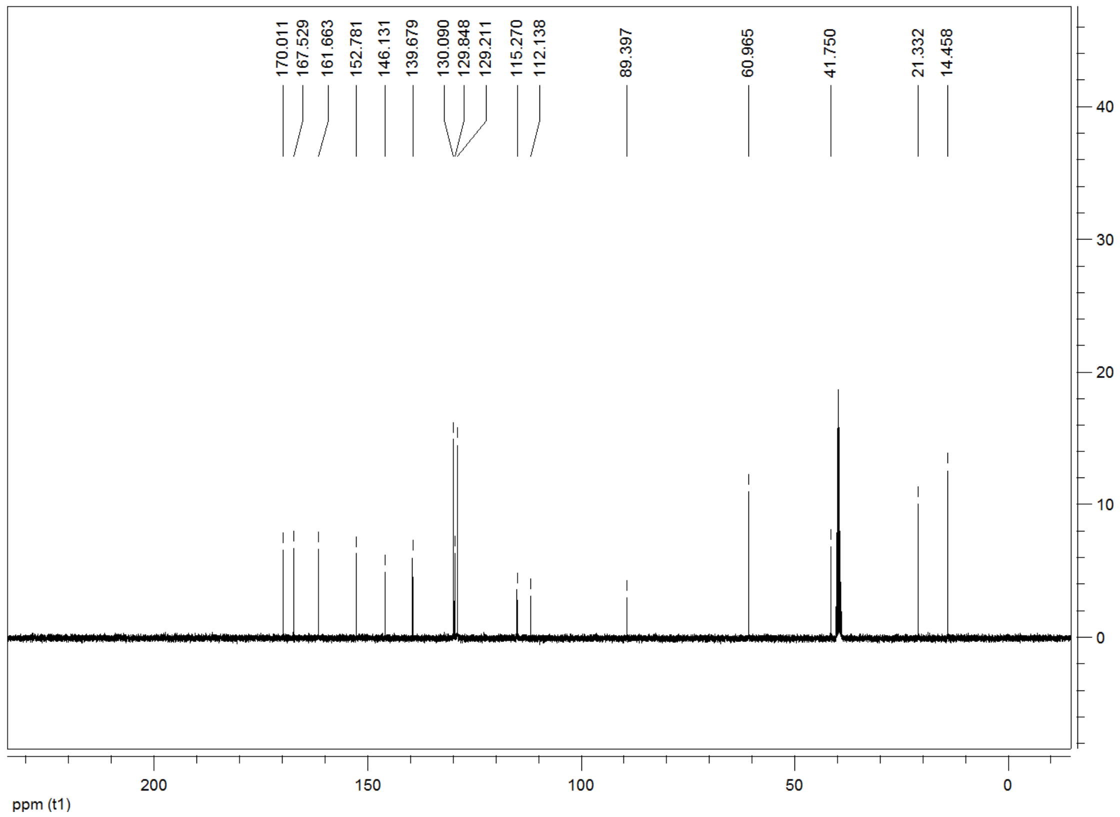
170.011
167.529
161.663
152.781
146.131
139.679
130.090
129.848
129.211
115.270
112.138
89.397
60.965
41.750
21.332
14.458
40
30
20
10
0
200
150
100
50
0
ppm (t1)

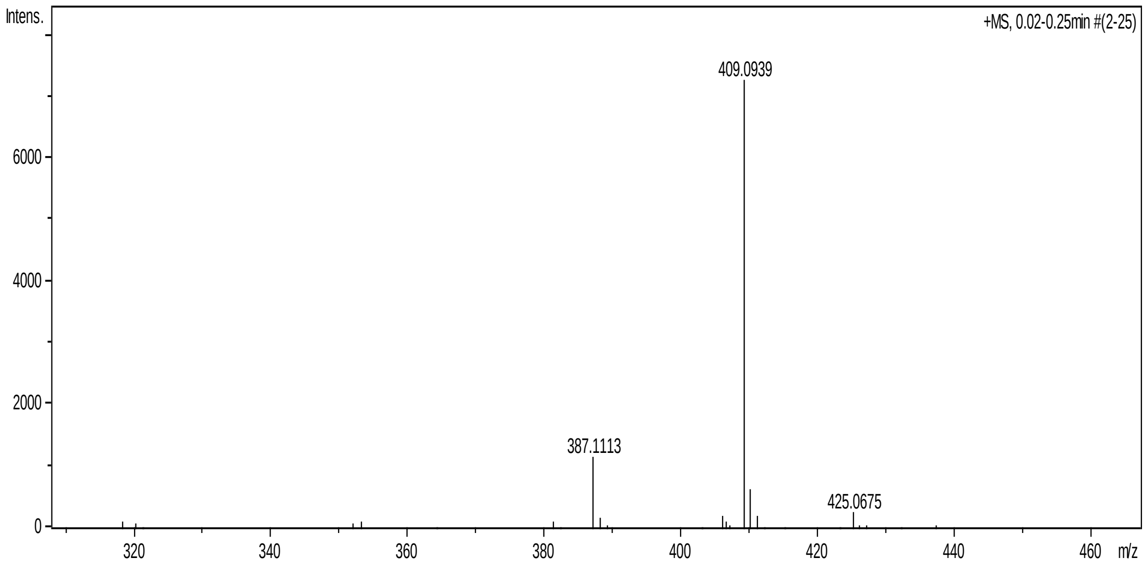
Intens.
+MS, 0.02-0.25min #(2-25)
409.0939
387.1113
425.0675
6000
4000
2000
0
320
340
360
380
400
420
440
460
m/z

ethyl ((5-amino-4-cyano-3-(4-methoxyphenyl)thiophene-2-carbonyl)carbamoyl)alaninate (3h): yellow solid, 68%, m.p. 186-188℃; ^{1}H NMR (400 MHz, DMSO-d_6) δ: 8.51 (d, J = 7.2 Hz, 1H, NH), 8.13 (s, 3H, NH, NH_2), 7.38 (d, J = 8.4 Hz, 2H, ArH), 7.09 (d, J = 8.4 Hz, 2H, ArH), 4.24-4.20 (m, 1H, CH), 4.12-4.07 (m, 2H, CH), 3.83 (s, 3H, OCH_3), 1.30 (d, J = 7.2 Hz, 3H, CH_3), 1.17 (t, J = 7.2 Hz, 3H, CH_3); ^{13}C NMR (100 MHz, DMSOd6) δ: 172.4, 167.5, 161.9, 160.5, 152.0, 146.1, 131.0, 130.6, 124.5, 115.3, 111.8, 89.5, 61.2, 55.8, 48.7, 17.8, 14.2; IR (KBr) υ: 3377, 3286, 3172, 2938, 2215, 1740, 1687, 1653, 1514, 1476, 1415, 1320, 1255, 1210, 1177, 1095, 1019, 845 cm^{-1}; MS (m/z): HRMS (ESI) Calcd. for $C_{19}H_{20}N_4O_5S$ ($[M+Na]^+$): 439.1154, found: 439.1048.

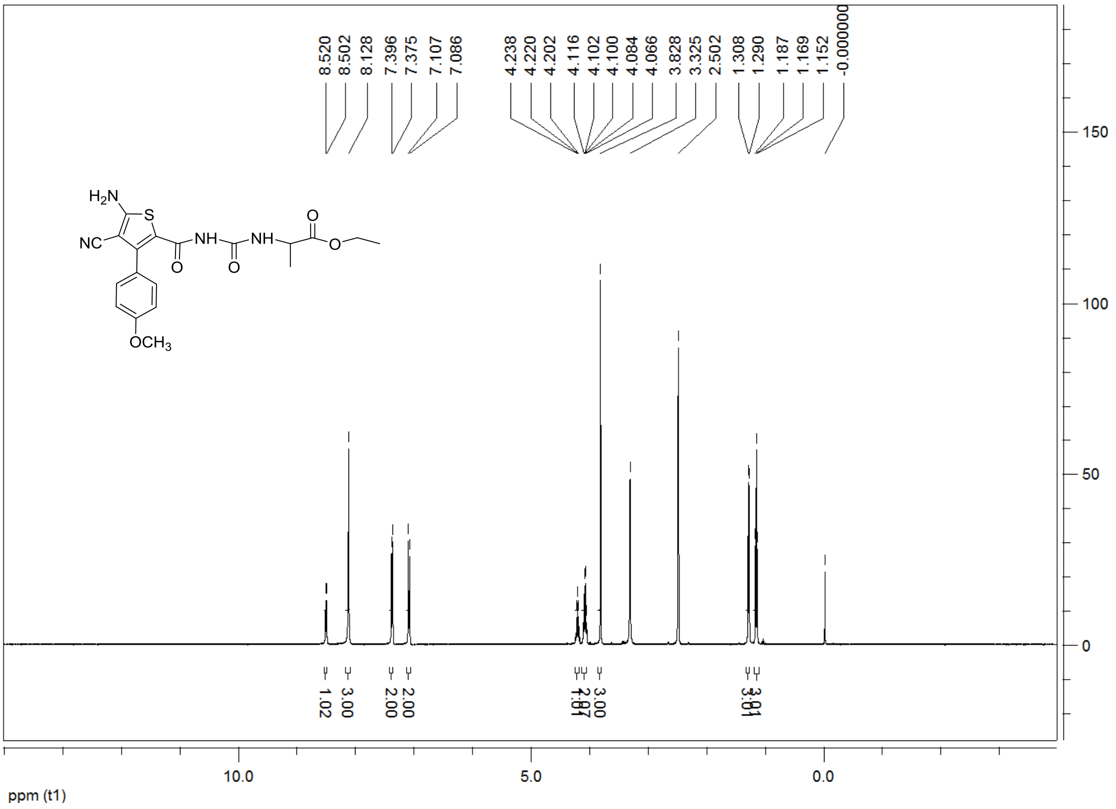

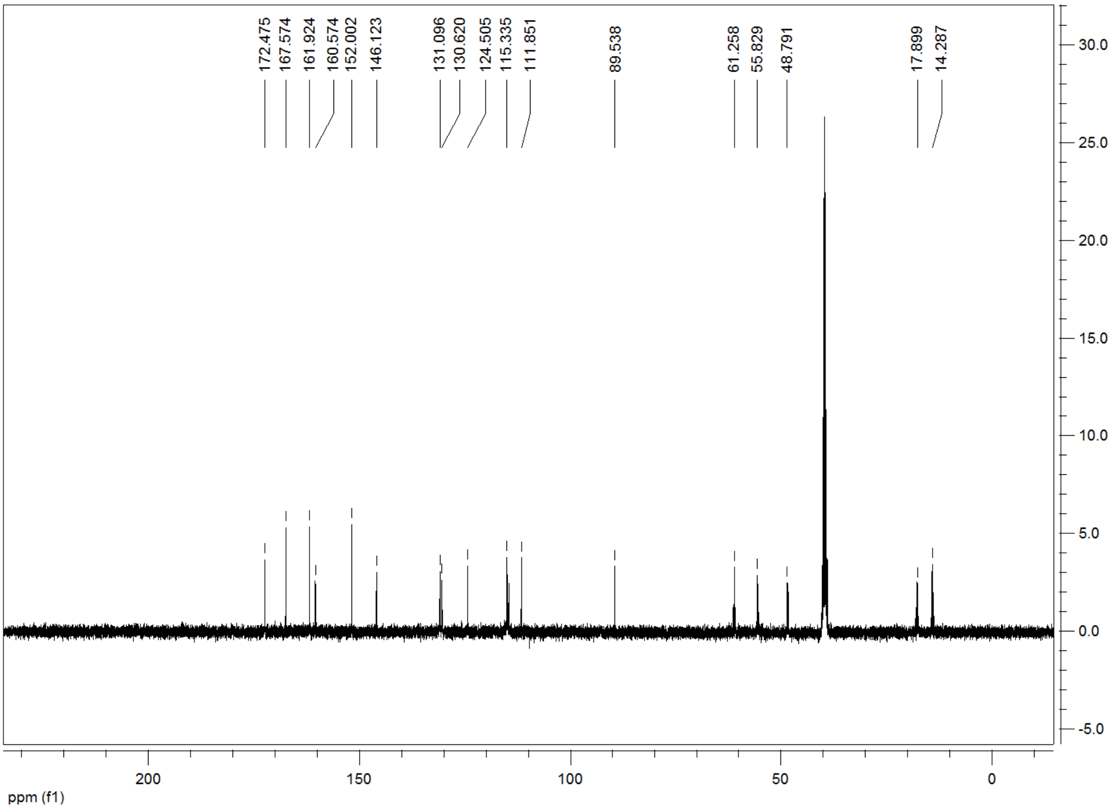

172.475
167.574
161.924
160.574
152.002
146.123
131.096
130.620
124.505
115.335
111.851
89.538
61.258
55.829
48.791
17.899
14.287
30.0
25.0
20.0
15.0
10.0
5.0
0.0
-5.0
200
150
100
50
0
ppm (f1)

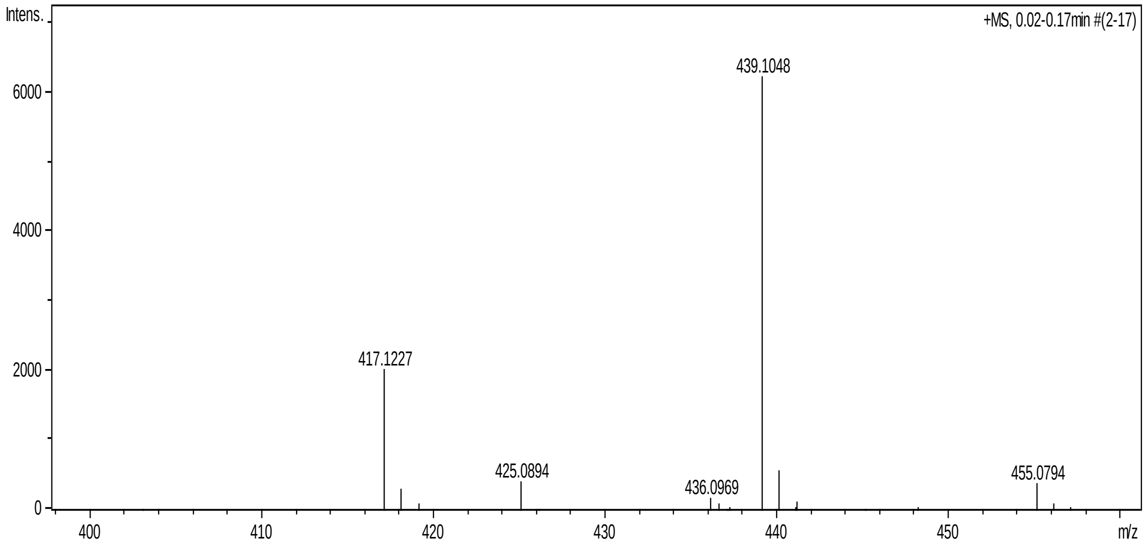

Intens.
+MS, 0.02-0.17min #(2-17)
439.1048
417.1227
425.0894
436.0969
455.0794
6000
4000
2000
0
400
410
420
430
440
450
m/z

Ethyl 2-(3-(5-amino-4-cyano-3-(4-methoxyphenyl)thiophene-2-carbonyl)ureido)-4-phenylbutanoate (3i): yellow solid, 58%, m.p. 180-182℃; ^{1}H NMR (400 MHz, DMSO-d_6) δ: 8.58 (d, J = 7.2 Hz, 1H, NH), 8.15 (s, 3H, NH, NH_2), 7.40 (d, J = 8.4 Hz, 2H, ArH), 7.29-7.25 (m, 2H, ArH), 7.20-7.16 (m, 3H, ArH), 7.09 (d, J = 8.4 Hz, 2H, ArH), 4.23-4.18 (m, 1H, CH), 4.11-4.05 (m, 2H, CH), 3.82 (s, 3H, OCH_3), 2.58-2.54 (m, 2H, CH), 2.04-1.94 (m, 2H, CH), 1.16 (t, J = 7.2 Hz, 3H, CH_3); ^{13}C NMR (100 MHz, DMSOd6) δ: 171.8, 167.6, 162.0, 160.6, 152.2, 146.0, 140.9, 130.8, 128.7, 128.7, 126.4, 124.4, 115.3, 114.9, 111.9, 89.5, 61.3, 55.7, 52.4, 33.3, 31.3, 14.4; IR (KBr) υ: 3399, 3295, 3209, 2923, 2850, 2202 1702, 1687, 1641, 1504, 1476, 1478, 1311, 1262, 1223, 1178, 1088, 1029, 955, 873, 834 cm^{-1}; MS (m/z): HRMS (ESI) Calcd. for $C_{26}H_{26}N_4NaO_5S$ ($[M+Na]^+$): 529.1516, found: 529.1526.

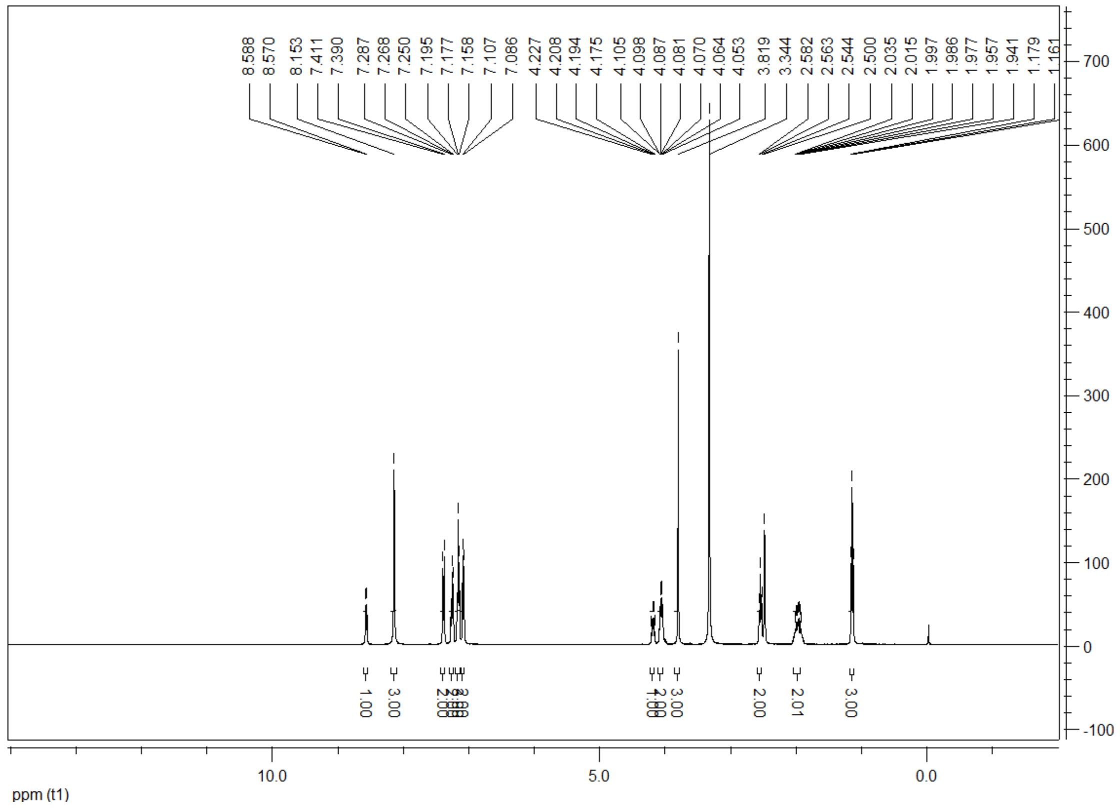

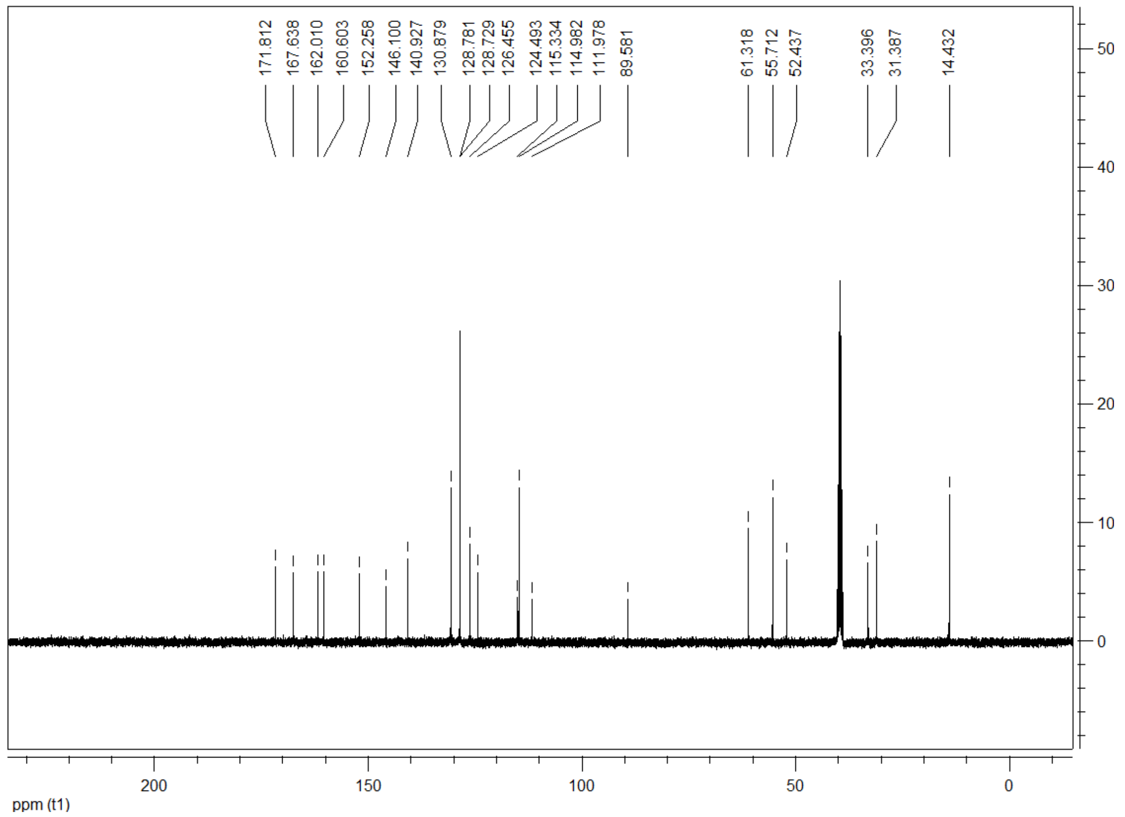
171.812
167.638
162.010
160.603
152.258
146.100
140.927
130.879
128.781
128.729
126.455
124.493
115.334
114.982
111.978
89.581
61.318
55.712
52.437
33.396
31.387
14.432
ppm (t1)

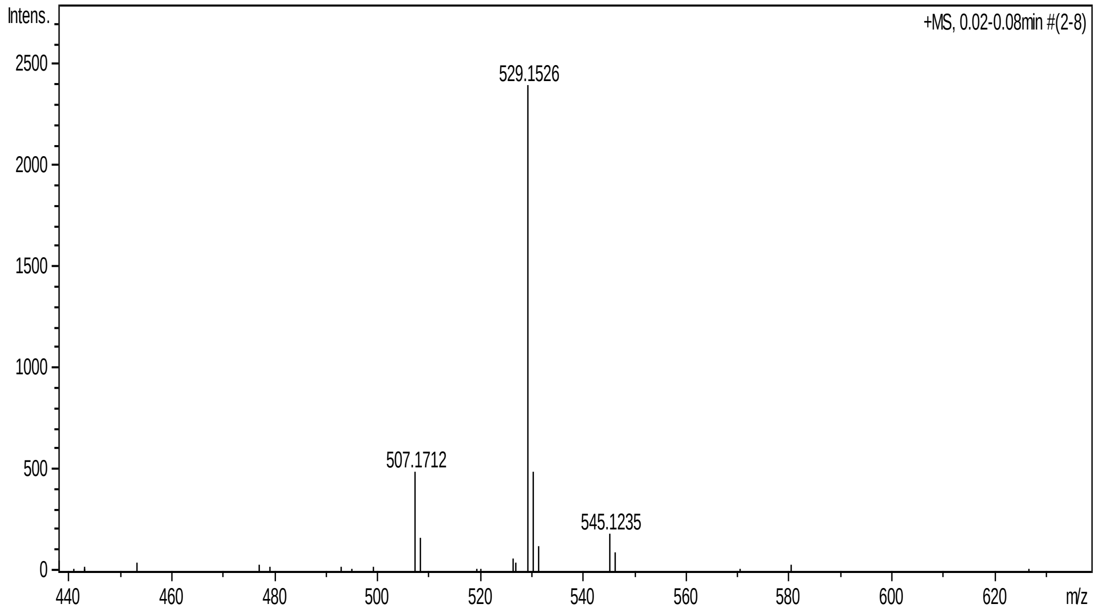
Intens.
+MS, 0.02-0.08min #(2-8)
529.1526
507.1712
545.1235
m/z

ethyl ((5-amino-4-cyano-3-(4-methoxyphenyl)thiophene-2-carbonyl)carbamoyl)tyrosinate (3j): yellow solid, 66%, m.p. 186-188℃; ^{1}H NMR (400 MHz, DMSO-d_6) δ: 9.29 (s, 1H, OH), 8.45 (d, J = 7.6 Hz, 1H, NH), 8.13 (s, 2H, NH_2), 8.10 (s, 1H, NH), 7.36 (d, J = 8.4 Hz, 2H, ArH), 7.08 (d, J = 8.4 Hz, 2H, ArH), 6.91 (d, J = 8.0 Hz, 2H, ArH), 6.66 (d, J = 8.0 Hz, 2H, ArH), 4.42-4.37 (m, 1H, CH), 4.09-4.03 (m, 2H, CH), 3.82 (s, 3H, OCH_3), 2.92-2.85 (m, 2H, CH), 1.14 (t, J = 7.2 Hz, 3H, CH_3); ^{13}C NMR (100 MHz, DMSO-d_6) δ: 171.3, 167.5, 161.9, 160.5, 156.6, 152.0, 145.9, 130.8, 130.5, 126.5, 124.4, 115.5, 115.3, 114.9, 111.9, 89.4, 61.2, 55.7, 54.4, 36.6, 14.4; IR (KBr) υ: 3433, 3373, 3290, 3209, 3181, 2936, 2850, 2212 1702, 1681, 1643, 1516, 1470, 1410, 1316, 1262, 1256, 1185, 1088, 1077, 1022, 838 cm^{-1}; MS (m/z): HRMS (ESI) Calcd. for $C_{25}H_{24}N_4NaO_6S$ ([M+Na]$^+$): 531.1309, found: 531.1319.

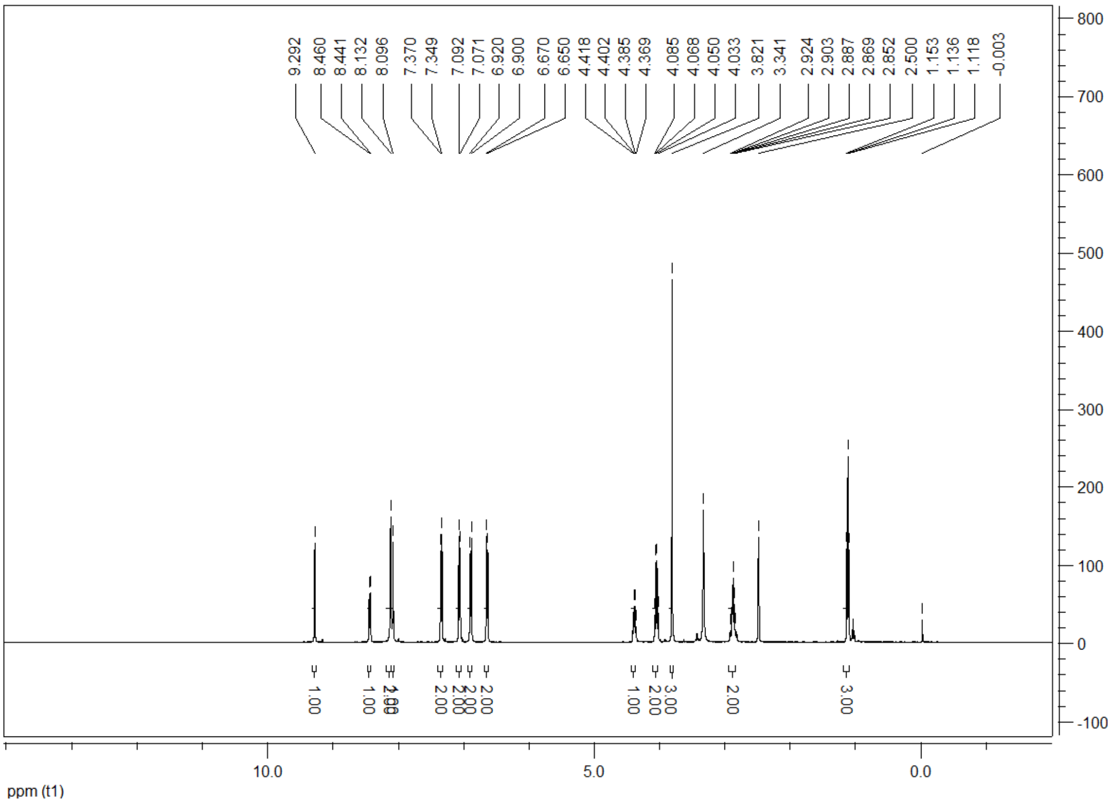

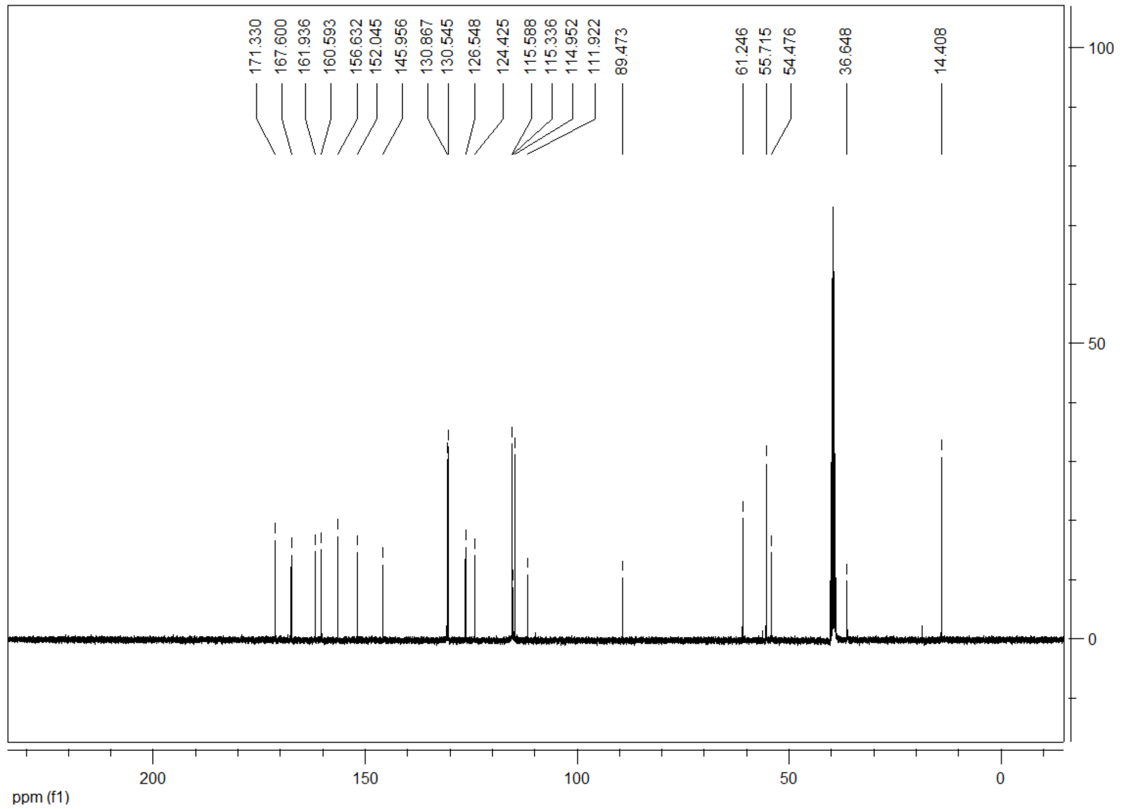
171.330
167.600
161.936
160.593
156.632
152.045
145.956
130.867
130.545
126.548
124.425
115.588
115.336
114.952
111.922
89.473
61.246
55.715
54.476
36.648
14.408
100
50
0
200
150
100
50
0
ppm (f1)

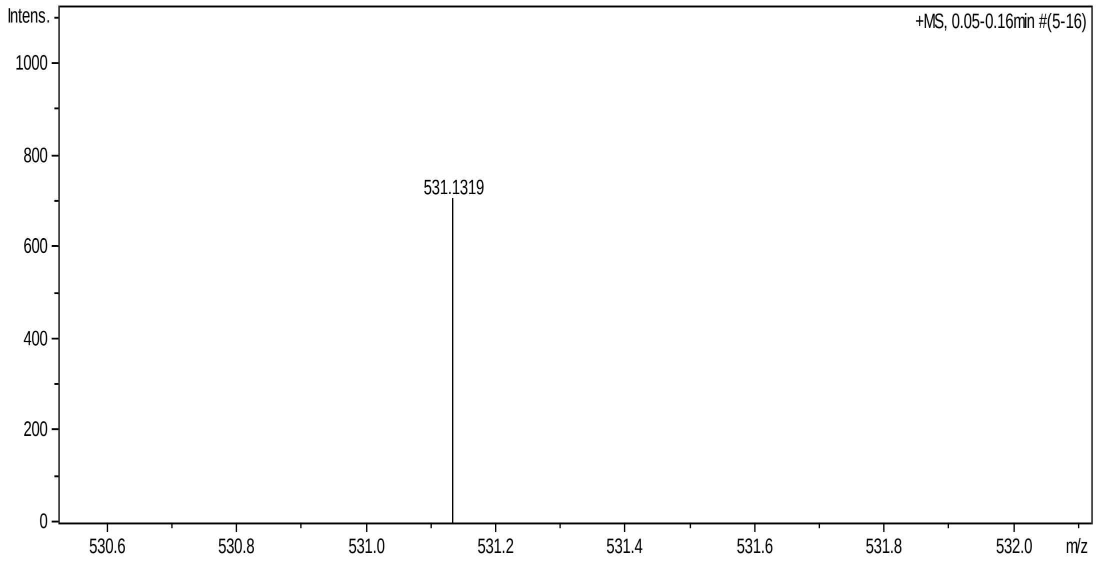
Intens.
+MS, 0.05-0.16min #(5-16)
1000
800
600
400
200
0
531.1319
530.6
530.8
531.0
531.2
531.4
531.6
531.8
532.0
m/z
